# Supplementary material for: Antimicrobial Susceptibility in Respiratory Pathogens and Farm and Animal Variables in Weaned California Dairy Heifers: Logistic Regression and Bayesian Network Analyses
Source: Antibiotics (Basel). 2024 Jan 4;13(1):50. doi: 10.3390/antibiotics13010050 (PMC10812578; doi:10.3390/antibiotics13010050)
Supplement: Supplementary file 1 [file antibiotics-13-00050-s001.zip › Supplemenatry File S2.html]

BRD Pathogens - BNA Manuscript


# BRD Pathogens - BNA Manuscript

#### Brittany L Morgan Bustamante

#### 12/09/2022

# 1. *P. multocida*

### Farm level factors

First analysis is an assessment of farm-level factors on *P.
multocida* resistance/susceptibile MIC interpretations. Variables at
this level under consideration include type of calf rearing facility
(single source, multisource), whether the farm practices onsite milking,
vaccines or biologicals administered to calves on site, colostrum
administration at arrival to multisource facility, colostrum
pasteurization, hospital milk pasteurization, bull calves comingled with
heifers, milk mass medication, grain mass medication, water mass
medication, feed lane cleaning method in weaned pens (lagoon flush,
scrape, clean water flush).

Any variable with a cell size less than 10 will be recategorized, if
possible. If it isn’t possible to recategorize to meet a minimum count
of 10, the variable will be removed from analysis.

#### 1.1 Data Wrangling

```
pmult.df <- read_csv("pmult.csv", show_col_types = FALSE)

# connect calf treatment variables to pmult df
pmult.raw <- left_join(pmult.df, calf.trx, by = "calf_ID")


# make variables factors, add labels
pmult.factor = pmult.raw
pmult.factor$season <- factor(pmult.raw$season, order = FALSE,
                              levels = c(1, 2),
                              labels = c("summer", "winter"))
pmult.factor$brd <- factor(pmult.raw$brd, order = FALSE,
                           levels = c(0, 1),
                           labels = c("neg", "pos"))
pmult.factor$cef <- factor(pmult.raw$cef, order = FALSE,
                           levels = c(0, 1),
                           labels = c("S", "R"))
pmult.factor$dan <- factor(pmult.raw$dan, order = FALSE,
                           levels = c(0, 1),
                           labels = c("S", "R"))
pmult.factor$enr <- factor(pmult.raw$enr, order = FALSE,
                           levels = c(0, 1),
                           labels = c("S", "R"))
pmult.factor$flr <- factor(pmult.raw$flr, order = FALSE,
                         levels = c(0, 1),
                         labels = c("S", "R"))
pmult.factor$gam <- factor(pmult.raw$gam, order = FALSE,
                           levels = c(0, 1),
                           labels = c("S", "R"))
pmult.factor$gen <- factor(pmult.raw$gen, order = FALSE,
                           levels = c(0, 1),
                           labels = c("S", "R"))
pmult.factor$pen <- factor(pmult.raw$pen, order = FALSE,
                           levels = c(0, 1),
                           labels = c("S", "R"))
pmult.factor$spc <- factor(pmult.raw$spc, order = FALSE,
                           levels = c(0, 1),
                           labels = c("S", "R"))
pmult.factor$tet <- factor(pmult.raw$tet, order = FALSE,
                           levels = c(0, 1),
                           labels = c("S", "R"))
pmult.factor$tild <- factor(pmult.raw$tild, order = FALSE,
                            levels = c(0, 1),
                            labels = c("S", "R"))
pmult.factor$tilm <- factor(pmult.raw$tilm, order = FALSE,
                            levels = c(0, 1),
                            labels = c("S", "R"))
pmult.factor$tul <- factor(pmult.raw$tul, order = FALSE,
                           levels = c(0, 1),
                           labels = c("S", "R"))
pmult.factor$source_no <- factor(pmult.raw$source_no, order = FALSE,
                                 levels = c(1, 2, 3),
                                 labels = c("single", "low multi", "high multi"))
pmult.factor$onsite_milking <- factor(pmult.raw$onsite_milking, order = FALSE,
                                      levels = c(0, 1),
                                      labels = c("no", "yes"))
pmult.factor$resp_vacc <- factor(pmult.raw$resp_vacc, order = FALSE,
                                 levels = c(0, 1),
                                 labels = c("no", "yes"))
pmult.factor$salmonella <- factor(pmult.raw$salmonella, order = FALSE,
                           levels = c(0, 1),
                           labels = c("no", "yes"))
pmult.factor$first_def <- factor(pmult.raw$first_def, order = FALSE,
                                 levels = c(0, 1),
                                 labels = c("no", "yes"))
pmult.factor$bovishield <- factor(pmult.raw$bovishield, order = FALSE,
                                  levels = c(0, 1),
                                  labels = c("no", "yes"))
pmult.factor$pyramid <- factor(pmult.raw$pyramid, order = FALSE,
                               levels = c(0, 1),
                               labels = c("no", "yes"))
pmult.factor$titanium <- factor(pmult.raw$titanium, order = FALSE,
                                levels = c(0, 1),
                                labels = c("no", "yes"))
pmult.factor$pinkeye <- factor(pmult.raw$pinkeye, order = FALSE,
                               levels = c(0, 1),
                               labels = c("no", "yes"))
pmult.factor$clostrid <- factor(pmult.raw$clostrid, order = FALSE,
                                levels = c(0, 1),
                                labels = c("no", "yes"))
pmult.factor$colost_source <- factor(pmult.raw$colost_source, order = FALSE,
                                     levels = c(1, 2),
                                     labels = c("p", "dp"))
pmult.factor$colost_past <- factor(pmult.raw$colost_past, order = FALSE,
                                   levels = c(0, 1),
                                   labels = c("no", "yes"))
pmult.factor$colost_again <- factor(pmult.raw$colost_again, order = FALSE,
                                    levels = c(0, 1, 99),
                                    labels = c("no", "yes", "-99"))
pmult.factor$hosp_milk_pastuerized <- factor(pmult.raw$hosp_milk_pastuerized, order = FALSE,
                                             levels = c(0, 1),
                                             labels = c("no", "yes"))
pmult.factor$action <- factor(pmult.raw$action, order = FALSE,
                              levels = c(1, 2),
                              labels = c("screen", "premium"))
pmult.factor$bull_calves <- factor(pmult.raw$bull_calves, order = FALSE,
                                   levels = c(0, 1),
                                   labels = c("no", "yes"))
pmult.factor$milk_mass_med <- factor(pmult.raw$milk_mass_med, order = FALSE,
                                     levels = c(0, 1),
                                     labels = c("no", "yes"))
pmult.factor$mass_pen_exp <- factor(pmult.raw$mass_pen_exp, order = FALSE,
                                    levels = c(0, 1),
                                    labels = c("no", "yes"))
pmult.factor$mass_tet_exp <- factor(pmult.raw$mass_tet_exp, order = FALSE,
                                    levels = c(0, 1),
                                    labels = c("no", "yes"))
pmult.factor$mass_corr_exp <- factor(pmult.raw$mass_corr_exp, order = FALSE,
                                    levels = c(0, 1),
                                    labels = c("no", "yes"))
pmult.factor$mass_rum_exp <- factor(pmult.raw$mass_rum_exp, order = FALSE,
                                    levels = c(0, 1),
                                    labels = c("no", "yes"))
pmult.factor$mass_sul_exp <- factor(pmult.raw$mass_sul_exp, order = FALSE,
                                    levels = c(0, 1),
                                    labels = c("no", "yes"))
pmult.factor$feed_lane_cleaning <- factor(pmult.raw$feed_lane_cleaning, order = FALSE,
                                          levels = c(1, 2, 3, 4),
                                          labels = c("scrape", "lagoon", "clean", "sl"))
pmult.factor$dust_mgmt <- factor(pmult.raw$dust_mgmt, order = FALSE,
                                 levels = c(0, 1),
                                 labels = c("no", "yes"))
pmult.factor$mort_rate <- factor(pmult.raw$mort_rate, order = FALSE,
                                 levels = c(0, 1),
                                 labels = c("no", "yes"))
pmult.factor$pen_trx_rate <- factor(pmult.raw$pen_trx_rate, order = FALSE,
                                    levels = c(1, 2),
                                    labels = c("low", "high"))
pmult.factor$cef_trx_rate <- factor(pmult.raw$cef_trx_rate, order = FALSE,
                                    levels = c(1, 2),
                                    labels = c("low", "high"))
pmult.factor$mac_trx_rate <- factor(pmult.raw$mac_trx_rate, order = FALSE,
                                    levels = c(1, 2),
                                    labels = c("low", "high"))
pmult.factor$res_nuf_trx_rate <- factor(pmult.raw$res_nuf_trx_rate, order = FALSE,
                                        levels = c(1, 2),
                                        labels = c("low", "high"))
pmult.factor$tet_inj_use <- factor(pmult.raw$tet_inj_use, order = FALSE,
                                  levels = c(0, 1),
                                  labels = c("no", "yes"))
pmult.factor$sul_trx_rate <- factor(pmult.raw$sul_trx_rate, order = FALSE,
                                    levels = c(1, 2),
                                    labels = c("low", "high"))
pmult.factor$bay_trx_rate <- factor(pmult.raw$bay_trx_rate, order = FALSE,
                                    levels = c(1, 2),
                                    labels = c("low", "high"))
pmult.factor$Farm <- factor(pmult.raw$Farm, order = FALSE,
                            levels = c(1, 2, 3, 4, 5, 6),
                            labels = c("A", "B", "C", "D", "E", "F"))
pmult.factor$last_treat <- factor(pmult.raw$last_treat, order = FALSE,
                                  levels = c(1, 2, 3, 4),
                                  labels = c("<16 days", "16-60 days", ">=60 days", "no trx"))
pmult.factor$no_classes <- factor(pmult.raw$no_classes, order = FALSE,
                                  levels = c(0, 1, 2, 3),
                                  labels = c("0", "1", "2 or more", "2 or more"))
pmult.factor$total_abx <- factor(pmult.raw$total_abx, order = FALSE,
                                  levels = c(0, 1, 2),
                                  labels = c("0", "1 to 2", "3 or more"))
pmult.factor$days_since_pen <- factor(pmult.raw$days_since_pen, order = FALSE,
                                      levels = c(1, 2, 3, 4),
                                      labels = c("<16 days", "16-60 days", ">=60 days", 
                                                 "no trx"))
pmult.factor$days_since_ceph <- factor(pmult.raw$days_since_ceph, order = FALSE,
                                       levels = c(1, 2, 3, 4),
                                       labels = c("<16 days", "16-60 days", ">=60 days", 
                                                  "no trx"))
pmult.factor$days_since_mac <- factor(pmult.raw$days_since_mac, order = FALSE,
                                      levels = c(1, 2, 3, 4),
                                      labels = c("<16 days", "16-60 days", ">=60 days", 
                                                 "no trx"))
pmult.factor$days_since_phen <- factor(pmult.raw$days_since_phen, order = FALSE,
                                       levels = c(1, 2, 3, 4),
                                       labels = c("<16 days", "16-60 days", ">=60 days", 
                                                  "no trx"))
pmult.factor$days_since_tet <- factor(pmult.raw$days_since_tet, order = FALSE,
                                      levels = c(2, 3, 4),
                                      labels = c("16-60 days", ">=60 days", "no trx"))
pmult.factor$days_since_sul <- factor(pmult.raw$days_since_sul, order = FALSE,
                                      levels = c(3, 4),
                                      labels = c(">=60 days", "no trx"))
pmult.factor$days_since_flr <- factor(pmult.raw$days_since_flr, order = FALSE,
                                      levels = c(1, 2, 3, 4),
                                      labels = c("<16 days", "16-60 days", ">=60 days", 
                                                 "no trx"))
                               
# glimpse(pmult.factor)
```

```
# analytic data frame for all farms
pmult.all <- pmult.factor %>%
            select(-"srp", -"entervene", -"pen_trx_rate", -"cef_trx_rate", 
                   -"mac_trx_rate", -"res_nuf_trx_rate", -"brd",
                   -"tet_inj_use", -"sul_trx_rate", -"bay_trx_rate", -"mort_rate", 
                   - "calf_ID", -"Farm_ID", -"cef", -"tet", -"total_abx", -"last_treat", 
                   -"days_since_pen", -"days_since_ceph", -"days_since_mac", 
                   -"days_since_phen", -"days_since_tet", -"days_since_sul", 
                   -"days_since_flr")

# check cell counts - All Farms
summary(pmult.all) # gen, pen, below 10
```

```
##  Farm      season   dan    enr    flr    gam     gen     pen     spc    
##  A:13   summer:71   S:55   S:55   S:59   S: 41   S:139   S:142   S:110  
##  B:24   winter:74   R:90   R:90   R:86   R:104   R:  6   R:  3   R: 35  
##  C:21                                                                   
##  D:34                                                                   
##  E:24                                                                   
##  F:29                                                                   
##  tild    tilm    tul          source_no  onsite_milking resp_vacc first_def
##  S: 36   S: 33   S:120   single    :63   no : 34        no : 24   no :116  
##  R:109   R:112   R: 25   low multi :37   yes:111        yes:121   yes: 29  
##                          high multi:45                                     
##                                                                            
##                                                                            
##                                                                            
##  salmonella bovishield pyramid  titanium  pinkeye   clostrid  colost_source
##  no :87     no :84     no :95   no :111   no : 37   no :111   p :121       
##  yes:58     yes:61     yes:50   yes: 34   yes:108   yes: 34   dp: 24       
##                                                                            
##                                                                            
##                                                                            
##                                                                            
##  colost_past colost_again hosp_milk_pastuerized     action    bull_calves
##  no : 24     no :37       no : 24               screen :124   no :69     
##  yes:121     yes:45       yes:121               premium: 21   yes:76     
##              -99:63                                                      
##                                                                          
##                                                                          
##                                                                          
##  milk_mass_med mass_pen_exp mass_tet_exp mass_rum_exp mass_corr_exp
##  no :79        no :132      no : 29      no : 13      no :76       
##  yes:66        yes: 13      yes:116      yes:132      yes:69       
##                                                                    
##                                                                    
##                                                                    
##                                                                    
##  mass_sul_exp feed_lane_cleaning dust_mgmt     no_classes
##  no :53       scrape:34          no : 24   0        :45  
##  yes:92       lagoon:53          yes:121   1        :56  
##               clean :34                    2 or more:44  
##               sl    :24                                  
##                                                          
##
```

```
# remove variables
pmultall.analyze <- as.data.frame(pmult.all) %>%
                    select(-"gen", -"pen", -"colost_again")

# check
summary(pmultall.analyze)
```

```
##  Farm      season   dan    enr    flr    gam     spc     tild    tilm   
##  A:13   summer:71   S:55   S:55   S:59   S: 41   S:110   S: 36   S: 33  
##  B:24   winter:74   R:90   R:90   R:86   R:104   R: 35   R:109   R:112  
##  C:21                                                                   
##  D:34                                                                   
##  E:24                                                                   
##  F:29                                                                   
##  tul          source_no  onsite_milking resp_vacc first_def salmonella
##  S:120   single    :63   no : 34        no : 24   no :116   no :87    
##  R: 25   low multi :37   yes:111        yes:121   yes: 29   yes:58    
##          high multi:45                                                
##                                                                       
##                                                                       
##                                                                       
##  bovishield pyramid  titanium  pinkeye   clostrid  colost_source colost_past
##  no :84     no :95   no :111   no : 37   no :111   p :121        no : 24    
##  yes:61     yes:50   yes: 34   yes:108   yes: 34   dp: 24        yes:121    
##                                                                             
##                                                                             
##                                                                             
##                                                                             
##  hosp_milk_pastuerized     action    bull_calves milk_mass_med mass_pen_exp
##  no : 24               screen :124   no :69      no :79        no :132     
##  yes:121               premium: 21   yes:76      yes:66        yes: 13     
##                                                                            
##                                                                            
##                                                                            
##                                                                            
##  mass_tet_exp mass_rum_exp mass_corr_exp mass_sul_exp feed_lane_cleaning
##  no : 29      no : 13      no :76        no :53       scrape:34         
##  yes:116      yes:132      yes:69        yes:92       lagoon:53         
##                                                       clean :34         
##                                                       sl    :24         
##                                                                         
##                                                                         
##  dust_mgmt     no_classes
##  no : 24   0        :45  
##  yes:121   1        :56  
##            2 or more:44  
##                          
##                          
##
```

---

Create an allowlist from season to farm to account for clustering of
farm samples in each season. This will be the same allowlist used for
all analyses

```
wl = data.frame(from = c("season"), to = c("Farm"))
```

Create a blocklist of risk factor variables to season or farm, this
accounts for multilevel structure. The blocklist will change for each
analysis depending on which variables are included

```
# create levels
pmult.bl.all = tiers2blacklist(list("season", "Farm",
                     c("dan", "enr", "flr", "gam", "spc", "tild", "tilm", "tul",
                       "source_no", "onsite_milking", "resp_vacc",
                       "first_def", "salmonella", "bovishield", "pyramid",
                       "titanium", "pinkeye", "clostrid",
                       "colost_source", "colost_past", "action",
                       "hosp_milk_pastuerized", "bull_calves", "milk_mass_med",
                       "mass_pen_exp", "mass_tet_exp", "mass_rum_exp", "mass_corr_exp", 
                       "mass_sul_exp", "feed_lane_cleaning", "dust_mgmt", "no_classes")))
pmult.bl.all = rbind(pmult.bl.all, c("season"))
pmult.bl.all
```

```
##       from                    to      
##  [1,] "Farm"                  "season"
##  [2,] "dan"                   "season"
##  [3,] "enr"                   "season"
##  [4,] "flr"                   "season"
##  [5,] "gam"                   "season"
##  [6,] "spc"                   "season"
##  [7,] "tild"                  "season"
##  [8,] "tilm"                  "season"
##  [9,] "tul"                   "season"
## [10,] "source_no"             "season"
## [11,] "onsite_milking"        "season"
## [12,] "resp_vacc"             "season"
## [13,] "first_def"             "season"
## [14,] "salmonella"            "season"
## [15,] "bovishield"            "season"
## [16,] "pyramid"               "season"
## [17,] "titanium"              "season"
## [18,] "pinkeye"               "season"
## [19,] "clostrid"              "season"
## [20,] "colost_source"         "season"
## [21,] "colost_past"           "season"
## [22,] "action"                "season"
## [23,] "hosp_milk_pastuerized" "season"
## [24,] "bull_calves"           "season"
## [25,] "milk_mass_med"         "season"
## [26,] "mass_pen_exp"          "season"
## [27,] "mass_tet_exp"          "season"
## [28,] "mass_rum_exp"          "season"
## [29,] "mass_corr_exp"         "season"
## [30,] "mass_sul_exp"          "season"
## [31,] "feed_lane_cleaning"    "season"
## [32,] "dust_mgmt"             "season"
## [33,] "no_classes"            "season"
## [34,] "dan"                   "Farm"  
## [35,] "enr"                   "Farm"  
## [36,] "flr"                   "Farm"  
## [37,] "gam"                   "Farm"  
## [38,] "spc"                   "Farm"  
## [39,] "tild"                  "Farm"  
## [40,] "tilm"                  "Farm"  
## [41,] "tul"                   "Farm"  
## [42,] "source_no"             "Farm"  
## [43,] "onsite_milking"        "Farm"  
## [44,] "resp_vacc"             "Farm"  
## [45,] "first_def"             "Farm"  
## [46,] "salmonella"            "Farm"  
## [47,] "bovishield"            "Farm"  
## [48,] "pyramid"               "Farm"  
## [49,] "titanium"              "Farm"  
## [50,] "pinkeye"               "Farm"  
## [51,] "clostrid"              "Farm"  
## [52,] "colost_source"         "Farm"  
## [53,] "colost_past"           "Farm"  
## [54,] "action"                "Farm"  
## [55,] "hosp_milk_pastuerized" "Farm"  
## [56,] "bull_calves"           "Farm"  
## [57,] "milk_mass_med"         "Farm"  
## [58,] "mass_pen_exp"          "Farm"  
## [59,] "mass_tet_exp"          "Farm"  
## [60,] "mass_rum_exp"          "Farm"  
## [61,] "mass_corr_exp"         "Farm"  
## [62,] "mass_sul_exp"          "Farm"  
## [63,] "feed_lane_cleaning"    "Farm"  
## [64,] "dust_mgmt"             "Farm"  
## [65,] "no_classes"            "Farm"  
## [66,] "season"                "season"
```

#### 1.2 BNA

Will learn network structure using Hill-Climbing (HC) learning
algorithm and Akaike Information Criterion scoring procedure. The HC
algorithm is a hueristic search that explores DAGs by single-arc
addition and removal. Will test network structure sensitivity to
learning algorithm chosen by also running TABU algorithm and comparing
results.

```
# all farms hc structure learning
pmultfarms.hc <- hc(pmultall.analyze, blacklist = pmult.bl.all, whitelist = wl, score = "aic")

# check score
score(pmultfarms.hc, pmultall.analyze)
```

```
## [1] -1132.98
```

```
# plot customization
pmult.a <- Rgraphviz::layoutGraph(bnlearn::as.graphNEL(pmultfarms.hc))
graph::nodeRenderInfo(pmult.a) <- list(fontsize=60)
Rgraphviz::renderGraph(pmult.a)
```

```
# all farms tabu structure learning
pmultfarms.tabu <- tabu(pmultall.analyze, blacklist = pmult.bl.all, 
                        whitelist = wl, score = "aic")

# check score
score(pmultfarms.tabu, pmultall.analyze)
```

```
## [1] -1132.98
```

```
# plot customization
pmult.b <- Rgraphviz::layoutGraph(bnlearn::as.graphNEL(pmultfarms.tabu))
graph::nodeRenderInfo(pmult.b) <- list(fontsize=60)
Rgraphviz::renderGraph(pmult.b)
```

```
graphviz.compare(pmultfarms.hc, pmultfarms.tabu, shape = "ellipse", 
                 main= c("P.mult Farms HC DAG", "P.mult Farms Tabu DAG"))
```

The DAGs are the same! Indicating the structure is not sensitive to
the learning algorithm chosen.

Now we’ll bootstrap the DAG to identify potentially spurious
associations. This process runs 10,000 bootstrap samples and prunes
edges that do not appear in at least 50% of the structures discovered in
each sample. We’ll again use the HC algorithm and the AIC scoring
procedure.

```
# bootstrap
pmultfarm.boot = boot.strength(pmultall.analyze, R = 10000, algorithm = "hc", 
                        algorithm.args = list(blacklist = pmult.bl.all, 
                                              whitelist = wl, score = "aic"))

# take arcs with strength 50% and return average consensus network
pmultfarm.avg = averaged.network(pmultfarm.boot, threshold = 0.5)

# graph customization
pmult.c <- Rgraphviz::layoutGraph(bnlearn::as.graphNEL(pmultfarm.avg))
graph::nodeRenderInfo(pmult.c) <- list(fontsize=60)
Rgraphviz::renderGraph(pmult.c)
```

Compare the averaged DAG with the single run DAG. The red lines are
the edges that were pruned by bootstrapping.

```
# plot the bootstrapped averaged plot with the original for comparison
graphviz.compare(pmultfarm.avg, pmultfarms.hc, shape = "ellipse", 
                 main= c("Pmult Farm Level Risk Factors Averaged DAG", 
                         "Pmult Farm Level Risk Factors Single DAG"))
```

### Individual level factors

The same analysis above will be run for individual level factors.
Factors under consideration for analysis are individual antibiotic
treatments, what class, how long it has been sense the calf was treated,
total number of antibiotic drugs the calf has received, and when the
last treatment of any antibiotic was.

#### 1.3 Data Wrangling

```
# analytic data frame for individual risk factors
pmult.ind <- pmult.factor %>%
            select("Farm", "season", "brd", "dan", "enr", "flr", "gam", "pen", "spc", 
                   "tet", "tild", "tilm", "tul", "total_abx", "last_treat", 
                   "days_since_pen", "days_since_ceph", "days_since_mac", "days_since_phen",
                   "days_since_tet", "days_since_sul",  "days_since_flr")

# check for missingness
summary(aggr(pmult.ind))
```

```
## 
##  Missings per variable: 
##         Variable Count
##             Farm     0
##           season     0
##              brd     0
##              dan     0
##              enr     0
##              flr     0
##              gam     0
##              pen     0
##              spc     0
##              tet     0
##             tild     0
##             tilm     0
##              tul     0
##        total_abx    24
##       last_treat    24
##   days_since_pen    24
##  days_since_ceph    24
##   days_since_mac    24
##  days_since_phen    24
##   days_since_tet    24
##   days_since_sul    24
##   days_since_flr    24
## 
##  Missings in combinations of variables: 
##                                 Combinations Count  Percent
##  0:0:0:0:0:0:0:0:0:0:0:0:0:0:0:0:0:0:0:0:0:0   121 83.44828
##  0:0:0:0:0:0:0:0:0:0:0:0:0:1:1:1:1:1:1:1:1:1    24 16.55172
```

```
# check cell counts - All Farms
summary(pmult.ind) # -pen, - tet, days_since_pen(<60 days, >=60 days, no treatment), days_since_ceph(<60 days, >=60 days, no treatment), days_since_mac(<60 days, >=60 days, no treatment), days_since_phen(<60 days, >=60 days, no treatment), days_since_tet(<60 days, >=60 days, no treatment), days_since_flr(<60 days, >=60 days, no treatment)
```

```
##  Farm      season    brd     dan    enr    flr    gam     pen     spc    
##  A:13   summer:71   neg:76   S:55   S:55   S:59   S: 41   S:142   S:110  
##  B:24   winter:74   pos:69   R:90   R:90   R:86   R:104   R:  3   R: 35  
##  C:21                                                                    
##  D:34                                                                    
##  E:24                                                                    
##  F:29                                                                    
##  tet     tild    tilm    tul         total_abx       last_treat
##  S:  0   S: 36   S: 33   S:120   0        :21   <16 days  :19  
##  R:145   R:109   R:112   R: 25   1 to 2   :34   16-60 days:33  
##                                  3 or more:66   >=60 days :48  
##                                  NA's     :24   no trx    :21  
##                                                 NA's      :24  
##                                                                
##     days_since_pen   days_since_ceph    days_since_mac   days_since_phen
##  <16 days  : 7     <16 days  :  3    <16 days  : 5     <16 days  : 9    
##  16-60 days:10     16-60 days:  7    16-60 days: 7     16-60 days:18    
##  >=60 days :10     >=60 days : 10    >=60 days :27     >=60 days :42    
##  no trx    :94     no trx    :101    no trx    :82     no trx    :52    
##  NA's      :24     NA's      : 24    NA's      :24     NA's      :24    
##                                                                         
##     days_since_tet   days_since_sul    days_since_flr
##  16-60 days: 2     >=60 days: 20    <16 days  : 2    
##  >=60 days :23     no trx   :101    16-60 days:12    
##  no trx    :96     NA's     : 24    >=60 days :29    
##  NA's      :24                      no trx    :78    
##                                     NA's      :24    
##
```

```
# remove variables
pmultind2 <- as.data.frame(pmult.ind) %>%
                    select(-"pen", -"tet")

# check
summary(pmultind2)
```

```
##  Farm      season    brd     dan    enr    flr    gam     spc     tild   
##  A:13   summer:71   neg:76   S:55   S:55   S:59   S: 41   S:110   S: 36  
##  B:24   winter:74   pos:69   R:90   R:90   R:86   R:104   R: 35   R:109  
##  C:21                                                                    
##  D:34                                                                    
##  E:24                                                                    
##  F:29                                                                    
##  tilm    tul         total_abx       last_treat    days_since_pen
##  S: 33   S:120   0        :21   <16 days  :19   <16 days  : 7    
##  R:112   R: 25   1 to 2   :34   16-60 days:33   16-60 days:10    
##                  3 or more:66   >=60 days :48   >=60 days :10    
##                  NA's     :24   no trx    :21   no trx    :94    
##                                 NA's      :24   NA's      :24    
##                                                                  
##    days_since_ceph    days_since_mac   days_since_phen    days_since_tet
##  <16 days  :  3    <16 days  : 5     <16 days  : 9     16-60 days: 2    
##  16-60 days:  7    16-60 days: 7     16-60 days:18     >=60 days :23    
##  >=60 days : 10    >=60 days :27     >=60 days :42     no trx    :96    
##  no trx    :101    no trx    :82     no trx    :52     NA's      :24    
##  NA's      : 24    NA's      :24     NA's      :24                      
##                                                                         
##    days_since_sul    days_since_flr
##  >=60 days: 20    <16 days  : 2    
##  no trx   :101    16-60 days:12    
##  NA's     : 24    >=60 days :29    
##                   no trx    :78    
##                   NA's      :24    
##
```

```
# recategorize
pmultind2$days_since_pen <- factor(pmult.raw$days_since_pen, order = FALSE,
                                          levels = c(1, 2, 3, 4),
                                          labels = c("<60 days", "<60 days", 
                                                     ">=60 days", "no treatment"))
pmultind2$days_since_ceph <- factor(pmult.raw$days_since_ceph, order = FALSE,
                                          levels = c(1, 2, 3, 4),
                                          labels = c("<60 days", "<60 days", 
                                                     ">=60 days", "no treatment"))
pmultind2$days_since_mac <- factor(pmult.raw$days_since_mac, order = FALSE,
                                          levels = c(1, 2, 3, 4),
                                          labels = c("<60 days", "<60 days", 
                                                     ">=60 days", "no treatment"))
pmultind2$days_since_phen <- factor(pmult.raw$days_since_phen, order = FALSE,
                                          levels = c(1, 2, 3, 4),
                                          labels = c("<60 days", "<60 days", 
                                                     ">=60 days", "no treatment"))
pmultind2$days_since_tet <- factor(pmult.raw$days_since_tet, order = FALSE,
                                          levels = c(1, 2, 3, 4),
                                          labels = c("treatment", "treatment", 
                                                     "treatment", "no treatment"))
pmultind2$days_since_flr <- factor(pmult.raw$days_since_flr, order = FALSE,
                                          levels = c(1, 2, 3, 4),
                                          labels = c("<60 days", "<60 days", 
                                                     ">=60 days", "no treatment"))

# remove Farm E
pmultind2$Farm <- factor(pmult.raw$Farm, order = FALSE,
                            levels = c(1, 2, 3, 4, 6),
                            labels = c("A", "B", "C", "D", "F"))
summary(pmultind2)
```

```
##    Farm       season    brd     dan    enr    flr    gam     spc     tild   
##  A   :13   summer:71   neg:76   S:55   S:55   S:59   S: 41   S:110   S: 36  
##  B   :24   winter:74   pos:69   R:90   R:90   R:86   R:104   R: 35   R:109  
##  C   :21                                                                    
##  D   :34                                                                    
##  F   :29                                                                    
##  NA's:24                                                                    
##  tilm    tul         total_abx       last_treat      days_since_pen
##  S: 33   S:120   0        :21   <16 days  :19   <60 days    :17    
##  R:112   R: 25   1 to 2   :34   16-60 days:33   >=60 days   :10    
##                  3 or more:66   >=60 days :48   no treatment:94    
##                  NA's     :24   no trx    :21   NA's        :24    
##                                 NA's      :24                      
##                                                                    
##      days_since_ceph      days_since_mac     days_since_phen
##  <60 days    : 10    <60 days    :12     <60 days    :27    
##  >=60 days   : 10    >=60 days   :27     >=60 days   :42    
##  no treatment:101    no treatment:82     no treatment:52    
##  NA's        : 24    NA's        :24     NA's        :24    
##                                                             
##                                                             
##       days_since_tet   days_since_sul      days_since_flr
##  treatment   :25     >=60 days: 20    <60 days    :14    
##  no treatment:96     no trx   :101    >=60 days   :29    
##  NA's        :24     NA's     : 24    no treatment:78    
##                                       NA's        :24    
##                                                          
##
```

```
pmultind.analyze <- na.omit(pmultind2)

# check
summary(pmultind.analyze)
```

```
##  Farm      season    brd     dan    enr    flr    gam    spc    tild   tilm  
##  A:13   summer:61   neg:63   S:51   S:51   S:49   S:37   S:88   S:32   S:29  
##  B:24   winter:60   pos:58   R:70   R:70   R:72   R:84   R:33   R:89   R:92  
##  C:21                                                                        
##  D:34                                                                        
##  F:29                                                                        
##  tul        total_abx       last_treat      days_since_pen     days_since_ceph
##  S:98   0        :21   <16 days  :19   <60 days    :17     <60 days    : 10   
##  R:23   1 to 2   :34   16-60 days:33   >=60 days   :10     >=60 days   : 10   
##         3 or more:66   >=60 days :48   no treatment:94     no treatment:101   
##                        no trx    :21                                          
##                                                                               
##       days_since_mac     days_since_phen      days_since_tet   days_since_sul
##  <60 days    :12     <60 days    :27     treatment   :25     >=60 days: 20   
##  >=60 days   :27     >=60 days   :42     no treatment:96     no trx   :101   
##  no treatment:82     no treatment:52                                         
##                                                                              
##                                                                              
##       days_since_flr
##  <60 days    :14    
##  >=60 days   :29    
##  no treatment:78    
##                     
##
```

```
# create levels
pmult.bl.ind = tiers2blacklist(list("season", "Farm",
                     c("brd", "dan", "enr", "flr", "gam", "spc", "tild", "tilm", 
                       "tul", "total_abx", "last_treat", "days_since_pen", 
                       "days_since_ceph", "days_since_mac", "days_since_phen",
                       "days_since_tet", "days_since_sul", "days_since_flr")))
pmult.bl.ind = rbind(pmult.bl.ind, c("season"))
pmult.bl.ind
```

```
##       from              to      
##  [1,] "Farm"            "season"
##  [2,] "brd"             "season"
##  [3,] "dan"             "season"
##  [4,] "enr"             "season"
##  [5,] "flr"             "season"
##  [6,] "gam"             "season"
##  [7,] "spc"             "season"
##  [8,] "tild"            "season"
##  [9,] "tilm"            "season"
## [10,] "tul"             "season"
## [11,] "total_abx"       "season"
## [12,] "last_treat"      "season"
## [13,] "days_since_pen"  "season"
## [14,] "days_since_ceph" "season"
## [15,] "days_since_mac"  "season"
## [16,] "days_since_phen" "season"
## [17,] "days_since_tet"  "season"
## [18,] "days_since_sul"  "season"
## [19,] "days_since_flr"  "season"
## [20,] "brd"             "Farm"  
## [21,] "dan"             "Farm"  
## [22,] "enr"             "Farm"  
## [23,] "flr"             "Farm"  
## [24,] "gam"             "Farm"  
## [25,] "spc"             "Farm"  
## [26,] "tild"            "Farm"  
## [27,] "tilm"            "Farm"  
## [28,] "tul"             "Farm"  
## [29,] "total_abx"       "Farm"  
## [30,] "last_treat"      "Farm"  
## [31,] "days_since_pen"  "Farm"  
## [32,] "days_since_ceph" "Farm"  
## [33,] "days_since_mac"  "Farm"  
## [34,] "days_since_phen" "Farm"  
## [35,] "days_since_tet"  "Farm"  
## [36,] "days_since_sul"  "Farm"  
## [37,] "days_since_flr"  "Farm"  
## [38,] "season"          "season"
```

#### 1.4 BNA

```
# individual factors hc structure learning
pmultind.hc <- hc(pmultind.analyze, blacklist = pmult.bl.ind, 
                  whitelist = wl, score = "aic")

# plot customization
pmult.c <- Rgraphviz::layoutGraph(bnlearn::as.graphNEL(pmultind.hc))
graph::nodeRenderInfo(pmult.c) <- list(fontsize=60)
Rgraphviz::renderGraph(pmult.c)
```

```
# individual factors tabu structure learning
pmultind.tabu <- tabu(pmultind.analyze, blacklist = pmult.bl.ind, 
                      whitelist = wl, 
                      score = "aic")

# plot customization
pmult.d <- Rgraphviz::layoutGraph(bnlearn::as.graphNEL(pmultind.tabu))
graph::nodeRenderInfo(pmult.d) <- list(fontsize=60)
Rgraphviz::renderGraph(pmult.d)
```

```
graphviz.compare(pmultind.hc, pmultind.tabu, shape = "ellipse", 
                 main= c("Pmult Individual Risk Factors HC DAG", 
                         "Pmult Individual Risk Factors Tabu DAG"))
```

```
# bootstrap
pmultind.boot = boot.strength(pmultind.analyze, R = 10000, algorithm = "hc", 
                        algorithm.args = list(blacklist = pmult.bl.ind, 
                                              whitelist = wl, score = "aic"))

# take arcs with strength of 50% and return average consensus network
pmultind.avg = averaged.network(pmultind.boot, threshold = 0.5)

# graph customization
pmult.e <- Rgraphviz::layoutGraph(bnlearn::as.graphNEL(pmultind.avg))
graph::nodeRenderInfo(pmult.e) <- list(fontsize=60)
Rgraphviz::renderGraph(pmult.e)
```

```
# plot the bootstrapped averaged plot with the original for comparison
graphviz.compare(pmultind.avg, pmultind.hc, shape = "ellipse", 
                 main= c("Pmult Individual Level Risk Factors Averaged DAG", 
                         "Pmult Individual Level Risk Factors Single DAG"))
```

#### 1.5 Parameter Learning

Farm level query

```
# fit the parameters - bayesian method
pcmult.bayes = bn.fit(pmultfarm.avg, pmultall.analyze, method = "bayes")

# probability table
pcmult.bayes
```

```
## 
##   Bayesian network parameters
## 
##   Parameters of node Farm (multinomial distribution)
## 
## Conditional probability table:
##  
##     season
## Farm     summer     winter
##    A 0.08508159 0.09507830
##    B 0.25291375 0.08165548
##    C 0.15501166 0.13534676
##    D 0.22494172 0.24272931
##    E 0.14102564 0.18903803
##    F 0.14102564 0.25615213
## 
##   Parameters of node season (multinomial distribution)
## 
## Conditional probability table:
##    summer   winter 
## 0.489726 0.510274 
## 
##   Parameters of node dan (multinomial distribution)
## 
## Conditional probability table:
##  
##    tild
## dan          S          R
##   S 0.93835616 0.19406393
##   R 0.06164384 0.80593607
## 
##   Parameters of node enr (multinomial distribution)
## 
## Conditional probability table:
##  
##    dan
## enr           S           R
##   S 0.995495495 0.002762431
##   R 0.004504505 0.997237569
## 
##   Parameters of node flr (multinomial distribution)
## 
## Conditional probability table:
##          S         R 
## 0.4075342 0.5924658 
## 
##   Parameters of node gam (multinomial distribution)
## 
## Conditional probability table:
##  
##    Farm
## gam          A          B          C          D          E          F
##   S 0.38607595 0.08620690 0.05118110 0.82195122 0.16896552 0.03714286
##   R 0.61392405 0.91379310 0.94881890 0.17804878 0.83103448 0.96285714
## 
##   Parameters of node spc (multinomial distribution)
## 
## Conditional probability table:
##  
##    dan
## spc         S         R
##   S 0.4189189 0.9640884
##   R 0.5810811 0.0359116
## 
##   Parameters of node tild (multinomial distribution)
## 
## Conditional probability table:
##  
## , , onsite_milking = no
## 
##     gam
## tild           S           R
##    S 0.180000000 0.004424779
##    R 0.820000000 0.995575221
## 
## , , onsite_milking = yes
## 
##     gam
## tild           S           R
##    S 0.996453901 0.001639344
##    R 0.003546099 0.998360656
## 
## 
##   Parameters of node tilm (multinomial distribution)
## 
## Conditional probability table:
##  
##     tild
## tilm           S           R
##    S 0.910958904 0.002283105
##    R 0.089041096 0.997716895
## 
##   Parameters of node tul (multinomial distribution)
## 
## Conditional probability table:
##  
##    spc
## tul         S         R
##   S 0.8891403 0.6267606
##   R 0.1108597 0.3732394
## 
##   Parameters of node source_no (multinomial distribution)
## 
## Conditional probability table:
##  
##             Farm
## source_no              A           B           C           D           E
##   single     0.004219409 0.002298851 0.002624672 0.996747967 0.002298851
##   low multi  0.991561181 0.995402299 0.002624672 0.001626016 0.002298851
##   high multi 0.004219409 0.002298851 0.994750656 0.001626016 0.995402299
##             Farm
## source_no              F
##   single     0.996190476
##   low multi  0.001904762
##   high multi 0.001904762
## 
##   Parameters of node onsite_milking (multinomial distribution)
## 
## Conditional probability table:
##  
##               feed_lane_cleaning
## onsite_milking      scrape      lagoon       clean          sl
##            no  0.996350365 0.002347418 0.003649635 0.005154639
##            yes 0.003649635 0.997652582 0.996350365 0.994845361
## 
##   Parameters of node resp_vacc (multinomial distribution)
## 
## Conditional probability table:
##  
##          feed_lane_cleaning
## resp_vacc      scrape      lagoon       clean          sl
##       no  0.003649635 0.002347418 0.003649635 0.994845361
##       yes 0.996350365 0.997652582 0.996350365 0.005154639
## 
##   Parameters of node first_def (multinomial distribution)
## 
## Conditional probability table:
##  
##          Farm
## first_def           A           B           C           D           E
##       no  0.993670886 0.996551724 0.996062992 0.997560976 0.996551724
##       yes 0.006329114 0.003448276 0.003937008 0.002439024 0.003448276
##          Farm
## first_def           F
##       no  0.002857143
##       yes 0.997142857
## 
##   Parameters of node salmonella (multinomial distribution)
## 
## Conditional probability table:
##  
##           Farm
## salmonella           A           B           C           D           E
##        no  0.006329114 0.003448276 0.003937008 0.997560976 0.996551724
##        yes 0.993670886 0.996551724 0.996062992 0.002439024 0.003448276
##           Farm
## salmonella           F
##        no  0.997142857
##        yes 0.002857143
## 
##   Parameters of node bovishield (multinomial distribution)
## 
## Conditional probability table:
##  
##           Farm
## bovishield           A           B           C           D           E
##        no  0.006329114 0.003448276 0.996062992 0.997560976 0.003448276
##        yes 0.993670886 0.996551724 0.003937008 0.002439024 0.996551724
##           Farm
## bovishield           F
##        no  0.997142857
##        yes 0.002857143
## 
##   Parameters of node pyramid (multinomial distribution)
## 
## Conditional probability table:
##  
##        Farm
## pyramid           A           B           C           D           E           F
##     no  0.993670886 0.996551724 0.003937008 0.997560976 0.996551724 0.002857143
##     yes 0.006329114 0.003448276 0.996062992 0.002439024 0.003448276 0.997142857
## 
##   Parameters of node titanium (multinomial distribution)
## 
## Conditional probability table:
##  
##         feed_lane_cleaning
## titanium      scrape      lagoon       clean          sl
##      no  0.996350365 0.997652582 0.003649635 0.994845361
##      yes 0.003649635 0.002347418 0.996350365 0.005154639
## 
##   Parameters of node pinkeye (multinomial distribution)
## 
## Conditional probability table:
##  
##        Farm
## pinkeye           A           B           C           D           E           F
##     no  0.993670886 0.003448276 0.003937008 0.002439024 0.996551724 0.002857143
##     yes 0.006329114 0.996551724 0.996062992 0.997560976 0.003448276 0.997142857
## 
##   Parameters of node clostrid (multinomial distribution)
## 
## Conditional probability table:
##  
##         titanium
## clostrid          no         yes
##      no  0.997757848 0.007246377
##      yes 0.002242152 0.992753623
## 
##   Parameters of node colost_source (multinomial distribution)
## 
## Conditional probability table:
##  
##              resp_vacc
## colost_source          no         yes
##            p  0.010204082 0.997942387
##            dp 0.989795918 0.002057613
## 
##   Parameters of node colost_past (multinomial distribution)
## 
## Conditional probability table:
##  
##            resp_vacc
## colost_past          no         yes
##         no  0.989795918 0.002057613
##         yes 0.010204082 0.997942387
## 
##   Parameters of node hosp_milk_pastuerized (multinomial distribution)
## 
## Conditional probability table:
##  
##                      resp_vacc
## hosp_milk_pastuerized          no         yes
##                   no  0.989795918 0.002057613
##                   yes 0.010204082 0.997942387
## 
##   Parameters of node action (multinomial distribution)
## 
## Conditional probability table:
##  
##          Farm
## action              A           B           C           D           E
##   screen  0.993670886 0.996551724 0.003937008 0.997560976 0.996551724
##   premium 0.006329114 0.003448276 0.996062992 0.002439024 0.003448276
##          Farm
## action              F
##   screen  0.997142857
##   premium 0.002857143
## 
##   Parameters of node bull_calves (multinomial distribution)
## 
## Conditional probability table:
##  
##            Farm
## bull_calves           A           B           C           D           E
##         no  0.006329114 0.996551724 0.996062992 0.002439024 0.996551724
##         yes 0.993670886 0.003448276 0.003937008 0.997560976 0.003448276
##            Farm
## bull_calves           F
##         no  0.002857143
##         yes 0.997142857
## 
##   Parameters of node milk_mass_med (multinomial distribution)
## 
## Conditional probability table:
##  
##              Farm
## milk_mass_med           A           B           C           D           E
##           no  0.006329114 0.996551724 0.996062992 0.997560976 0.003448276
##           yes 0.993670886 0.003448276 0.003937008 0.002439024 0.996551724
##              Farm
## milk_mass_med           F
##           no  0.002857143
##           yes 0.997142857
## 
##   Parameters of node mass_pen_exp (multinomial distribution)
## 
## Conditional probability table:
##  
##             Farm
## mass_pen_exp           A           B           C           D           E
##          no  0.006329114 0.996551724 0.996062992 0.997560976 0.996551724
##          yes 0.993670886 0.003448276 0.003937008 0.002439024 0.003448276
##             Farm
## mass_pen_exp           F
##          no  0.997142857
##          yes 0.002857143
## 
##   Parameters of node mass_tet_exp (multinomial distribution)
## 
## Conditional probability table:
##  
##             first_def
## mass_tet_exp          no         yes
##          no  0.002145923 0.991525424
##          yes 0.997854077 0.008474576
## 
##   Parameters of node mass_rum_exp (multinomial distribution)
## 
## Conditional probability table:
##  
##             mass_pen_exp
## mass_rum_exp          no         yes
##          no  0.001886792 0.981481481
##          yes 0.998113208 0.018518519
## 
##   Parameters of node mass_corr_exp (multinomial distribution)
## 
## Conditional probability table:
##  
##              bull_calves
## mass_corr_exp          no         yes
##           no  0.003597122 0.996732026
##           yes 0.996402878 0.003267974
## 
##   Parameters of node mass_sul_exp (multinomial distribution)
## 
## Conditional probability table:
##  
##             Farm
## mass_sul_exp           A           B           C           D           E
##          no  0.006329114 0.003448276 0.003937008 0.002439024 0.996551724
##          yes 0.993670886 0.996551724 0.996062992 0.997560976 0.003448276
##             Farm
## mass_sul_exp           F
##          no  0.997142857
##          yes 0.002857143
## 
##   Parameters of node feed_lane_cleaning (multinomial distribution)
## 
## Conditional probability table:
##  
##                   Farm
## feed_lane_cleaning           A           B           C           D           E
##             scrape 0.990506329 0.001724138 0.994094488 0.001219512 0.001724138
##             lagoon 0.003164557 0.994827586 0.001968504 0.001219512 0.001724138
##             clean  0.003164557 0.001724138 0.001968504 0.996341463 0.001724138
##             sl     0.003164557 0.001724138 0.001968504 0.001219512 0.994827586
##                   Farm
## feed_lane_cleaning           F
##             scrape 0.001428571
##             lagoon 0.995714286
##             clean  0.001428571
##             sl     0.001428571
## 
##   Parameters of node dust_mgmt (multinomial distribution)
## 
## Conditional probability table:
##  
##          resp_vacc
## dust_mgmt          no         yes
##       no  0.989795918 0.002057613
##       yes 0.010204082 0.997942387
## 
##   Parameters of node no_classes (multinomial distribution)
## 
## Conditional probability table:
##  
##            Farm
## no_classes            A           B           C           D           E
##   0         0.763713080 0.126436782 0.002624672 0.030894309 0.995402299
##   1         0.232067511 0.374712644 0.664041995 0.382113821 0.002298851
##   2 or more 0.004219409 0.498850575 0.333333333 0.586991870 0.002298851
##            Farm
## no_classes            F
##   0         0.241904762
##   1         0.584761905
##   2 or more 0.173333333
```

```
# conditional probabilities of AMD
pcmult.bayes$gam
```

```
## 
##   Parameters of node gam (multinomial distribution)
## 
## Conditional probability table:
##  
##    Farm
## gam          A          B          C          D          E          F
##   S 0.38607595 0.08620690 0.05118110 0.82195122 0.16896552 0.03714286
##   R 0.61392405 0.91379310 0.94881890 0.17804878 0.83103448 0.96285714
```

```
pcmult.bayes$tild
```

```
## 
##   Parameters of node tild (multinomial distribution)
## 
## Conditional probability table:
##  
## , , onsite_milking = no
## 
##     gam
## tild           S           R
##    S 0.180000000 0.004424779
##    R 0.820000000 0.995575221
## 
## , , onsite_milking = yes
## 
##     gam
## tild           S           R
##    S 0.996453901 0.001639344
##    R 0.003546099 0.998360656
```

```
pcmult.bayes$tilm
```

```
## 
##   Parameters of node tilm (multinomial distribution)
## 
## Conditional probability table:
##  
##     tild
## tilm           S           R
##    S 0.910958904 0.002283105
##    R 0.089041096 0.997716895
```

```
pcmult.bayes$dan
```

```
## 
##   Parameters of node dan (multinomial distribution)
## 
## Conditional probability table:
##  
##    tild
## dan          S          R
##   S 0.93835616 0.19406393
##   R 0.06164384 0.80593607
```

```
pcmult.bayes$enr
```

```
## 
##   Parameters of node enr (multinomial distribution)
## 
## Conditional probability table:
##  
##    dan
## enr           S           R
##   S 0.995495495 0.002762431
##   R 0.004504505 0.997237569
```

```
pcmult.bayes$spc
```

```
## 
##   Parameters of node spc (multinomial distribution)
## 
## Conditional probability table:
##  
##    dan
## spc         S         R
##   S 0.4189189 0.9640884
##   R 0.5810811 0.0359116
```

```
set.seed(111)
cpquery(pcmult.bayes, (gam =="R"), tild == "R")
```

```
## [1] 0.9484033
```

```
cpquery(pcmult.bayes, (gam =="R"), tild == "S")
```

```
## [1] 0.008209539
```

```
cpquery(pcmult.bayes, (tild =="R"), gam == "R")
```

```
## [1] 0.9976157
```

```
cpquery(pcmult.bayes, (tild =="R"), gam == "S")
```

```
## [1] 0.1315699
```

```
set.seed(112)
cpquery(pcmult.bayes, (tild == "R"), onsite_milking == "yes")
```

```
## [1] 0.6855354
```

```
cpquery(pcmult.bayes, (tild == "R"), onsite_milking == "no")
```

```
## [1] 0.9600666
```

```
cpquery(pcmult.bayes, (tild == "S"), onsite_milking == "yes")
```

```
## [1] 0.3161341
```

```
cpquery(pcmult.bayes, (tild == "S"), onsite_milking == "no")
```

```
## [1] 0.03434951
```

```
set.seed(113)
cpquery(pcmult.bayes, (tild == "R"), onsite_milking == "yes" & feed_lane_cleaning == "scrape")
```

```
## [1] 0.75
```

```
cpquery(pcmult.bayes, (tild == "R"), onsite_milking == "no" & feed_lane_cleaning == "scrape")
```

```
## [1] 0.9666244
```

```
cpquery(pcmult.bayes, (tild == "S"), onsite_milking == "yes" & feed_lane_cleaning == "scrape")
```

```
## [1] 0.3636364
```

```
cpquery(pcmult.bayes, (tild == "S"), onsite_milking == "no" & feed_lane_cleaning == "scrape")
```

```
## [1] 0.0356832
```

```
set.seed(114)
cpquery(pcmult.bayes, (tild == "R"), onsite_milking == "yes" & feed_lane_cleaning == "lagoon")
```

```
## [1] 0.9413061
```

```
cpquery(pcmult.bayes, (tild == "R"), onsite_milking == "no" & feed_lane_cleaning == "lagoon")
```

```
## [1] 0.875
```

```
cpquery(pcmult.bayes, (tild == "S"), onsite_milking == "yes" & feed_lane_cleaning == "lagoon")
```

```
## [1] 0.05970967
```

```
cpquery(pcmult.bayes, (tild == "S"), onsite_milking == "no" & feed_lane_cleaning == "lagoon")
```

```
## [1] 0.125
```

```
set.seed(115)
cpquery(pcmult.bayes, (tild == "R"), onsite_milking == "yes" & feed_lane_cleaning == "clean")
```

```
## [1] 0.1745962
```

```
cpquery(pcmult.bayes, (tild == "R"), onsite_milking == "no" & feed_lane_cleaning == "clean")
```

```
## [1] 0.7142857
```

```
cpquery(pcmult.bayes, (tild == "S"), onsite_milking == "yes" & feed_lane_cleaning == "clean")
```

```
## [1] 0.8199748
```

```
cpquery(pcmult.bayes, (tild == "S"), onsite_milking == "no" & feed_lane_cleaning == "clean")
```

```
## [1] 0.1666667
```

Individual level query

```
# fit the parameters - bayesian method
pc.bayes = bn.fit(pmultind.avg, pmultind.analyze, method = "bayes")

# probability table
pc.bayes
```

```
## 
##   Bayesian network parameters
## 
##   Parameters of node Farm (multinomial distribution)
## 
## Conditional probability table:
##  
##     season
## Farm     summer     winter
##    A 0.09918699 0.11735537
##    B 0.29430894 0.10082645
##    C 0.18048780 0.16694215
##    D 0.26178862 0.29917355
##    F 0.16422764 0.31570248
## 
##   Parameters of node season (multinomial distribution)
## 
## Conditional probability table:
##     summer    winter 
## 0.5040984 0.4959016 
## 
##   Parameters of node brd (multinomial distribution)
## 
## Conditional probability table:
##        neg       pos 
## 0.5204918 0.4795082 
## 
##   Parameters of node dan (multinomial distribution)
## 
## Conditional probability table:
##  
## , , tild = S
## 
##    Farm
## dan           A           B           C           D           F
##   S 0.954545455 0.023809524 0.500000000 0.998220641 0.954545455
##   R 0.045454545 0.976190476 0.500000000 0.001779359 0.045454545
## 
## , , tild = R
## 
##    Farm
## dan           A           B           C           D           F
##   S 0.004132231 0.047511312 0.428909953 0.008196721 0.393238434
##   R 0.995867769 0.952488688 0.571090047 0.991803279 0.606761566
## 
## 
##   Parameters of node enr (multinomial distribution)
## 
## Conditional probability table:
##  
##    dan
## enr           S           R
##   S 0.995145631 0.003546099
##   R 0.004854369 0.996453901
## 
##   Parameters of node flr (multinomial distribution)
## 
## Conditional probability table:
##  
## , , season = summer
## 
##    Farm
## flr           A           B           C           D           F
##   S 0.008196721 0.058011050 0.274774775 0.934782609 0.103960396
##   R 0.991803279 0.941988950 0.725225225 0.065217391 0.896039604
## 
## , , season = winter
## 
##    Farm
## flr           A           B           C           D           F
##   S 0.147887324 0.500000000 0.103960396 0.997237569 0.316753927
##   R 0.852112676 0.500000000 0.896039604 0.002762431 0.683246073
## 
## 
##   Parameters of node gam (multinomial distribution)
## 
## Conditional probability table:
##  
##    Farm
## gam          A          B          C          D          F
##   S 0.38636364 0.08677686 0.05188679 0.82163743 0.03767123
##   R 0.61363636 0.91322314 0.94811321 0.17836257 0.96232877
## 
##   Parameters of node spc (multinomial distribution)
## 
## Conditional probability table:
##  
## , , dan = S
## 
##    Farm
## spc           A           B           C           D           F
##   S 0.954545455 0.045454545 0.005494505 0.286476868 0.995867769
##   R 0.045454545 0.954545455 0.994505495 0.713523132 0.004132231
## 
## , , dan = R
## 
##    Farm
## spc           A           B           C           D           F
##   S 0.995867769 0.954545455 0.830578512 0.991803279 0.997076023
##   R 0.004132231 0.045454545 0.169421488 0.008196721 0.002923977
## 
## 
##   Parameters of node tild (multinomial distribution)
## 
## Conditional probability table:
##  
##     gam
## tild          S          R
##    S 0.86000000 0.00295858
##    R 0.14000000 0.99704142
## 
##   Parameters of node tilm (multinomial distribution)
## 
## Conditional probability table:
##  
##     tild
## tilm           S           R
##    S 0.900000000 0.002793296
##    R 0.100000000 0.997206704
## 
##   Parameters of node tul (multinomial distribution)
## 
## Conditional probability table:
##  
## , , spc = S
## 
##    Farm
## tul           A           B           C           D           F
##   S 0.690839695 0.771493213 0.995049505 0.996453901 0.963917526
##   R 0.309160305 0.228506787 0.004950495 0.003546099 0.036082474
## 
## , , spc = R
## 
##    Farm
## tul           A           B           C           D           F
##   S 0.500000000 0.023809524 0.004504505 0.997512438 0.500000000
##   R 0.500000000 0.976190476 0.995495495 0.002487562 0.500000000
## 
## 
##   Parameters of node total_abx (multinomial distribution)
## 
## Conditional probability table:
##  
##            Farm
## total_abx             A           B           C           D           F
##   0         0.762626263 0.126721763 0.003144654 0.031189084 0.242009132
##   1 to 2    0.156565657 0.168044077 0.380503145 0.352826511 0.276255708
##   3 or more 0.080808081 0.705234160 0.616352201 0.615984405 0.481735160
## 
##   Parameters of node last_treat (multinomial distribution)
## 
## Conditional probability table:
##  
##             total_abx
## last_treat             0      1 to 2   3 or more
##   <16 days   0.003906250 0.206310680 0.182160804
##   16-60 days 0.003906250 0.264563107 0.363065327
##   >=60 days  0.003906250 0.526699029 0.453517588
##   no trx     0.988281250 0.002427184 0.001256281
## 
##   Parameters of node days_since_pen (multinomial distribution)
## 
## Conditional probability table:
##  
##               Farm
## days_since_pen           A           B           C           D           F
##   <60 days     0.005050505 0.126721763 0.663522013 0.001949318 0.002283105
##   >=60 days    0.005050505 0.374655647 0.050314465 0.001949318 0.002283105
##   no treatment 0.989898990 0.498622590 0.286163522 0.996101365 0.995433790
## 
##   Parameters of node days_since_ceph (multinomial distribution)
## 
## Conditional probability table:
##      <60 days    >=60 days no treatment 
##   0.08469945   0.08469945   0.83060109 
## 
##   Parameters of node days_since_mac (multinomial distribution)
## 
## Conditional probability table:
##  
##               last_treat
## days_since_mac    <16 days  16-60 days   >=60 days      no trx
##   <60 days     0.264069264 0.213032581 0.001727116 0.003921569
##   >=60 days    0.160173160 0.243107769 0.333333333 0.003921569
##   no treatment 0.575757576 0.543859649 0.664939551 0.992156863
## 
##   Parameters of node days_since_phen (multinomial distribution)
## 
## Conditional probability table:
##  
## , , last_treat = <16 days
## 
##                season
## days_since_phen      summer      winter
##    <60 days     0.881278539 0.497942387
##    >=60 days    0.004566210 0.300411523
##    no treatment 0.114155251 0.201646091
## 
## , , last_treat = 16-60 days
## 
##                season
## days_since_phen      summer      winter
##    <60 days     0.644768856 0.188630491
##    >=60 days    0.060827251 0.560723514
##    no treatment 0.294403893 0.250645995
## 
## , , last_treat = >=60 days
## 
##                season
## days_since_phen      summer      winter
##    <60 days     0.001801802 0.001658375
##    >=60 days    0.563963964 0.638474295
##    no treatment 0.434234234 0.359867330
## 
## , , last_treat = no trx
## 
##                season
## days_since_phen      summer      winter
##    <60 days     0.003436426 0.004566210
##    >=60 days    0.003436426 0.004566210
##    no treatment 0.993127148 0.990867580
## 
## 
##   Parameters of node days_since_tet (multinomial distribution)
## 
## Conditional probability table:
##  
##               Farm
## days_since_tet           A           B           C           D           F
##   treatment    0.007575758 0.004132231 0.004716981 0.587719298 0.174657534
##   no treatment 0.992424242 0.995867769 0.995283019 0.412280702 0.825342466
## 
##   Parameters of node days_since_sul (multinomial distribution)
## 
## Conditional probability table:
##  
##               Farm
## days_since_sul           A           B           C           D           F
##      >=60 days 0.007575758 0.004132231 0.004716981 0.587719298 0.003424658
##      no trx    0.992424242 0.995867769 0.995283019 0.412280702 0.996575342
## 
##   Parameters of node days_since_flr (multinomial distribution)
## 
## Conditional probability table:
##      <60 days    >=60 days no treatment 
##    0.1174863    0.2404372    0.6420765
```

```
pc.bayes$flr
```

```
## 
##   Parameters of node flr (multinomial distribution)
## 
## Conditional probability table:
##  
## , , season = summer
## 
##    Farm
## flr           A           B           C           D           F
##   S 0.008196721 0.058011050 0.274774775 0.934782609 0.103960396
##   R 0.991803279 0.941988950 0.725225225 0.065217391 0.896039604
## 
## , , season = winter
## 
##    Farm
## flr           A           B           C           D           F
##   S 0.147887324 0.500000000 0.103960396 0.997237569 0.316753927
##   R 0.852112676 0.500000000 0.896039604 0.002762431 0.683246073
```

```
pc.bayes$gam
```

```
## 
##   Parameters of node gam (multinomial distribution)
## 
## Conditional probability table:
##  
##    Farm
## gam          A          B          C          D          F
##   S 0.38636364 0.08677686 0.05188679 0.82163743 0.03767123
##   R 0.61363636 0.91322314 0.94811321 0.17836257 0.96232877
```

```
pc.bayes$tild
```

```
## 
##   Parameters of node tild (multinomial distribution)
## 
## Conditional probability table:
##  
##     gam
## tild          S          R
##    S 0.86000000 0.00295858
##    R 0.14000000 0.99704142
```

```
pc.bayes$tilm
```

```
## 
##   Parameters of node tilm (multinomial distribution)
## 
## Conditional probability table:
##  
##     tild
## tilm           S           R
##    S 0.900000000 0.002793296
##    R 0.100000000 0.997206704
```

```
pc.bayes$dan
```

```
## 
##   Parameters of node dan (multinomial distribution)
## 
## Conditional probability table:
##  
## , , tild = S
## 
##    Farm
## dan           A           B           C           D           F
##   S 0.954545455 0.023809524 0.500000000 0.998220641 0.954545455
##   R 0.045454545 0.976190476 0.500000000 0.001779359 0.045454545
## 
## , , tild = R
## 
##    Farm
## dan           A           B           C           D           F
##   S 0.004132231 0.047511312 0.428909953 0.008196721 0.393238434
##   R 0.995867769 0.952488688 0.571090047 0.991803279 0.606761566
```

```
pc.bayes$enr
```

```
## 
##   Parameters of node enr (multinomial distribution)
## 
## Conditional probability table:
##  
##    dan
## enr           S           R
##   S 0.995145631 0.003546099
##   R 0.004854369 0.996453901
```

```
pc.bayes$spc
```

```
## 
##   Parameters of node spc (multinomial distribution)
## 
## Conditional probability table:
##  
## , , dan = S
## 
##    Farm
## spc           A           B           C           D           F
##   S 0.954545455 0.045454545 0.005494505 0.286476868 0.995867769
##   R 0.045454545 0.954545455 0.994505495 0.713523132 0.004132231
## 
## , , dan = R
## 
##    Farm
## spc           A           B           C           D           F
##   S 0.995867769 0.954545455 0.830578512 0.991803279 0.997076023
##   R 0.004132231 0.045454545 0.169421488 0.008196721 0.002923977
```

```
pc.bayes$tul
```

```
## 
##   Parameters of node tul (multinomial distribution)
## 
## Conditional probability table:
##  
## , , spc = S
## 
##    Farm
## tul           A           B           C           D           F
##   S 0.690839695 0.771493213 0.995049505 0.996453901 0.963917526
##   R 0.309160305 0.228506787 0.004950495 0.003546099 0.036082474
## 
## , , spc = R
## 
##    Farm
## tul           A           B           C           D           F
##   S 0.500000000 0.023809524 0.004504505 0.997512438 0.500000000
##   R 0.500000000 0.976190476 0.995495495 0.002487562 0.500000000
```

```
set.seed(115)
cpquery(pc.bayes, (tul =="R"), spc == "R")
```

```
## [1] 0.4290794
```

```
cpquery(pc.bayes, (tul =="R"), spc == "S")
```

```
## [1] 0.1120944
```

```
cpquery(pc.bayes, (tul =="S"), spc == "R")
```

```
## [1] 0.5466461
```

```
cpquery(pc.bayes, (tul =="S"), spc == "S")
```

```
## [1] 0.8861281
```

```
set.seed(116)
cpquery(pc.bayes, (flr =="R"), season == "summer")
```

```
## [1] 0.6734533
```

```
cpquery(pc.bayes, (flr =="R"), season == "winter")
```

```
## [1] 0.5231815
```

```
cpquery(pc.bayes, (flr =="S"), season == "summer")
```

```
## [1] 0.3252704
```

```
cpquery(pc.bayes, (flr =="S"), season == "winter")
```

```
## [1] 0.4759808
```

# *2. M. haemolytica*

The same analysis above will be run for the remaining two respiratory
pathogens.

### Farm level factors

#### 2.1 Data Wrangling

```
# import data
mhaem.df <- read_csv("MHAEM.csv", show_col_types = FALSE)
# glimpse(mhaem.df)
```

```
# connect calf treatment variables to mhaem df
mhaem.df <- mhaem.df %>%
  mutate(calf_ID = ID) %>%
  select(-"ID")

mhaem.raw <- left_join(mhaem.df, calf.trx, by = "calf_ID")

# make variables factors, add labels
mhaem.factor = mhaem.raw
mhaem.factor$season <- factor(mhaem.raw$season, order = FALSE,
                              levels = c(1, 2),
                              labels = c("summer", "winter"))
mhaem.factor$brd <- factor(mhaem.raw$brd, order = FALSE,
                           levels = c(0, 1),
                           labels = c("neg", "pos"))
mhaem.factor$cef <- factor(mhaem.raw$cef, order = FALSE,
                           levels = c(0, 1),
                           labels = c("S", "R"))
mhaem.factor$dan <- factor(mhaem.raw$dan, order = FALSE,
                           levels = c(0, 1),
                           labels = c("S", "R"))
mhaem.factor$enr <- factor(mhaem.raw$enr, order = FALSE,
                           levels = c(0, 1),
                           labels = c("S", "R"))
mhaem.factor$flr <- factor(mhaem.raw$flr, order = FALSE,
                         levels = c(0, 1),
                         labels = c("S", "R"))
mhaem.factor$gam <- factor(mhaem.raw$gam, order = FALSE,
                           levels = c(0, 1),
                           labels = c("S", "R"))
mhaem.factor$gen <- factor(mhaem.raw$gen, order = FALSE,
                           levels = c(0, 1),
                           labels = c("S", "R"))
mhaem.factor$pen <- factor(mhaem.raw$pen, order = FALSE,
                           levels = c(0, 1),
                           labels = c("S", "R"))
mhaem.factor$spc <- factor(mhaem.raw$spc, order = FALSE,
                           levels = c(0, 1),
                           labels = c("S", "R"))
mhaem.factor$tet <- factor(mhaem.raw$tet, order = FALSE,
                           levels = c(0, 1),
                           labels = c("S", "R"))
mhaem.factor$tild <- factor(mhaem.raw$tild, order = FALSE,
                            levels = c(0, 1),
                            labels = c("S", "R"))
mhaem.factor$tilm <- factor(mhaem.raw$tilm, order = FALSE,
                            levels = c(0, 1),
                            labels = c("S", "R"))
mhaem.factor$tul <- factor(mhaem.raw$tul, order = FALSE,
                           levels = c(0, 1),
                           labels = c("S", "R"))
mhaem.factor$source_no <- factor(mhaem.raw$source_no, order = FALSE,
                                 levels = c(1, 2, 3),
                                 labels = c("single", "low multi", "high multi"))
mhaem.factor$onsite_milking <- factor(mhaem.raw$onsite_milking, order = FALSE,
                                      levels = c(0, 1),
                                      labels = c("no", "yes"))
mhaem.factor$resp_vacc <- factor(mhaem.raw$resp_vacc, order = FALSE,
                                 levels = c(0, 1),
                                 labels = c("no", "yes"))
mhaem.factor$first_def <- factor(mhaem.raw$first_def, order = FALSE,
                                 levels = c(0, 1),
                                 labels = c("no", "yes"))
mhaem.factor$salmonella <- factor(mhaem.raw$salmonella, order = FALSE,
                           levels = c(0, 1),
                           labels = c("no", "yes"))
mhaem.factor$bovishield <- factor(mhaem.raw$bovishield, order = FALSE,
                                  levels = c(0, 1),
                                  labels = c("no", "yes"))
mhaem.factor$pyramid <- factor(mhaem.raw$pyramid, order = FALSE,
                               levels = c(0, 1),
                               labels = c("no", "yes"))
mhaem.factor$titanium <- factor(mhaem.raw$titanium, order = FALSE,
                                levels = c(0, 1),
                                labels = c("no", "yes"))
mhaem.factor$pinkeye <- factor(mhaem.raw$pinkeye, order = FALSE,
                               levels = c(0, 1),
                               labels = c("no", "yes"))
mhaem.factor$clostrid <- factor(mhaem.raw$clostrid, order = FALSE,
                                levels = c(0, 1),
                                labels = c("no", "yes"))
mhaem.factor$colost_source <- factor(mhaem.raw$colost_source, order = FALSE,
                                     levels = c(1, 2),
                                     labels = c("p", "dp"))
mhaem.factor$colost_past <- factor(mhaem.raw$colost_past, order = FALSE,
                                   levels = c(0, 1),
                                   labels = c("no", "yes"))
mhaem.factor$colost_again <- factor(mhaem.raw$colost_again, order = FALSE,
                                    levels = c(0, 1, 99),
                                    labels = c("no", "yes", "-99"))
mhaem.factor$hosp_milk_pastuerized <- factor(mhaem.raw$hosp_milk_pastuerized, 
                                             order = FALSE,
                                             levels = c(0, 1),
                                             labels = c("no", "yes"))
mhaem.factor$action <- factor(mhaem.raw$action, order = FALSE,
                              levels = c(1, 2),
                              labels = c("screen", "premium"))
mhaem.factor$bull_calves <- factor(mhaem.raw$bull_calves, order = FALSE,
                                   levels = c(0, 1),
                                   labels = c("no", "yes"))
mhaem.factor$milk_mass_med <- factor(mhaem.raw$milk_mass_med, order = FALSE,
                                     levels = c(0, 1),
                                     labels = c("no", "yes"))
mhaem.factor$mass_pen_exp <- factor(mhaem.raw$mass_pen_exp, order = FALSE,
                                    levels = c(0, 1),
                                    labels = c("no", "yes"))
mhaem.factor$mass_tet_exp <- factor(mhaem.raw$mass_tet_exp, order = FALSE,
                                    levels = c(0, 1),
                                    labels = c("no", "yes"))
mhaem.factor$mass_corr_exp <- factor(mhaem.raw$mass_corr_exp, order = FALSE,
                                    levels = c(0, 1),
                                    labels = c("no", "yes"))
mhaem.factor$mass_rum_exp <- factor(mhaem.raw$mass_rum_exp, order = FALSE,
                                    levels = c(0, 1),
                                    labels = c("no", "yes"))
mhaem.factor$mass_sul_exp <- factor(mhaem.raw$mass_sul_exp, order = FALSE,
                                    levels = c(0, 1),
                                    labels = c("no", "yes"))
mhaem.factor$feed_lane_cleaning <- factor(mhaem.raw$feed_lane_cleaning, order = FALSE,
                                          levels = c(1, 2, 3, 4),
                                          labels = c("scrape", "lagoon", "clean", "sl"))
mhaem.factor$dust_mgmt <- factor(mhaem.raw$dust_mgmt, order = FALSE,
                                 levels = c(0, 1),
                                 labels = c("no", "yes"))
mhaem.factor$mort_rate <- factor(mhaem.raw$mort_rate, order = FALSE,
                                 levels = c(0, 1),
                                 labels = c("no", "yes"))
mhaem.factor$pen_trx_rate <- factor(mhaem.raw$pen_trx_rate, order = FALSE,
                                    levels = c(1, 2),
                                    labels = c("low", "high"))
mhaem.factor$cef_trx_rate <- factor(mhaem.raw$cef_trx_rate, order = FALSE,
                                    levels = c(1, 2),
                                    labels = c("low", "high"))
mhaem.factor$mac_trx_rate <- factor(mhaem.raw$mac_trx_rate, order = FALSE,
                                    levels = c(1, 2),
                                    labels = c("low", "high"))
mhaem.factor$res_nuf_trx_rate <- factor(mhaem.raw$res_nuf_trx_rate, order = FALSE,
                                        levels = c(1, 2),
                                        labels = c("low", "high"))
mhaem.factor$tet_inj_use <- factor(mhaem.raw$tet_inj_use, order = FALSE,
                                  levels = c(0, 1),
                                  labels = c("no", "yes"))
mhaem.factor$sul_trx_rate <- factor(mhaem.raw$sul_trx_rate, order = FALSE,
                                    levels = c(1, 2),
                                    labels = c("low", "high"))
mhaem.factor$bay_trx_rate <- factor(mhaem.raw$bay_trx_rate, order = FALSE,
                                    levels = c(1, 2),
                                    labels = c("low", "high"))
mhaem.factor$Farm <- factor(mhaem.raw$Farm, order = FALSE,
                            levels = c(1, 2, 3, 4, 5, 6),
                            labels = c("A", "B", "C", "D", "E", "F"))
mhaem.factor$last_treat <- factor(mhaem.raw$last_treat, order = FALSE,
                                  levels = c(1, 2, 3, 4),
                                  labels = c("<16 days", "16-60 days", 
                                             ">=60 days", "no trx"))
mhaem.factor$no_classes <- factor(mhaem.raw$no_classes, order = FALSE,
                                  levels = c(0, 1, 2, 3),
                                  labels = c("0", "1", "2", "3 or more"))
mhaem.factor$total_abx <- factor(mhaem.raw$total_abx, order = FALSE,
                                  levels = c(0, 1, 2),
                                  labels = c("0", "1 to 2", "3 or more"))
mhaem.factor$days_since_pen <- factor(mhaem.raw$days_since_pen, order = FALSE,
                                      levels = c(1, 2, 3, 4),
                                      labels = c("<16 days", "16-60 days", 
                                                 ">=60 days", "no trx"))
mhaem.factor$days_since_ceph <- factor(mhaem.raw$days_since_ceph, order = FALSE,
                                       levels = c(1, 2, 3, 4),
                                       labels = c("<16 days", "16-60 days", 
                                                  ">=60 days", "no trx"))
mhaem.factor$days_since_mac <- factor(mhaem.raw$days_since_mac, order = FALSE,
                                      levels = c(1, 2, 3, 4),
                                      labels = c("<16 days", "16-60 days", 
                                                 ">=60 days", "no trx"))
mhaem.factor$days_since_phen <- factor(mhaem.raw$days_since_phen, order = FALSE,
                                       levels = c(1, 2, 3, 4),
                                       labels = c("<16 days", "16-60 days", 
                                                  ">=60 days", "no trx"))
mhaem.factor$days_since_tet <- factor(mhaem.raw$days_since_tet, order = FALSE,
                                      levels = c(2, 3, 4),
                                      labels = c("16-60 days", ">=60 days", "no trx"))
mhaem.factor$days_since_sul <- factor(mhaem.raw$days_since_sul, order = FALSE,
                                      levels = c(3, 4),
                                      labels = c(">=60 days", "no trx"))
mhaem.factor$days_since_flr <- factor(mhaem.raw$days_since_flr, order = FALSE,
                                      levels = c(1, 2, 3, 4),
                                      labels = c("<16 days", "16-60 days", 
                                                 ">=60 days", "no trx"))
```

```
# analytic data frame for all farms
mhaem.all <- mhaem.factor %>%
            select(-"srp", - "entervene", -"pen_trx_rate", -"brd",
                   -"cef_trx_rate", -"mac_trx_rate", -"res_nuf_trx_rate",
                   -"tet_inj_use", -"sul_trx_rate", -"bay_trx_rate", -"mort_rate", 
                   - "calf_ID", -"Farm_ID", -"total_abx", 
                   -"last_treat", -"days_since_pen", -"days_since_ceph", 
                   -"days_since_mac", -"days_since_phen", -"days_since_tet", 
                   -"days_since_sul",  -"days_since_flr")

# check for missingness
summary(aggr(mhaem.all))
```

```
## 
##  Missings per variable: 
##               Variable Count
##                   Farm     0
##                 season     0
##                    cef     0
##                    dan     0
##                    enr     0
##                    flr     0
##                    gam     0
##                    gen     0
##                    pen     0
##                    spc     0
##                    tet     0
##                   tild     0
##                   tilm     0
##                    tul     0
##              source_no     0
##         onsite_milking     0
##              resp_vacc     0
##             salmonella     0
##              first_def     0
##             bovishield     0
##                pyramid     0
##               titanium     0
##                pinkeye     0
##               clostrid     0
##          colost_source     0
##            colost_past     0
##           colost_again     0
##  hosp_milk_pastuerized     0
##                 action     0
##            bull_calves     0
##          milk_mass_med     0
##           mass_pen_exp     0
##           mass_tet_exp     0
##           mass_rum_exp     0
##          mass_corr_exp     0
##           mass_sul_exp     0
##     feed_lane_cleaning     0
##              dust_mgmt     0
##             no_classes     0
## 
##  Missings in combinations of variables: 
##                                                                   Combinations
##  0:0:0:0:0:0:0:0:0:0:0:0:0:0:0:0:0:0:0:0:0:0:0:0:0:0:0:0:0:0:0:0:0:0:0:0:0:0:0
##  Count Percent
##    119     100
```

```
# check cell counts - All Farms
summary(mhaem.all) # cef, gen, resp_vacc, colost_source, colost_past, hosp_milk_pastuerized, feed_lane_cleaning(sl), dust_mgmt, no_classes(3 or more) below 10
```

```
##  Farm      season   cef     dan    enr    flr    gam    gen     pen    spc    
##  A:23   summer:60   S:119   S:36   S:34   S:79   S:75   S:112   S:74   S:100  
##  B:26   winter:59   R:  0   R:83   R:85   R:40   R:44   R:  7   R:45   R: 19  
##  C:17                                                                         
##  D:20                                                                         
##  E: 9                                                                         
##  F:24                                                                         
##  tet     tild   tilm   tul         source_no  onsite_milking resp_vacc
##  S: 16   S:55   S:43   S:84   single    :44   no :40         no :  9  
##  R:103   R:64   R:76   R:35   low multi :49   yes:79         yes:110  
##                               high multi:26                           
##                                                                       
##                                                                       
##                                                                       
##  salmonella first_def bovishield pyramid  titanium pinkeye  clostrid
##  no :53     no :95    no :61     no :78   no :99   no :32   no :99  
##  yes:66     yes:24    yes:58     yes:41   yes:20   yes:87   yes:20  
##                                                                     
##                                                                     
##                                                                     
##                                                                     
##  colost_source colost_past colost_again hosp_milk_pastuerized     action   
##  p :110        no :  9     no :32       no :  9               screen :102  
##  dp:  9        yes:110     yes:43       yes:110               premium: 17  
##                            -99:44                                          
##                                                                            
##                                                                            
##                                                                            
##  bull_calves milk_mass_med mass_pen_exp mass_tet_exp mass_rum_exp mass_corr_exp
##  no :52      no :63        no :96       no :24       no :23       no :67       
##  yes:67      yes:56        yes:23       yes:95       yes:96       yes:52       
##                                                                                
##                                                                                
##                                                                                
##                                                                                
##  mass_sul_exp feed_lane_cleaning dust_mgmt     no_classes
##  no :33       scrape:40          no :  9   0        :38  
##  yes:86       lagoon:50          yes:110   1        :55  
##               clean :20                    2        :26  
##               sl    : 9                    3 or more: 0  
##                                                          
##
```

```
# remove variables
mhaemall.analyze <- as.data.frame(mhaem.all)

mhaemall.analyze <- as.data.frame(mhaem.all) %>%
                    select(-"cef", -"gen", -"resp_vacc", -"colost_source", -"colost_past",
                           -"colost_again", -"hosp_milk_pastuerized", -"dust_mgmt") %>%
            droplevels(mhaemall.analyze$no_classes, exclude = "3 or more")

# recategorize feed_lane_cleaning
mhaemall.analyze$feed_lane_cleaning <- factor(mhaem.raw$feed_lane_cleaning, order = FALSE,
                                          levels = c(1, 2, 3, 4),
                                          labels = c("scrape/lagoon", "scrape/lagoon", 
                                                     "clean", "scrape/lagoon"))
# check
summary(mhaemall.analyze)
```

```
##  Farm      season   dan    enr    flr    gam    pen    spc     tet     tild  
##  A:23   summer:60   S:36   S:34   S:79   S:75   S:74   S:100   S: 16   S:55  
##  B:26   winter:59   R:83   R:85   R:40   R:44   R:45   R: 19   R:103   R:64  
##  C:17                                                                        
##  D:20                                                                        
##  E: 9                                                                        
##  F:24                                                                        
##  tilm   tul         source_no  onsite_milking salmonella first_def bovishield
##  S:43   S:84   single    :44   no :40         no :53     no :95    no :61    
##  R:76   R:35   low multi :49   yes:79         yes:66     yes:24    yes:58    
##                high multi:26                                                 
##                                                                              
##                                                                              
##                                                                              
##  pyramid  titanium pinkeye  clostrid     action    bull_calves milk_mass_med
##  no :78   no :99   no :32   no :99   screen :102   no :52      no :63       
##  yes:41   yes:20   yes:87   yes:20   premium: 17   yes:67      yes:56       
##                                                                             
##                                                                             
##                                                                             
##                                                                             
##  mass_pen_exp mass_tet_exp mass_rum_exp mass_corr_exp mass_sul_exp
##  no :96       no :24       no :23       no :67        no :33      
##  yes:23       yes:95       yes:96       yes:52        yes:86      
##                                                                   
##                                                                   
##                                                                   
##                                                                   
##      feed_lane_cleaning no_classes
##  scrape/lagoon:99       0:38      
##  clean        :20       1:55      
##                         2:26      
##                                   
##                                   
##
```

```
# create levels
mhaem.bl.all = tiers2blacklist(list("season", "Farm",
                     c("dan", "enr", "flr", "gam", "pen", "spc", "tet", "tild", "tilm", 
                       "tul", "source_no", "onsite_milking", "first_def", 
                       "salmonella","bovishield", "pyramid", "titanium", "pinkeye", 
                       "clostrid", "action", "bull_calves", 
                       "milk_mass_med", "mass_pen_exp", "mass_tet_exp", "mass_rum_exp", 
                       "mass_corr_exp", "mass_sul_exp","feed_lane_cleaning",
                       "no_classes")))
mhaem.bl.all = rbind(mhaem.bl.all, c("season"))
mhaem.bl.all
```

```
##       from                 to      
##  [1,] "Farm"               "season"
##  [2,] "dan"                "season"
##  [3,] "enr"                "season"
##  [4,] "flr"                "season"
##  [5,] "gam"                "season"
##  [6,] "pen"                "season"
##  [7,] "spc"                "season"
##  [8,] "tet"                "season"
##  [9,] "tild"               "season"
## [10,] "tilm"               "season"
## [11,] "tul"                "season"
## [12,] "source_no"          "season"
## [13,] "onsite_milking"     "season"
## [14,] "first_def"          "season"
## [15,] "salmonella"         "season"
## [16,] "bovishield"         "season"
## [17,] "pyramid"            "season"
## [18,] "titanium"           "season"
## [19,] "pinkeye"            "season"
## [20,] "clostrid"           "season"
## [21,] "action"             "season"
## [22,] "bull_calves"        "season"
## [23,] "milk_mass_med"      "season"
## [24,] "mass_pen_exp"       "season"
## [25,] "mass_tet_exp"       "season"
## [26,] "mass_rum_exp"       "season"
## [27,] "mass_corr_exp"      "season"
## [28,] "mass_sul_exp"       "season"
## [29,] "feed_lane_cleaning" "season"
## [30,] "no_classes"         "season"
## [31,] "dan"                "Farm"  
## [32,] "enr"                "Farm"  
## [33,] "flr"                "Farm"  
## [34,] "gam"                "Farm"  
## [35,] "pen"                "Farm"  
## [36,] "spc"                "Farm"  
## [37,] "tet"                "Farm"  
## [38,] "tild"               "Farm"  
## [39,] "tilm"               "Farm"  
## [40,] "tul"                "Farm"  
## [41,] "source_no"          "Farm"  
## [42,] "onsite_milking"     "Farm"  
## [43,] "first_def"          "Farm"  
## [44,] "salmonella"         "Farm"  
## [45,] "bovishield"         "Farm"  
## [46,] "pyramid"            "Farm"  
## [47,] "titanium"           "Farm"  
## [48,] "pinkeye"            "Farm"  
## [49,] "clostrid"           "Farm"  
## [50,] "action"             "Farm"  
## [51,] "bull_calves"        "Farm"  
## [52,] "milk_mass_med"      "Farm"  
## [53,] "mass_pen_exp"       "Farm"  
## [54,] "mass_tet_exp"       "Farm"  
## [55,] "mass_rum_exp"       "Farm"  
## [56,] "mass_corr_exp"      "Farm"  
## [57,] "mass_sul_exp"       "Farm"  
## [58,] "feed_lane_cleaning" "Farm"  
## [59,] "no_classes"         "Farm"  
## [60,] "season"             "season"
```

#### 2.2 BNA

```
# all farms hc structure learning
mhaemfarm.hc <- hc(mhaemall.analyze, blacklist = mhaem.bl.all, whitelist = wl, score = "aic")

# plot customization
mhaem.a <- Rgraphviz::layoutGraph(bnlearn::as.graphNEL(mhaemfarm.hc))
graph::nodeRenderInfo(mhaem.a) <- list(fontsize=60)
Rgraphviz::renderGraph(mhaem.a)
```

```
# all farms tabu structure learning
mhaemfarm.tabu <- tabu(mhaemall.analyze, blacklist = mhaem.bl.all, 
                       whitelist = wl, score = "aic")

# plot customization
mhaem.b <- Rgraphviz::layoutGraph(bnlearn::as.graphNEL(mhaemfarm.tabu))
graph::nodeRenderInfo(mhaem.b) <- list(fontsize=60)
Rgraphviz::renderGraph(mhaem.b)
```

```
graphviz.compare(mhaemfarm.hc, mhaemfarm.tabu, shape = "ellipse", 
                 main= c("Mhaem Farms HC DAG", "Mhaem Farms Tabu DAG"))
```

```
# bootstrap
mhaemfarm.boot = boot.strength(mhaemall.analyze, R = 10000, algorithm = "hc", 
                        algorithm.args = list(blacklist = mhaem.bl.all, 
                                              whitelist = wl, score = "aic"))

# take arcs with strength of 50% and return average consensus network
mhaemfarm.avg = averaged.network(mhaemfarm.boot, threshold = 0.5)

# graph customization
mhaem.c <- Rgraphviz::layoutGraph(bnlearn::as.graphNEL(mhaemfarm.avg))
graph::nodeRenderInfo(mhaem.c) <- list(fontsize=60)
Rgraphviz::renderGraph(mhaem.c)
```

```
# plot the bootstrapped averaged plot with the original for comparison
graphviz.compare(mhaemfarm.avg, mhaemfarm.hc, shape = "ellipse", 
                 main= c("Mhaem Farm Level Factors Averaged DAG", 
                         "Mhaem Farm Level Factors Single DAG"))
```

### Animal level factors

#### 2.3 Data Wrangling

```
# analytic data frame for individual risk factors
mhaem.ind <- mhaem.factor %>%
            select("Farm", "season", "brd", "dan", "enr", "flr", "gam", "pen", "spc", 
                   "tet", "tild", "tilm", "tul", "total_abx", "last_treat", 
                   "days_since_pen", "days_since_ceph", "days_since_mac", "days_since_phen",
                   "days_since_tet", "days_since_sul",  "days_since_flr")

# check for missingness
summary(aggr(mhaem.ind))
```

```
## 
##  Missings per variable: 
##         Variable Count
##             Farm     0
##           season     0
##              brd     0
##              dan     0
##              enr     0
##              flr     0
##              gam     0
##              pen     0
##              spc     0
##              tet     0
##             tild     0
##             tilm     0
##              tul     0
##        total_abx     9
##       last_treat     9
##   days_since_pen     9
##  days_since_ceph     9
##   days_since_mac     9
##  days_since_phen     9
##   days_since_tet     9
##   days_since_sul     9
##   days_since_flr     9
## 
##  Missings in combinations of variables: 
##                                 Combinations Count   Percent
##  0:0:0:0:0:0:0:0:0:0:0:0:0:0:0:0:0:0:0:0:0:0   110 92.436975
##  0:0:0:0:0:0:0:0:0:0:0:0:0:1:1:1:1:1:1:1:1:1     9  7.563025
```

```
# check cell counts - All Farms
summary(mhaem.ind) # days_since_pen(pen yes/no), days_since_ceph(ceph yes/no), days_since_mac(mac yes/no), days_since_phen(0-60 days, >=60 days, no trx), days_since_tet(tet yes/no), days_since_sul(remove), days_since_flr(0-60 days, >= 60 days, no trx)
```

```
##  Farm      season    brd     dan    enr    flr    gam    pen    spc     tet    
##  A:23   summer:60   neg:56   S:36   S:34   S:79   S:75   S:74   S:100   S: 16  
##  B:26   winter:59   pos:63   R:83   R:85   R:40   R:44   R:45   R: 19   R:103  
##  C:17                                                                          
##  D:20                                                                          
##  E: 9                                                                          
##  F:24                                                                          
##  tild   tilm   tul        total_abx       last_treat    days_since_pen
##  S:55   S:43   S:84   0        :30   <16 days  :14   <16 days  : 3    
##  R:64   R:76   R:35   1 to 2   :35   16-60 days:25   16-60 days: 4    
##                       3 or more:45   >=60 days :42   >=60 days :11    
##                       NA's     : 9   no trx    :29   no trx    :92    
##                                      NA's      : 9   NA's      : 9    
##                                                                       
##    days_since_ceph    days_since_mac   days_since_phen    days_since_tet
##  <16 days  :  1    <16 days  : 2     <16 days  : 8     16-60 days: 0    
##  16-60 days:  2    16-60 days: 5     16-60 days:16     >=60 days :15    
##  >=60 days :  7    >=60 days :17     >=60 days :34     no trx    :95    
##  no trx    :100    no trx    :86     no trx    :52     NA's      : 9    
##  NA's      :  9    NA's      : 9     NA's      : 9                      
##                                                                         
##    days_since_sul    days_since_flr
##  >=60 days:  7    <16 days  : 2    
##  no trx   :103    16-60 days:11    
##  NA's     :  9    >=60 days :16    
##                   no trx    :81    
##                   NA's      : 9    
##
```

```
# remove variables
mhaemind2 <- as.data.frame(mhaem.ind) %>%
                    select(-"days_since_sul")

# check
summary(mhaemind2)
```

```
##  Farm      season    brd     dan    enr    flr    gam    pen    spc     tet    
##  A:23   summer:60   neg:56   S:36   S:34   S:79   S:75   S:74   S:100   S: 16  
##  B:26   winter:59   pos:63   R:83   R:85   R:40   R:44   R:45   R: 19   R:103  
##  C:17                                                                          
##  D:20                                                                          
##  E: 9                                                                          
##  F:24                                                                          
##  tild   tilm   tul        total_abx       last_treat    days_since_pen
##  S:55   S:43   S:84   0        :30   <16 days  :14   <16 days  : 3    
##  R:64   R:76   R:35   1 to 2   :35   16-60 days:25   16-60 days: 4    
##                       3 or more:45   >=60 days :42   >=60 days :11    
##                       NA's     : 9   no trx    :29   no trx    :92    
##                                      NA's      : 9   NA's      : 9    
##                                                                       
##    days_since_ceph    days_since_mac   days_since_phen    days_since_tet
##  <16 days  :  1    <16 days  : 2     <16 days  : 8     16-60 days: 0    
##  16-60 days:  2    16-60 days: 5     16-60 days:16     >=60 days :15    
##  >=60 days :  7    >=60 days :17     >=60 days :34     no trx    :95    
##  no trx    :100    no trx    :86     no trx    :52     NA's      : 9    
##  NA's      :  9    NA's      : 9     NA's      : 9                      
##                                                                         
##     days_since_flr
##  <16 days  : 2    
##  16-60 days:11    
##  >=60 days :16    
##  no trx    :81    
##  NA's      : 9    
##
```

```
# recategorize
mhaemind2$days_since_pen <- factor(mhaem.raw$days_since_pen, order = FALSE,
                                          levels = c(1, 2, 3, 4),
                                          labels = c("treated", "treated", 
                                                     "treated", "no treatment"))
mhaemind2$days_since_ceph <- factor(mhaem.raw$days_since_ceph, order = FALSE,
                                          levels = c(1, 2, 3, 4),
                                          labels = c("treated", "treated", 
                                                     "treated", "no treatment"))
mhaemind2$days_since_mac <- factor(mhaem.raw$days_since_mac, order = FALSE,
                                          levels = c(1, 2, 3, 4),
                                          labels = c("treated", "treated", 
                                                     "treated", "no treatment"))
mhaemind2$days_since_phen <- factor(mhaem.raw$days_since_phen, order = FALSE,
                                          levels = c(1, 2, 3, 4),
                                          labels = c("0-60 days", "0-60 days", 
                                                     ">=60 days", "no treatment"))
mhaemind2$days_since_tet <- factor(mhaem.raw$days_since_tet, order = FALSE,
                                          levels = c(1, 2, 3, 4),
                                          labels = c("treated", "treated", 
                                                     "treated", "no treatment"))
mhaemind2$days_since_flr <- factor(mhaem.raw$days_since_flr, order = FALSE,
                                          levels = c(1, 2, 3, 4),
                                          labels = c("0-60 days", "0-60 days", 
                                                     ">=60 days", "no treatment"))

# remove Farm E
mhaemind2$Farm <- factor(mhaem.raw$Farm, order = FALSE,
                            levels = c(1, 2, 3, 4, 6),
                            labels = c("A", "B", "C", "D", "F"))
# summary(mhaemind2)

mhaemind.analyze <- na.omit(mhaemind2)

# check
summary(mhaemind.analyze)
```

```
##  Farm      season    brd     dan    enr    flr    gam    pen    spc    tet   
##  A:23   summer:55   neg:50   S:35   S:34   S:72   S:71   S:70   S:93   S:15  
##  B:26   winter:55   pos:60   R:75   R:76   R:38   R:39   R:40   R:17   R:95  
##  C:17                                                                        
##  D:20                                                                        
##  F:24                                                                        
##  tild   tilm   tul        total_abx       last_treat      days_since_pen
##  S:55   S:42   S:78   0        :30   <16 days  :14   treated     :18    
##  R:55   R:68   R:32   1 to 2   :35   16-60 days:25   no treatment:92    
##                       3 or more:45   >=60 days :42                      
##                                      no trx    :29                      
##                                                                         
##      days_since_ceph      days_since_mac     days_since_phen
##  treated     : 10    treated     :24     0-60 days   :24    
##  no treatment:100    no treatment:86     >=60 days   :34    
##                                          no treatment:52    
##                                                             
##                                                             
##       days_since_tet      days_since_flr
##  treated     :15     0-60 days   :13    
##  no treatment:95     >=60 days   :16    
##                      no treatment:81    
##                                         
##
```

```
# create levels
mhaem.bl.ind = tiers2blacklist(list("season", "Farm",
                     c("brd", "dan", "enr", "flr", "gam", "pen", "spc", "tet", "tild", "tilm", 
                       "tul", "total_abx", "last_treat", "days_since_pen", 
                       "days_since_ceph", "days_since_mac", "days_since_phen",
                       "days_since_tet", "days_since_flr")))
mhaem.bl.ind = rbind(mhaem.bl.ind, c("season"))
mhaem.bl.ind
```

```
##       from              to      
##  [1,] "Farm"            "season"
##  [2,] "brd"             "season"
##  [3,] "dan"             "season"
##  [4,] "enr"             "season"
##  [5,] "flr"             "season"
##  [6,] "gam"             "season"
##  [7,] "pen"             "season"
##  [8,] "spc"             "season"
##  [9,] "tet"             "season"
## [10,] "tild"            "season"
## [11,] "tilm"            "season"
## [12,] "tul"             "season"
## [13,] "total_abx"       "season"
## [14,] "last_treat"      "season"
## [15,] "days_since_pen"  "season"
## [16,] "days_since_ceph" "season"
## [17,] "days_since_mac"  "season"
## [18,] "days_since_phen" "season"
## [19,] "days_since_tet"  "season"
## [20,] "days_since_flr"  "season"
## [21,] "brd"             "Farm"  
## [22,] "dan"             "Farm"  
## [23,] "enr"             "Farm"  
## [24,] "flr"             "Farm"  
## [25,] "gam"             "Farm"  
## [26,] "pen"             "Farm"  
## [27,] "spc"             "Farm"  
## [28,] "tet"             "Farm"  
## [29,] "tild"            "Farm"  
## [30,] "tilm"            "Farm"  
## [31,] "tul"             "Farm"  
## [32,] "total_abx"       "Farm"  
## [33,] "last_treat"      "Farm"  
## [34,] "days_since_pen"  "Farm"  
## [35,] "days_since_ceph" "Farm"  
## [36,] "days_since_mac"  "Farm"  
## [37,] "days_since_phen" "Farm"  
## [38,] "days_since_tet"  "Farm"  
## [39,] "days_since_flr"  "Farm"  
## [40,] "season"          "season"
```

#### 2.4 BNA

```
# individual factors hc structure learning
mhaemind.hc <- hc(mhaemind.analyze, blacklist = mhaem.bl.ind, 
                  whitelist = wl, score = "aic")

# plot customization
mhaem.c <- Rgraphviz::layoutGraph(bnlearn::as.graphNEL(mhaemind.hc))
graph::nodeRenderInfo(mhaem.c) <- list(fontsize=60)
Rgraphviz::renderGraph(mhaem.c)
```

```
# individual factors tabu structure learning
mhaemind.tabu <- tabu(mhaemind.analyze, blacklist = mhaem.bl.ind, 
                      whitelist = wl, score = "aic")

# plot customization
mhaem.d <- Rgraphviz::layoutGraph(bnlearn::as.graphNEL(mhaemind.tabu))
graph::nodeRenderInfo(mhaem.d) <- list(fontsize=60)
Rgraphviz::renderGraph(mhaem.d)
```

```
graphviz.compare(mhaemind.hc, mhaemind.tabu, shape = "ellipse", 
                 main= c("Mhaem Individual Risk Factors HC DAG", 
                         "Mhaem Individual Risk Factors Tabu DAG"))
```

```
# bootstrap
mhaemind.boot = boot.strength(mhaemind.analyze, R = 10000, algorithm = "hc", 
                        algorithm.args = list(blacklist = mhaem.bl.ind, 
                                              whitelist = wl, score = "aic"))

# take arcs with strength of 50% and return average consensus network
mhaemind.avg = averaged.network(mhaemind.boot, threshold = 0.5)

# graph customization
mhaem.e <- Rgraphviz::layoutGraph(bnlearn::as.graphNEL(mhaemind.avg))
graph::nodeRenderInfo(mhaem.e) <- list(fontsize=40)
Rgraphviz::renderGraph(mhaem.e)
```

```
# plot the bootstrapped averaged plot with the original for comparison
graphviz.compare(mhaemind.avg, mhaemind.hc, shape = "ellipse", 
                 main= c("M haem Individual Level Factors Averaged DAG", 
                         "M haem Individual Level Factors Single DAG"))
```

#### 2.5 Parameter Learning

Farm level query

```
# fit the parameters - bayesian method
mhaemfarm.bayes = bn.fit(mhaemfarm.avg, mhaemall.analyze, method = "bayes")

# probability table
mhaemfarm.bayes
```

```
## 
##   Bayesian network parameters
## 
##   Parameters of node Farm (multinomial distribution)
## 
## Conditional probability table:
##  
##     season
## Farm     summer     winter
##    A 0.10055096 0.28711485
##    B 0.26584022 0.16946779
##    C 0.13360882 0.15266106
##    D 0.21625344 0.11904762
##    E 0.08402204 0.06862745
##    F 0.19972452 0.20308123
## 
##   Parameters of node season (multinomial distribution)
## 
## Conditional probability table:
##     summer    winter 
## 0.5041667 0.4958333 
## 
##   Parameters of node dan (multinomial distribution)
## 
## Conditional probability table:
##  
##    Farm
## dan           A           B           C           D           E           F
##   S 0.089928058 0.079617834 0.179611650 0.995867769 0.118181818 0.334482759
##   R 0.910071942 0.920382166 0.820388350 0.004132231 0.881818182 0.665517241
## 
##   Parameters of node enr (multinomial distribution)
## 
## Conditional probability table:
##  
## , , pinkeye = no
## 
##    dan
## enr           S           R
##   S 0.346153846 0.004273504
##   R 0.653846154 0.995726496
## 
## , , pinkeye = yes
## 
##    dan
## enr           S           R
##   S 0.996240602 0.002304147
##   R 0.003759398 0.997695853
## 
## 
##   Parameters of node flr (multinomial distribution)
## 
## Conditional probability table:
##  
## , , pen = S, tul = S
## 
##    Farm
## flr           A           B           C           D           E           F
##   S 0.998960499 0.998402556 0.797520661 0.980000000 0.993150685 0.998113208
##   R 0.001039501 0.001597444 0.202479339 0.020000000 0.006849315 0.001886792
## 
## , , pen = R, tul = S
## 
##    Farm
## flr           A           B           C           D           E           F
##   S 0.993150685 0.989795918 0.989795918 0.263676149 0.993150685 0.989795918
##   R 0.006849315 0.010204082 0.010204082 0.736323851 0.006849315 0.010204082
## 
## , , pen = S, tul = R
## 
##    Farm
## flr           A           B           C           D           E           F
##   S 0.500000000 0.334482759 0.335616438 0.500000000 0.980000000 0.545283019
##   R 0.500000000 0.665517241 0.664383562 0.500000000 0.020000000 0.454716981
## 
## , , pen = R, tul = R
## 
##    Farm
## flr           A           B           C           D           E           F
##   S 0.500000000 0.004132231 0.002958580 0.500000000 0.010204082 0.500000000
##   R 0.500000000 0.995867769 0.997041420 0.500000000 0.989795918 0.500000000
## 
## 
##   Parameters of node gam (multinomial distribution)
## 
## Conditional probability table:
##  
##    Farm
## gam           A           B           C           D           E           F
##   S 0.996402878 0.576433121 0.354368932 0.995867769 0.445454545 0.293103448
##   R 0.003597122 0.423566879 0.645631068 0.004132231 0.554545455 0.706896552
## 
##   Parameters of node pen (multinomial distribution)
## 
## Conditional probability table:
##  
##    Farm
## pen          A          B          C          D          E          F
##   S 0.86690647 0.72929936 0.47087379 0.05371901 0.44545455 0.91379310
##   R 0.13309353 0.27070064 0.52912621 0.94628099 0.55454545 0.08620690
## 
##   Parameters of node spc (multinomial distribution)
## 
## Conditional probability table:
##  
##    tilm
## spc           S           R
##   S 0.994252874 0.748366013
##   R 0.005747126 0.251633987
## 
##   Parameters of node tet (multinomial distribution)
## 
## Conditional probability table:
##  
##    enr
## tet          S          R
##   S 0.44202899 0.01461988
##   R 0.55797101 0.98538012
## 
##   Parameters of node tild (multinomial distribution)
## 
## Conditional probability table:
##  
## , , gam = S
## 
##     enr
## tild           S           R
##    S 0.996240602 0.500000000
##    R 0.003759398 0.500000000
## 
## , , gam = R
## 
##     enr
## tild           S           R
##    S 0.100000000 0.026011561
##    R 0.900000000 0.973988439
## 
## 
##   Parameters of node tilm (multinomial distribution)
## 
## Conditional probability table:
##  
## , , tild = S
## 
##     pen
## tilm           S           R
##    S 0.932000000 0.541237113
##    R 0.068000000 0.458762887
## 
## , , tild = R
## 
##     pen
## tilm           S           R
##    S 0.002890173 0.052941176
##    R 0.997109827 0.947058824
## 
## 
##   Parameters of node tul (multinomial distribution)
## 
## Conditional probability table:
##  
##    gam
## tul          S          R
##   S 0.98344371 0.23033708
##   R 0.01655629 0.76966292
## 
##   Parameters of node source_no (multinomial distribution)
## 
## Conditional probability table:
##  
##             Farm
## source_no              A           B           C           D           E
##   single     0.002398082 0.002123142 0.003236246 0.994490358 0.006060606
##   low multi  0.995203837 0.995753715 0.003236246 0.002754821 0.006060606
##   high multi 0.002398082 0.002123142 0.993527508 0.002754821 0.987878788
##             Farm
## source_no              F
##   single     0.995402299
##   low multi  0.002298851
##   high multi 0.002298851
## 
##   Parameters of node onsite_milking (multinomial distribution)
## 
## Conditional probability table:
##  
##               Farm
## onsite_milking           A           B           C           D           E
##            no  0.996402878 0.003184713 0.995145631 0.004132231 0.009090909
##            yes 0.003597122 0.996815287 0.004854369 0.995867769 0.990909091
##               Farm
## onsite_milking           F
##            no  0.003448276
##            yes 0.996551724
## 
##   Parameters of node salmonella (multinomial distribution)
## 
## Conditional probability table:
##  
##           Farm
## salmonella           A           B           C           D           E
##        no  0.003597122 0.003184713 0.004854369 0.995867769 0.990909091
##        yes 0.996402878 0.996815287 0.995145631 0.004132231 0.009090909
##           Farm
## salmonella           F
##        no  0.996551724
##        yes 0.003448276
## 
##   Parameters of node first_def (multinomial distribution)
## 
## Conditional probability table:
##  
##          Farm
## first_def           A           B           C           D           E
##       no  0.996402878 0.996815287 0.995145631 0.995867769 0.990909091
##       yes 0.003597122 0.003184713 0.004854369 0.004132231 0.009090909
##          Farm
## first_def           F
##       no  0.003448276
##       yes 0.996551724
## 
##   Parameters of node bovishield (multinomial distribution)
## 
## Conditional probability table:
##  
##           Farm
## bovishield           A           B           C           D           E
##        no  0.003597122 0.003184713 0.995145631 0.995867769 0.009090909
##        yes 0.996402878 0.996815287 0.004854369 0.004132231 0.990909091
##           Farm
## bovishield           F
##        no  0.996551724
##        yes 0.003448276
## 
##   Parameters of node pyramid (multinomial distribution)
## 
## Conditional probability table:
##  
##        Farm
## pyramid           A           B           C           D           E           F
##     no  0.996402878 0.996815287 0.004854369 0.995867769 0.990909091 0.003448276
##     yes 0.003597122 0.003184713 0.995145631 0.004132231 0.009090909 0.996551724
## 
##   Parameters of node titanium (multinomial distribution)
## 
## Conditional probability table:
##  
##         Farm
## titanium           A           B           C           D           E
##      no  0.996402878 0.996815287 0.995145631 0.004132231 0.990909091
##      yes 0.003597122 0.003184713 0.004854369 0.995867769 0.009090909
##         Farm
## titanium           F
##      no  0.996551724
##      yes 0.003448276
## 
##   Parameters of node pinkeye (multinomial distribution)
## 
## Conditional probability table:
##  
##        Farm
## pinkeye           A           B           C           D           E           F
##     no  0.996402878 0.003184713 0.004854369 0.004132231 0.990909091 0.003448276
##     yes 0.003597122 0.996815287 0.995145631 0.995867769 0.009090909 0.996551724
## 
##   Parameters of node clostrid (multinomial distribution)
## 
## Conditional probability table:
##  
##         titanium
## clostrid          no         yes
##      no  0.997487437 0.012195122
##      yes 0.002512563 0.987804878
## 
##   Parameters of node action (multinomial distribution)
## 
## Conditional probability table:
##  
##          Farm
## action              A           B           C           D           E
##   screen  0.996402878 0.996815287 0.004854369 0.995867769 0.990909091
##   premium 0.003597122 0.003184713 0.995145631 0.004132231 0.009090909
##          Farm
## action              F
##   screen  0.996551724
##   premium 0.003448276
## 
##   Parameters of node bull_calves (multinomial distribution)
## 
## Conditional probability table:
##  
##            Farm
## bull_calves           A           B           C           D           E
##         no  0.003597122 0.996815287 0.995145631 0.004132231 0.990909091
##         yes 0.996402878 0.003184713 0.004854369 0.995867769 0.009090909
##            Farm
## bull_calves           F
##         no  0.003448276
##         yes 0.996551724
## 
##   Parameters of node milk_mass_med (multinomial distribution)
## 
## Conditional probability table:
##  
##              Farm
## milk_mass_med           A           B           C           D           E
##           no  0.003597122 0.996815287 0.995145631 0.995867769 0.009090909
##           yes 0.996402878 0.003184713 0.004854369 0.004132231 0.990909091
##              Farm
## milk_mass_med           F
##           no  0.003448276
##           yes 0.996551724
## 
##   Parameters of node mass_pen_exp (multinomial distribution)
## 
## Conditional probability table:
##  
##             Farm
## mass_pen_exp           A           B           C           D           E
##          no  0.003597122 0.996815287 0.995145631 0.995867769 0.990909091
##          yes 0.996402878 0.003184713 0.004854369 0.004132231 0.009090909
##             Farm
## mass_pen_exp           F
##          no  0.996551724
##          yes 0.003448276
## 
##   Parameters of node mass_tet_exp (multinomial distribution)
## 
## Conditional probability table:
##  
##             first_def
## mass_tet_exp          no         yes
##          no  0.002617801 0.989795918
##          yes 0.997382199 0.010204082
## 
##   Parameters of node mass_rum_exp (multinomial distribution)
## 
## Conditional probability table:
##  
##             mass_pen_exp
## mass_rum_exp          no         yes
##          no  0.002590674 0.989361702
##          yes 0.997409326 0.010638298
## 
##   Parameters of node mass_corr_exp (multinomial distribution)
## 
## Conditional probability table:
##  
##              bull_calves
## mass_corr_exp          no         yes
##           no  0.004761905 0.996296296
##           yes 0.995238095 0.003703704
## 
##   Parameters of node mass_sul_exp (multinomial distribution)
## 
## Conditional probability table:
##  
##             Farm
## mass_sul_exp           A           B           C           D           E
##          no  0.003597122 0.003184713 0.004854369 0.004132231 0.990909091
##          yes 0.996402878 0.996815287 0.995145631 0.995867769 0.009090909
##             Farm
## mass_sul_exp           F
##          no  0.996551724
##          yes 0.003448276
## 
##   Parameters of node feed_lane_cleaning (multinomial distribution)
## 
## Conditional probability table:
##  
##                   titanium
## feed_lane_cleaning          no         yes
##      scrape/lagoon 0.997487437 0.012195122
##      clean         0.002512563 0.987804878
## 
##   Parameters of node no_classes (multinomial distribution)
## 
## Conditional probability table:
##  
##           Farm
## no_classes           A           B           C           D           E
##          0 0.606714628 0.116772824 0.119741100 0.101928375 0.987878788
##          1 0.390887290 0.460721868 0.702265372 0.498622590 0.006060606
##          2 0.002398082 0.422505308 0.177993528 0.399449036 0.006060606
##           Farm
## no_classes           F
##          0 0.333333333
##          1 0.498850575
##          2 0.167816092
```

```
set.seed(117)
cpquery(mhaemfarm.bayes, (enr == "R"), pinkeye == "yes")
```

```
## [1] 0.622962
```

```
cpquery(mhaemfarm.bayes, (enr == "R"), pinkeye == "no")
```

```
## [1] 0.962188
```

```
cpquery(mhaemfarm.bayes, (enr == "S"), pinkeye == "yes")
```

```
## [1] 0.3785272
```

```
cpquery(mhaemfarm.bayes, (enr == "S"), pinkeye == "no")
```

```
## [1] 0.04271263
```

Animal level query

```
# fit the parameters - bayesian method
mhaemind.bayes = bn.fit(mhaemind.avg, mhaemind.analyze, method = "bayes")

# probability table
mhaemind.bayes
```

```
## 
##   Bayesian network parameters
## 
##   Parameters of node Farm (multinomial distribution)
## 
## Conditional probability table:
##  
##     season
## Farm    summer    winter
##    A 0.1099099 0.3081081
##    B 0.2900901 0.1819820
##    C 0.1459459 0.1639640
##    D 0.2360360 0.1279279
##    F 0.2180180 0.2180180
## 
##   Parameters of node season (multinomial distribution)
## 
## Conditional probability table:
##  summer winter 
##    0.5    0.5 
## 
##   Parameters of node brd (multinomial distribution)
## 
## Conditional probability table:
##       neg      pos 
## 0.454955 0.545045 
## 
##   Parameters of node dan (multinomial distribution)
## 
## Conditional probability table:
##  
##    Farm
## dan           A           B           C           D           F
##   S 0.090517241 0.080152672 0.180232558 0.995049505 0.334710744
##   R 0.909482759 0.919847328 0.819767442 0.004950495 0.665289256
## 
##   Parameters of node enr (multinomial distribution)
## 
## Conditional probability table:
##  
## , , tild = S
## 
##    dan
## enr           S           R
##   S 0.996240602 0.005617978
##   R 0.003759398 0.994382022
## 
## , , tild = R
## 
##    dan
## enr           S           R
##   S 0.500000000 0.002347418
##   R 0.500000000 0.997652582
## 
## 
##   Parameters of node flr (multinomial distribution)
## 
## Conditional probability table:
##  
## , , tul = S
## 
##    Farm
## flr           A           B           C           D           F
##   S 0.997835498 0.996688742 0.852112676 0.300995025 0.996183206
##   R 0.002164502 0.003311258 0.147887324 0.699004975 0.003816794
## 
## , , tul = R
## 
##    Farm
## flr           A           B           C           D           F
##   S 0.500000000 0.184684685 0.103960396 0.500000000 0.545045045
##   R 0.500000000 0.815315315 0.896039604 0.500000000 0.454954955
## 
## 
##   Parameters of node gam (multinomial distribution)
## 
## Conditional probability table:
##  
##    Farm
## gam           A           B           C           D           F
##   S 0.995689655 0.576335878 0.354651163 0.995049505 0.293388430
##   R 0.004310345 0.423664122 0.645348837 0.004950495 0.706611570
## 
##   Parameters of node pen (multinomial distribution)
## 
## Conditional probability table:
##  
##    Farm
## pen          A          B          C          D          F
##   S 0.86637931 0.72900763 0.47093023 0.05445545 0.91322314
##   R 0.13362069 0.27099237 0.52906977 0.94554455 0.08677686
## 
##   Parameters of node spc (multinomial distribution)
## 
## Conditional probability table:
##  
##    Farm
## spc           A           B           C           D           F
##   S 0.909482759 0.576335878 0.761627907 0.995049505 0.995867769
##   R 0.090517241 0.423664122 0.238372093 0.004950495 0.004132231
## 
##   Parameters of node tet (multinomial distribution)
## 
## Conditional probability table:
##  
## , , flr = S
## 
##    enr
## tet           S           R
##   S 0.697530864 0.002392344
##   R 0.302469136 0.997607656
## 
## , , flr = R
## 
##    enr
## tet           S           R
##   S 0.078947368 0.005154639
##   R 0.921052632 0.994845361
## 
## 
##   Parameters of node tild (multinomial distribution)
## 
## Conditional probability table:
##  
##     gam
## tild          S          R
##    S 0.75874126 0.03164557
##    R 0.24125874 0.96835443
## 
##   Parameters of node tilm (multinomial distribution)
## 
## Conditional probability table:
##  
## , , tild = S
## 
##     pen
## tilm           S           R
##    S 0.932000000 0.541237113
##    R 0.068000000 0.458762887
## 
## , , tild = R
## 
##     pen
## tilm           S           R
##    S 0.003184713 0.007692308
##    R 0.996815287 0.992307692
## 
## 
##   Parameters of node tul (multinomial distribution)
## 
## Conditional probability table:
##  
##    gam
## tul           S           R
##   S 0.996503497 0.183544304
##   R 0.003496503 0.816455696
## 
##   Parameters of node total_abx (multinomial distribution)
## 
## Conditional probability table:
##  
##            Farm
## total_abx            A          B          C          D          F
##   0         0.64942529 0.11704835 0.12015504 0.10231023 0.33333333
##   1 to 2    0.30459770 0.23155216 0.46899225 0.44884488 0.20936639
##   3 or more 0.04597701 0.65139949 0.41085271 0.44884488 0.45730028
## 
##   Parameters of node last_treat (multinomial distribution)
## 
## Conditional probability table:
##  
##             total_abx
## last_treat             0      1 to 2   3 or more
##   <16 days   0.002747253 0.172169811 0.178308824
##   16-60 days 0.002747253 0.172169811 0.420955882
##   >=60 days  0.035714286 0.653301887 0.398897059
##   no trx     0.958791209 0.002358491 0.001838235
## 
##   Parameters of node days_since_pen (multinomial distribution)
## 
## Conditional probability table:
##  
##               Farm
## days_since_pen           A           B           C           D           F
##   treated      0.004310345 0.423664122 0.412790698 0.004950495 0.004132231
##   no treatment 0.995689655 0.576335878 0.587209302 0.995049505 0.995867769
## 
##   Parameters of node days_since_ceph (multinomial distribution)
## 
## Conditional probability table:
##  
##                Farm
## days_since_ceph           A           B           C           D           F
##    treated      0.047413793 0.003816794 0.005813953 0.054455446 0.334710744
##    no treatment 0.952586207 0.996183206 0.994186047 0.945544554 0.665289256
## 
##   Parameters of node days_since_mac (multinomial distribution)
## 
## Conditional probability table:
##  
##               total_abx
## days_since_mac           0      1 to 2   3 or more
##   treated      0.005494505 0.089622642 0.466911765
##   no treatment 0.994505495 0.910377358 0.533088235
## 
##   Parameters of node days_since_phen (multinomial distribution)
## 
## Conditional probability table:
##  
## , , days_since_pen = treated
## 
##                last_treat
## days_since_phen    <16 days  16-60 days   >=60 days      no trx
##    0-60 days    0.788617886 0.695473251 0.013333333 0.333333333
##    >=60 days    0.203252033 0.004115226 0.013333333 0.333333333
##    no treatment 0.008130081 0.300411523 0.973333333 0.333333333
## 
## , , days_since_pen = no treatment
## 
##                last_treat
## days_since_phen    <16 days  16-60 days   >=60 days      no trx
##    0-60 days    0.771689498 0.399449036 0.001064963 0.001430615
##    >=60 days    0.114155251 0.465564738 0.640042599 0.001430615
##    no treatment 0.114155251 0.134986226 0.358892439 0.997138770
## 
## 
##   Parameters of node days_since_tet (multinomial distribution)
## 
## Conditional probability table:
##  
##               Farm
## days_since_tet           A           B           C           D           F
##   treated      0.004310345 0.003816794 0.005813953 0.648514851 0.086776860
##   no treatment 0.995689655 0.996183206 0.994186047 0.351485149 0.913223140
## 
##   Parameters of node days_since_flr (multinomial distribution)
## 
## Conditional probability table:
##  
##               last_treat
## days_since_flr    <16 days  16-60 days   >=60 days      no trx
##   0-60 days    0.356725146 0.320132013 0.001972387 0.002849003
##   >=60 days    0.076023392 0.161716172 0.262327416 0.002849003
##   no treatment 0.567251462 0.518151815 0.735700197 0.994301994
```

# *3. H. somni*

### Farm level factors

#### 3.1 Data Wrangling

```
# import data
hsom.df <- read_csv("HSOM.csv", show_col_types = FALSE)

# remove na rows
hsom.df <- filter(hsom.df, rowSums(is.na(hsom.df)) != ncol(hsom.df))
```

```
# connect calf treatment variables to hsom df
hsom.df <- hsom.df %>%
  mutate(calf_ID = ID) %>%
  select(-"ID")

hsom.raw <- left_join(hsom.df, calf.trx, by = "calf_ID")


# make variables factors, add labels
hsom.factor = hsom.raw
  
hsom.factor$season <- factor(hsom.raw$season, order = FALSE,
                              levels = c(1, 2),
                              labels = c("summer", "winter"))
hsom.factor$brd <- factor(hsom.raw$brd, order = FALSE,
                          levels = c(0, 1),
                          labels = c("neg", "pos"))
hsom.factor$cef <- factor(hsom.raw$cef, order = FALSE,
                           levels = c(0, 1),
                           labels = c("S", "R"))
hsom.factor$dan <- factor(hsom.raw$dan, order = FALSE,
                           levels = c(0, 1),
                           labels = c("S", "R"))
hsom.factor$enr <- factor(hsom.raw$enr, order = FALSE,
                           levels = c(0, 1),
                           labels = c("S", "R"))
hsom.factor$flr <- factor(hsom.raw$flr, order = FALSE,
                         levels = c(0, 1),
                         labels = c("S", "R"))
hsom.factor$gam <- factor(hsom.raw$gam, order = FALSE,
                           levels = c(0, 1),
                           labels = c("S", "R"))
hsom.factor$gen <- factor(hsom.raw$gen, order = FALSE,
                           levels = c(0, 1),
                           labels = c("S", "R"))
hsom.factor$pen <- factor(hsom.raw$pen, order = FALSE,
                           levels = c(0, 1),
                           labels = c("S", "R"))
hsom.factor$spc <- factor(hsom.raw$spc, order = FALSE,
                           levels = c(0, 1),
                           labels = c("S", "R"))
hsom.factor$tet <- factor(hsom.raw$tet, order = FALSE,
                           levels = c(0, 1),
                           labels = c("S", "R"))
hsom.factor$tild <- factor(hsom.raw$tild, order = FALSE,
                            levels = c(0, 1),
                            labels = c("S", "R"))
hsom.factor$tilm <- factor(hsom.raw$tilm, order = FALSE,
                            levels = c(0, 1),
                            labels = c("S", "R"))
hsom.factor$tul <- factor(hsom.raw$tul, order = FALSE,
                           levels = c(0, 1),
                           labels = c("S", "R"))
hsom.factor$source_no <- factor(hsom.raw$source_no, order = FALSE,
                                 levels = c(1, 2, 3),
                                 labels = c("single", "low multi", "high multi"))
hsom.factor$onsite_milking <- factor(hsom.raw$onsite_milking, order = FALSE,
                                      levels = c(0, 1),
                                      labels = c("no", "yes"))
hsom.factor$resp_vacc <- factor(hsom.raw$resp_vacc, order = FALSE,
                                 levels = c(0, 1),
                                 labels = c("no", "yes"))
hsom.factor$salmonella <- factor(hsom.raw$salmonella, order = FALSE,
                           levels = c(0, 1),
                           labels = c("no", "yes"))
hsom.factor$bovishield <- factor(hsom.raw$bovishield, order = FALSE,
                                  levels = c(0, 1),
                                  labels = c("no", "yes"))
hsom.factor$pyramid <- factor(hsom.raw$pyramid, order = FALSE,
                               levels = c(0, 1),
                               labels = c("no", "yes"))
hsom.factor$titanium <- factor(hsom.raw$titanium, order = FALSE,
                                levels = c(0, 1),
                                labels = c("no", "yes"))
hsom.factor$pinkeye <- factor(hsom.raw$pinkeye, order = FALSE,
                               levels = c(0, 1),
                               labels = c("no", "yes"))
hsom.factor$clostrid <- factor(hsom.raw$clostrid, order = FALSE,
                                levels = c(0, 1),
                                labels = c("no", "yes"))
hsom.factor$colost_source <- factor(hsom.raw$colost_source, order = FALSE,
                                     levels = c(1, 2),
                                     labels = c("p", "dp"))
hsom.factor$colost_past <- factor(hsom.raw$colost_past, order = FALSE,
                                   levels = c(0, 1),
                                   labels = c("no", "yes"))
hsom.factor$colost_again <- factor(hsom.raw$colost_again, order = FALSE,
                                    levels = c(0, 1, 99),
                                    labels = c("no", "yes", "-99"))
hsom.factor$hosp_milk_pastuerized <- factor(hsom.raw$hosp_milk_pastuerized, 
                                            order = FALSE,
                                             levels = c(0, 1),
                                             labels = c("no", "yes"))
hsom.factor$action <- factor(hsom.raw$action, order = FALSE,
                              levels = c(1, 2),
                              labels = c("screen", "premium"))
hsom.factor$bull_calves <- factor(hsom.raw$bull_calves, order = FALSE,
                                   levels = c(0, 1),
                                   labels = c("no", "yes"))
hsom.factor$milk_mass_med <- factor(hsom.raw$milk_mass_med, order = FALSE,
                                     levels = c(0, 1),
                                     labels = c("no", "yes"))
hsom.factor$mass_pen_exp <- factor(hsom.raw$mass_pen_exp, order = FALSE,
                                    levels = c(0, 1),
                                    labels = c("no", "yes"))
hsom.factor$mass_tet_exp <- factor(hsom.raw$mass_tet_exp, order = FALSE,
                                    levels = c(0, 1),
                                    labels = c("no", "yes"))
hsom.factor$mass_corr_exp <- factor(hsom.raw$mass_corr_exp, order = FALSE,
                                    levels = c(0, 1),
                                    labels = c("no", "yes"))
hsom.factor$mass_rum_exp <- factor(hsom.raw$mass_rum_exp, order = FALSE,
                                    levels = c(0, 1),
                                    labels = c("no", "yes"))
hsom.factor$mass_sul_exp <- factor(hsom.raw$mass_sul_exp, order = FALSE,
                                    levels = c(0, 1),
                                    labels = c("no", "yes"))
hsom.factor$feed_lane_cleaning <- factor(hsom.raw$feed_lane_cleaning, order = FALSE,
                                          levels = c(1, 2, 3, 4),
                                          labels = c("scrape", "lagoon", "clean", "sl"))
hsom.factor$dust_mgmt <- factor(hsom.raw$dust_mgmt, order = FALSE,
                                 levels = c(0, 1),
                                 labels = c("no", "yes"))
hsom.factor$mort_rate <- factor(hsom.raw$mort_rate, order = FALSE,
                                 levels = c(0, 1),
                                 labels = c("no", "yes"))
hsom.factor$pen_trx_rate <- factor(hsom.raw$pen_trx_rate, order = FALSE,
                                    levels = c(1, 2),
                                    labels = c("low", "high"))
hsom.factor$cef_trx_rate <- factor(hsom.raw$cef_trx_rate, order = FALSE,
                                    levels = c(1, 2),
                                    labels = c("low", "high"))
hsom.factor$mac_trx_rate <- factor(hsom.raw$mac_trx_rate, order = FALSE,
                                    levels = c(1, 2),
                                    labels = c("low", "high"))
hsom.factor$res_nuf_trx_rate <- factor(hsom.raw$res_nuf_trx_rate, order = FALSE,
                                        levels = c(1, 2),
                                        labels = c("low", "high"))
hsom.factor$tet_inj_use <- factor(hsom.raw$tet_inj_use, order = FALSE,
                                  levels = c(0, 1),
                                  labels = c("no", "yes"))
hsom.factor$sul_trx_rate <- factor(hsom.raw$sul_trx_rate, order = FALSE,
                                    levels = c(1, 2),
                                    labels = c("low", "high"))
hsom.factor$bay_trx_rate <- factor(hsom.raw$bay_trx_rate, order = FALSE,
                                    levels = c(1, 2),
                                    labels = c("low", "high"))
hsom.factor$farm <- factor(hsom.raw$farm, order = FALSE,
                            levels = c(1, 2, 3, 4, 5, 6),
                            labels = c("A", "B", "C", "D", "E", "F"))
hsom.factor$last_treat <- factor(hsom.raw$last_treat, order = FALSE,
                                  levels = c(1, 2, 3, 4),
                                  labels = c("<16 days", "16-60 days", ">=60 days", "no trx"))
hsom.factor$no_classes <- factor(hsom.raw$no_classes, order = FALSE,
                                  levels = c(0, 1, 2),
                                  labels = c("0", "1", "2 or more"))
hsom.factor$total_abx <- factor(hsom.raw$total_abx, order = FALSE,
                                  levels = c(0, 1, 2),
                                  labels = c("0", "1 to 2", "3 or more"))
hsom.factor$days_since_pen <- factor(hsom.raw$days_since_pen, order = FALSE,
                                      levels = c(1, 2, 3, 4),
                                      labels = c("<16 days", "16-60 days", ">=60 days", 
                                                 "no trx"))
hsom.factor$days_since_ceph <- factor(hsom.raw$days_since_ceph, order = FALSE,
                                       levels = c(1, 2, 3, 4),
                                       labels = c("<16 days", "16-60 days", ">=60 days", 
                                                  "no trx"))
hsom.factor$days_since_mac <- factor(hsom.raw$days_since_mac, order = FALSE,
                                      levels = c(1, 2, 3, 4),
                                      labels = c("<16 days", "16-60 days", ">=60 days", 
                                                 "no trx"))
hsom.factor$days_since_phen <- factor(hsom.raw$days_since_phen, order = FALSE,
                                       levels = c(1, 2, 3, 4),
                                       labels = c("<16 days", "16-60 days", ">=60 days", 
                                                  "no trx"))
hsom.factor$days_since_tet <- factor(hsom.raw$days_since_tet, order = FALSE,
                                      levels = c(2, 3, 4),
                                      labels = c("16-60 days", ">=60 days", "no trx"))
hsom.factor$days_since_sul <- factor(hsom.raw$days_since_sul, order = FALSE,
                                      levels = c(3, 4),
                                      labels = c(">=60 days", "no trx"))
hsom.factor$days_since_flr <- factor(hsom.raw$days_since_flr, order = FALSE,
                                      levels = c(1, 2, 3, 4),
                                      labels = c("treated", "treated", ">=60 days", "no trx"))

# glimpse(hsom.factor)
```

```
# analytic data frame for all farms
hsom.all <- hsom.factor %>%
            mutate(Farm = farm) %>%
            select(-"farm", -"srp", -"entervene", -"pen_trx_rate", -"cef_trx_rate", 
                   -"mac_trx_rate", -"res_nuf_trx_rate", -"brd",
                   -"tet_inj_use", -"sul_trx_rate", -"bay_trx_rate", -"mort_rate", 
                   - "calf_ID", -"Farm_ID", -"cef", -"dan", -"enr", -"flr", -"total_abx", 
                   -"last_treat", -"days_since_pen", -"days_since_ceph", -"colost_again",
                   -"days_since_mac", -"days_since_phen", -"days_since_tet", 
                   -"days_since_sul",  -"days_since_flr", -"gen")

# check for missingness
summary(aggr(hsom.all))
```

```
## 
##  Missings per variable: 
##               Variable Count
##                 season     0
##                    gam     0
##                    pen     0
##                    spc     0
##                    tet     0
##                   tild     0
##                   tilm     0
##                    tul     0
##              source_no     0
##         onsite_milking     0
##              resp_vacc     0
##             salmonella     0
##             bovishield     0
##                pyramid     0
##               titanium     0
##                pinkeye     0
##               clostrid     0
##          colost_source     0
##            colost_past     0
##  hosp_milk_pastuerized     0
##                 action     0
##            bull_calves     0
##          milk_mass_med     0
##           mass_pen_exp     0
##           mass_tet_exp     0
##           mass_rum_exp     0
##          mass_corr_exp     0
##           mass_sul_exp     0
##     feed_lane_cleaning     0
##              dust_mgmt     0
##             no_classes     0
##                   Farm     0
## 
##  Missings in combinations of variables: 
##                                                     Combinations Count Percent
##  0:0:0:0:0:0:0:0:0:0:0:0:0:0:0:0:0:0:0:0:0:0:0:0:0:0:0:0:0:0:0:0    97     100
```

```
# check cell counts - All Farms
summary(hsom.all) # gam, pen, tul, mass_tet_exp, no_classes(3 or more) below 10
```

```
##     season   gam    pen    spc    tet    tild   tilm   tul         source_no 
##  summer:43   S:93   S:94   S:29   S:36   S:82   S:86   S:93   single    :23  
##  winter:54   R: 4   R: 3   R:68   R:61   R:15   R:11   R: 4   low multi :22  
##                                                               high multi:52  
##                                                                              
##                                                                              
##                                                                              
##  onsite_milking resp_vacc salmonella bovishield pyramid  titanium pinkeye 
##  no :31         no :32    no :54     no :43     no :69   no :82   no :43  
##  yes:66         yes:65    yes:43     yes:54     yes:28   yes:15   yes:54  
##                                                                           
##                                                                           
##                                                                           
##                                                                           
##  clostrid colost_source colost_past hosp_milk_pastuerized     action  
##  no :82   p :64         no :32      no :32                screen :77  
##  yes:15   dp:33         yes:65      yes:65                premium:20  
##                                                                       
##                                                                       
##                                                                       
##                                                                       
##  bull_calves milk_mass_med mass_pen_exp mass_tet_exp mass_rum_exp mass_corr_exp
##  no :63      no :46        no :86       no : 8       no :11       no :34       
##  yes:34      yes:51        yes:11       yes:89       yes:86       yes:63       
##                                                                                
##                                                                                
##                                                                                
##                                                                                
##  mass_sul_exp feed_lane_cleaning dust_mgmt     no_classes Farm  
##  no :40       scrape:31          no :32    0        :41   A:11  
##  yes:57       lagoon:19          yes:65    1        :35   B:11  
##               clean :15                    2 or more:21   C:20  
##               sl    :32                                   D:15  
##                                                           E:32  
##                                                           F: 8
```

```
# remove variables
hsomall.analyze <- as.data.frame(hsom.all)
hsomall.analyze <- as.data.frame(hsom.all) %>%
                    select(-"gam", -"pen", - "tul", 
                           -"mass_tet_exp") %>%
            droplevels(hsomall.analyze$no_classes, exclude = "3 or more")

# check
summary(hsomall.analyze)
```

```
##     season   spc    tet    tild   tilm        source_no  onsite_milking
##  summer:43   S:29   S:36   S:82   S:86   single    :23   no :31        
##  winter:54   R:68   R:61   R:15   R:11   low multi :22   yes:66        
##                                          high multi:52                 
##                                                                        
##                                                                        
##                                                                        
##  resp_vacc salmonella bovishield pyramid  titanium pinkeye  clostrid
##  no :32    no :54     no :43     no :69   no :82   no :43   no :82  
##  yes:65    yes:43     yes:54     yes:28   yes:15   yes:54   yes:15  
##                                                                     
##                                                                     
##                                                                     
##                                                                     
##  colost_source colost_past hosp_milk_pastuerized     action   bull_calves
##  p :64         no :32      no :32                screen :77   no :63     
##  dp:33         yes:65      yes:65                premium:20   yes:34     
##                                                                          
##                                                                          
##                                                                          
##                                                                          
##  milk_mass_med mass_pen_exp mass_rum_exp mass_corr_exp mass_sul_exp
##  no :46        no :86       no :11       no :34        no :40      
##  yes:51        yes:11       yes:86       yes:63        yes:57      
##                                                                    
##                                                                    
##                                                                    
##                                                                    
##  feed_lane_cleaning dust_mgmt     no_classes Farm  
##  scrape:31          no :32    0        :41   A:11  
##  lagoon:19          yes:65    1        :35   B:11  
##  clean :15                    2 or more:21   C:20  
##  sl    :32                                   D:15  
##                                              E:32  
##                                              F: 8
```

```
# create levels
hsom.bl.all = tiers2blacklist(list("season", "Farm",
                     c("spc", "tet", "tild", "tilm",
                       "source_no", "onsite_milking", "resp_vacc", 
                       "salmonella", "bovishield", "pyramid", "titanium", "pinkeye", 
                       "clostrid", "colost_source", "colost_past", 
                       "hosp_milk_pastuerized", "action", "bull_calves", "milk_mass_med", 
                       "mass_pen_exp", "mass_rum_exp", "mass_corr_exp", "mass_sul_exp",
                       "feed_lane_cleaning", "dust_mgmt", "no_classes")))
hsom.bl.all = rbind(hsom.bl.all, c("season"))
hsom.bl.all
```

```
##       from                    to      
##  [1,] "Farm"                  "season"
##  [2,] "spc"                   "season"
##  [3,] "tet"                   "season"
##  [4,] "tild"                  "season"
##  [5,] "tilm"                  "season"
##  [6,] "source_no"             "season"
##  [7,] "onsite_milking"        "season"
##  [8,] "resp_vacc"             "season"
##  [9,] "salmonella"            "season"
## [10,] "bovishield"            "season"
## [11,] "pyramid"               "season"
## [12,] "titanium"              "season"
## [13,] "pinkeye"               "season"
## [14,] "clostrid"              "season"
## [15,] "colost_source"         "season"
## [16,] "colost_past"           "season"
## [17,] "hosp_milk_pastuerized" "season"
## [18,] "action"                "season"
## [19,] "bull_calves"           "season"
## [20,] "milk_mass_med"         "season"
## [21,] "mass_pen_exp"          "season"
## [22,] "mass_rum_exp"          "season"
## [23,] "mass_corr_exp"         "season"
## [24,] "mass_sul_exp"          "season"
## [25,] "feed_lane_cleaning"    "season"
## [26,] "dust_mgmt"             "season"
## [27,] "no_classes"            "season"
## [28,] "spc"                   "Farm"  
## [29,] "tet"                   "Farm"  
## [30,] "tild"                  "Farm"  
## [31,] "tilm"                  "Farm"  
## [32,] "source_no"             "Farm"  
## [33,] "onsite_milking"        "Farm"  
## [34,] "resp_vacc"             "Farm"  
## [35,] "salmonella"            "Farm"  
## [36,] "bovishield"            "Farm"  
## [37,] "pyramid"               "Farm"  
## [38,] "titanium"              "Farm"  
## [39,] "pinkeye"               "Farm"  
## [40,] "clostrid"              "Farm"  
## [41,] "colost_source"         "Farm"  
## [42,] "colost_past"           "Farm"  
## [43,] "hosp_milk_pastuerized" "Farm"  
## [44,] "action"                "Farm"  
## [45,] "bull_calves"           "Farm"  
## [46,] "milk_mass_med"         "Farm"  
## [47,] "mass_pen_exp"          "Farm"  
## [48,] "mass_rum_exp"          "Farm"  
## [49,] "mass_corr_exp"         "Farm"  
## [50,] "mass_sul_exp"          "Farm"  
## [51,] "feed_lane_cleaning"    "Farm"  
## [52,] "dust_mgmt"             "Farm"  
## [53,] "no_classes"            "Farm"  
## [54,] "season"                "season"
```

3.2 BNA

```
# all farms hc structure learning
hsomfarm.hc <- hc(hsomall.analyze, blacklist = hsom.bl.all, 
                  whitelist = wl, score = "aic")

# plot customization
hsom.a <- Rgraphviz::layoutGraph(bnlearn::as.graphNEL(hsomfarm.hc))
graph::nodeRenderInfo(hsom.a) <- list(fontsize=50)
Rgraphviz::renderGraph(hsom.a)
```

```
# all farms tabu structure learning
hsomfarm.tabu <- tabu(hsomall.analyze, blacklist = hsom.bl.all, whitelist = wl, score = "aic")

# plot customization
hsom.b <- Rgraphviz::layoutGraph(bnlearn::as.graphNEL(hsomfarm.tabu))
graph::nodeRenderInfo(hsom.b) <- list(fontsize=50)
Rgraphviz::renderGraph(hsom.b)
```

```
graphviz.compare(hsomfarm.hc, hsomfarm.tabu, shape = "ellipse", 
                 main= c("Hsom Farms HC DAG", "Hsom Farms Tabu DAG"))
```

```
# bootstrap
hsomfarm.boot = boot.strength(hsomall.analyze, R = 10000, algorithm = "hc", 
                        algorithm.args = list(blacklist = hsom.bl.all, 
                                              whitelist = wl, score = "aic"))

# take arcs with strength of 50% and return average consensus network
hsomfarm.avg = averaged.network(hsomfarm.boot, threshold = 0.5)

# graph customization
hsom.c <- Rgraphviz::layoutGraph(bnlearn::as.graphNEL(hsomfarm.avg))
graph::nodeRenderInfo(hsom.c) <- list(fontsize=50)
Rgraphviz::renderGraph(hsom.c)
```

```
# plot the bootstrapped averaged plot with the original for comparison
graphviz.compare(hsomfarm.avg, hsomfarm.hc, shape = "ellipse", 
                 main= c("Hsom Farm Level Factors Averaged DAG", 
                         "Hsom Farm Level Factors Single DAG"))
```

### Animal level factors

#### 3.3 Data Wrangling

```
# analytic data frame for individual risk factors
hsom.ind <- hsom.factor %>%
            mutate(Farm = farm) %>%
            select("Farm", "season", "brd", "dan", "enr", "flr", "gam", "pen", "spc", 
                   "tet", "tild", "tilm", "tul", "total_abx", "last_treat", 
                   "days_since_pen", "days_since_ceph", "days_since_mac", "days_since_phen",
                   "days_since_tet", "days_since_sul",  "days_since_flr")

# check for missingness
summary(aggr(hsom.ind))
```

```
## 
##  Missings per variable: 
##         Variable Count
##             Farm     0
##           season     0
##              brd     0
##              dan     0
##              enr     0
##              flr     0
##              gam     0
##              pen     0
##              spc     0
##              tet     0
##             tild     0
##             tilm     0
##              tul     0
##        total_abx    32
##       last_treat    32
##   days_since_pen    32
##  days_since_ceph    32
##   days_since_mac    32
##  days_since_phen    32
##   days_since_tet    32
##   days_since_sul    32
##   days_since_flr    32
## 
##  Missings in combinations of variables: 
##                                 Combinations Count  Percent
##  0:0:0:0:0:0:0:0:0:0:0:0:0:0:0:0:0:0:0:0:0:0    65 67.01031
##  0:0:0:0:0:0:0:0:0:0:0:0:0:1:1:1:1:1:1:1:1:1    32 32.98969
```

```
# check cell counts - All Farms
summary(hsom.ind) # -dan, -enr, -flr, -gam, -pen, -tul, total_abx(<=2, 3 or more), last_treat(0-60 days, >=60 days, none), days_since_pen(pen yes/no), -days_since_ceph, days_since_mac(mac yes, no), days_since_phen(0-60 days, >=60 days, no treatment), days_since_tet(0-60 days, >=60 days, no treatment), -days_since_sul, days_since_flr(flr yes/no)
```

```
##  Farm      season    brd     dan    enr    flr    gam    pen    spc    tet   
##  A:11   summer:43   neg:46   S:97   S:97   S:97   S:93   S:94   S:29   S:36  
##  B:11   winter:54   pos:51   R: 0   R: 0   R: 0   R: 4   R: 3   R:68   R:61  
##  C:20                                                                        
##  D:15                                                                        
##  E:32                                                                        
##  F: 8                                                                        
##  tild   tilm   tul        total_abx       last_treat    days_since_pen
##  S:82   S:86   S:93   0        : 8   <16 days  : 9   <16 days  : 4    
##  R:15   R:11   R: 4   1 to 2   :27   16-60 days:17   16-60 days: 6    
##                       3 or more:30   >=60 days :30   >=60 days : 8    
##                       NA's     :32   no trx    : 9   no trx    :47    
##                                      NA's      :32   NA's      :32    
##                                                                       
##    days_since_ceph    days_since_mac   days_since_phen    days_since_tet
##  <16 days  : 0     <16 days  : 0     <16 days  : 4     16-60 days: 1    
##  16-60 days: 2     16-60 days: 2     16-60 days: 8     >=60 days :12    
##  >=60 days : 3     >=60 days :16     >=60 days :26     no trx    :52    
##  no trx    :60     no trx    :47     no trx    :27     NA's      :32    
##  NA's      :32     NA's      :32     NA's      :32                      
##                                                                         
##    days_since_sul   days_since_flr
##  >=60 days: 7     treated  : 6    
##  no trx   :58     >=60 days:11    
##  NA's     :32     no trx   :48    
##                   NA's     :32    
##                                   
##
```

```
# remove variables
hsomind2 <- as.data.frame(hsom.ind) %>%
                    select(-"dan", -"enr", -"flr", -"gam", -"pen", -"tul",
                           -"days_since_ceph", -"days_since_sul")

# check
summary(hsomind2)
```

```
##  Farm      season    brd     spc    tet    tild   tilm       total_abx 
##  A:11   summer:43   neg:46   S:29   S:36   S:82   S:86   0        : 8  
##  B:11   winter:54   pos:51   R:68   R:61   R:15   R:11   1 to 2   :27  
##  C:20                                                    3 or more:30  
##  D:15                                                    NA's     :32  
##  E:32                                                                  
##  F: 8                                                                  
##       last_treat    days_since_pen    days_since_mac   days_since_phen
##  <16 days  : 9   <16 days  : 4     <16 days  : 0     <16 days  : 4    
##  16-60 days:17   16-60 days: 6     16-60 days: 2     16-60 days: 8    
##  >=60 days :30   >=60 days : 8     >=60 days :16     >=60 days :26    
##  no trx    : 9   no trx    :47     no trx    :47     no trx    :27    
##  NA's      :32   NA's      :32     NA's      :32     NA's      :32    
##                                                                       
##     days_since_tet   days_since_flr
##  16-60 days: 1     treated  : 6    
##  >=60 days :12     >=60 days:11    
##  no trx    :52     no trx   :48    
##  NA's      :32     NA's     :32    
##                                    
##
```

```
# recategorize feed_lane_cleaning
hsomind2$total_abx <- factor(hsom.raw$total_abx, order = FALSE,
                                          levels = c(0, 1, 2),
                                          labels = c(">=2", ">=2", 
                                                     "3 or more"))
hsomind2$last_treat <- factor(hsom.raw$last_treat, order = FALSE,
                                          levels = c(1, 2, 3, 4),
                                          labels = c("<60 days", "<60 days", 
                                                     ">=60 days", "no treatment"))
hsomind2$days_since_pen <- factor(hsom.raw$days_since_pen, order = FALSE,
                                          levels = c(1, 2, 3, 4),
                                          labels = c("treated", "treated", 
                                                     "treated", "no treatment"))
hsomind2$days_since_mac <- factor(hsom.raw$days_since_mac, order = FALSE,
                                          levels = c(1, 2, 3, 4),
                                          labels = c("treated", "treated", 
                                                     "treated", "no treatment"))
hsomind2$days_since_phen <- factor(hsom.raw$days_since_phen, order = FALSE,
                                          levels = c(1, 2, 3, 4),
                                          labels = c("<60 days", "<60 days", 
                                                     ">=60 days", "no treatment"))
hsomind2$days_since_tet <- factor(hsom.raw$days_since_tet, order = FALSE,
                                          levels = c(2, 3, 4),
                                          labels = c("treated", "treated", 
                                                     "no treatment"))
hsomind2$days_since_flr <- factor(hsom.raw$days_since_flr, order = FALSE,
                                          levels = c(1, 2, 3, 4),
                                          labels = c("treated", "treated", 
                                                     "treated", "no treatment"))
summary(hsomind2)
```

```
##  Farm      season    brd     spc    tet    tild   tilm       total_abx 
##  A:11   summer:43   neg:46   S:29   S:36   S:82   S:86   >=2      :35  
##  B:11   winter:54   pos:51   R:68   R:61   R:15   R:11   3 or more:30  
##  C:20                                                    NA's     :32  
##  D:15                                                                  
##  E:32                                                                  
##  F: 8                                                                  
##         last_treat      days_since_pen      days_since_mac     days_since_phen
##  <60 days    :26   treated     :18     treated     :18     <60 days    :12    
##  >=60 days   :30   no treatment:47     no treatment:47     >=60 days   :26    
##  no treatment: 9   NA's        :32     NA's        :32     no treatment:27    
##  NA's        :32                                           NA's        :32    
##                                                                               
##                                                                               
##       days_since_tet      days_since_flr
##  treated     :13     treated     :17    
##  no treatment:52     no treatment:48    
##  NA's        :32     NA's        :32    
##                                         
##                                         
##
```

```
# remove Farm E, tilmicosin, last_treatment
hsomind2$Farm <- factor(hsom.raw$farm, order = FALSE,
                            levels = c(1, 2, 3, 4, 6),
                            labels = c("A", "B", "C", "D", "F"))
summary(hsomind2)
```

```
##    Farm       season    brd     spc    tet    tild   tilm       total_abx 
##  A   :11   summer:43   neg:46   S:29   S:36   S:82   S:86   >=2      :35  
##  B   :11   winter:54   pos:51   R:68   R:61   R:15   R:11   3 or more:30  
##  C   :20                                                    NA's     :32  
##  D   :15                                                                  
##  F   : 8                                                                  
##  NA's:32                                                                  
##         last_treat      days_since_pen      days_since_mac     days_since_phen
##  <60 days    :26   treated     :18     treated     :18     <60 days    :12    
##  >=60 days   :30   no treatment:47     no treatment:47     >=60 days   :26    
##  no treatment: 9   NA's        :32     NA's        :32     no treatment:27    
##  NA's        :32                                           NA's        :32    
##                                                                               
##                                                                               
##       days_since_tet      days_since_flr
##  treated     :13     treated     :17    
##  no treatment:52     no treatment:48    
##  NA's        :32     NA's        :32    
##                                         
##                                         
##
```

```
hsomind.analyze <- na.omit(hsomind2) %>%
  select(-"tilm", -"last_treat")

# check
summary(hsomind.analyze)
```

```
##  Farm      season    brd     spc    tet    tild       total_abx 
##  A:11   summer:27   neg:29   S:22   S:23   S:50   >=2      :35  
##  B:11   winter:38   pos:36   R:43   R:42   R:15   3 or more:30  
##  C:20                                                           
##  D:15                                                           
##  F: 8                                                           
##       days_since_pen      days_since_mac     days_since_phen
##  treated     :18     treated     :18     <60 days    :12    
##  no treatment:47     no treatment:47     >=60 days   :26    
##                                          no treatment:27    
##                                                             
##                                                             
##       days_since_tet      days_since_flr
##  treated     :13     treated     :17    
##  no treatment:52     no treatment:48    
##                                         
##                                         
##
```

```
hsomind <- hsomind.analyze %>%
  summarise_each(funs())
```

```
# create levels
hsom.bl.ind = tiers2blacklist(list("season", "Farm",
                     c("brd", "spc", "tet", "tild", 
                       "total_abx", "days_since_pen", 
                       "days_since_mac", "days_since_phen",
                       "days_since_tet", "days_since_flr")))
hsom.bl.ind = rbind(hsom.bl.ind, c("season"))
hsom.bl.ind
```

```
##       from              to      
##  [1,] "Farm"            "season"
##  [2,] "brd"             "season"
##  [3,] "spc"             "season"
##  [4,] "tet"             "season"
##  [5,] "tild"            "season"
##  [6,] "total_abx"       "season"
##  [7,] "days_since_pen"  "season"
##  [8,] "days_since_mac"  "season"
##  [9,] "days_since_phen" "season"
## [10,] "days_since_tet"  "season"
## [11,] "days_since_flr"  "season"
## [12,] "brd"             "Farm"  
## [13,] "spc"             "Farm"  
## [14,] "tet"             "Farm"  
## [15,] "tild"            "Farm"  
## [16,] "total_abx"       "Farm"  
## [17,] "days_since_pen"  "Farm"  
## [18,] "days_since_mac"  "Farm"  
## [19,] "days_since_phen" "Farm"  
## [20,] "days_since_tet"  "Farm"  
## [21,] "days_since_flr"  "Farm"  
## [22,] "season"          "season"
```

#### 3.4 BNA

```
# individual factors hc structure learning
hsomind.hc <- hc(hsomind.analyze, blacklist = hsom.bl.ind, whitelist = wl, score = "aic")
hsomind.hc
```

```
## 
##   Bayesian network learned via Score-based methods
## 
##   model:
##    [season][Farm|season][brd|Farm][spc|Farm][total_abx|Farm]
##    [days_since_tet|Farm:season][tet|Farm:brd][tild|season:brd]
##    [days_since_pen|Farm:total_abx][days_since_mac|total_abx]
##    [days_since_phen|total_abx:days_since_pen][days_since_flr|tet:total_abx]
##   nodes:                                 12 
##   arcs:                                  17 
##     undirected arcs:                     0 
##     directed arcs:                       17 
##   average markov blanket size:           3.17 
##   average neighbourhood size:            2.83 
##   average branching factor:              1.42 
## 
##   learning algorithm:                    Hill-Climbing 
##   score:                                 AIC (disc.) 
##   penalization coefficient:              1 
##   tests used in the learning procedure:  264 
##   optimized:                             TRUE
```

```
# plot customization
hsom.d <- Rgraphviz::layoutGraph(bnlearn::as.graphNEL(hsomind.hc))
graph::nodeRenderInfo(hsom.d) <- list(fontsize=40)
Rgraphviz::renderGraph(hsom.d)
```

```
# individual factors tabu structure learning
hsomind.tabu <- tabu(hsomind.analyze, blacklist = hsom.bl.ind, whitelist = wl, score = "aic")
hsomind.tabu
```

```
## 
##   Bayesian network learned via Score-based methods
## 
##   model:
##    [season][Farm|season][brd|Farm][spc|Farm][total_abx|Farm]
##    [days_since_tet|Farm:season][tet|Farm:brd][tild|season:brd]
##    [days_since_pen|Farm:total_abx][days_since_mac|total_abx]
##    [days_since_phen|total_abx:days_since_pen][days_since_flr|tet:total_abx]
##   nodes:                                 12 
##   arcs:                                  17 
##     undirected arcs:                     0 
##     directed arcs:                       17 
##   average markov blanket size:           3.17 
##   average neighbourhood size:            2.83 
##   average branching factor:              1.42 
## 
##   learning algorithm:                    Tabu Search 
##   score:                                 AIC (disc.) 
##   penalization coefficient:              1 
##   tests used in the learning procedure:  484 
##   optimized:                             TRUE
```

```
# plot customization
hsom.e <- Rgraphviz::layoutGraph(bnlearn::as.graphNEL(hsomind.tabu))
graph::nodeRenderInfo(hsom.e) <- list(fontsize=40)
Rgraphviz::renderGraph(hsom.e)
```

```
graphviz.compare(hsomind.hc, hsomind.tabu, shape = "ellipse", 
                 main= c("Hsom Individual Risk Factors HC DAG", 
                         "Hsom Individual Risk Factors Tabu DAG"))
```

```
# bootstrap
hsomind.boot = boot.strength(hsomind.analyze, R = 10000, algorithm = "hc", 
                        algorithm.args = list(blacklist = hsom.bl.ind, 
                                              whitelist = wl, score = "aic"))

# take arcs with strength of 50% and return average consensus network
hsomind.avg = averaged.network(hsomind.boot, threshold = 0.5)

# graph customization
hsom.f <- Rgraphviz::layoutGraph(bnlearn::as.graphNEL(hsomind.avg))
graph::nodeRenderInfo(hsom.f) <- list(fontsize=40)
Rgraphviz::renderGraph(hsom.f)
```

```
# plot the bootstrapped averaged plot with the original for comparison
graphviz.compare(hsomind.avg, hsomind.hc, shape = "ellipse", 
                 main= c("Hsom Individual Factors Averaged DAG", 
                         "Hsom Individual Factors Single DAG"))
```

#### 3.5 Parameter Learning

Farm level query

```
# fit the parameters - bayesian method
hsomfarm.bayes = bn.fit(hsomfarm.avg, hsomall.analyze, method = "bayes")

# probability table
hsomfarm.bayes
```

```
## 
##   Bayesian network parameters
## 
##   Parameters of node season (multinomial distribution)
## 
## Conditional probability table:
##     summer    winter 
## 0.4438776 0.5561224 
## 
##   Parameters of node spc (multinomial distribution)
## 
## Conditional probability table:
##  
##    source_no
## spc      single   low multi  high multi
##   S 0.007142857 0.723880597 0.251592357
##   R 0.992857143 0.276119403 0.748407643
## 
##   Parameters of node tet (multinomial distribution)
## 
## Conditional probability table:
##         S        R 
## 0.372449 0.627551 
## 
##   Parameters of node tild (multinomial distribution)
## 
## Conditional probability table:
##  
## , , bovishield = no
## 
##     season
## tild      summer      winter
##    S 0.938356164 0.440594059
##    R 0.061643836 0.559405941
## 
## , , bovishield = yes
## 
##     season
## tild      summer      winter
##    S 0.995049505 0.995726496
##    R 0.004950495 0.004273504
## 
## 
##   Parameters of node tilm (multinomial distribution)
## 
## Conditional probability table:
##  
##     tild
## tilm          S          R
##    S 0.94848485 0.53225806
##    R 0.05151515 0.46774194
## 
##   Parameters of node source_no (multinomial distribution)
## 
## Conditional probability table:
##  
##             Farm
## source_no              A           B           C           D           E
##   single     0.004975124 0.004975124 0.002754821 0.992673993 0.001727116
##   low multi  0.990049751 0.990049751 0.002754821 0.003663004 0.001727116
##   high multi 0.004975124 0.004975124 0.994490358 0.003663004 0.996545769
##             Farm
## source_no              F
##   single     0.986394558
##   low multi  0.006802721
##   high multi 0.006802721
## 
##   Parameters of node onsite_milking (multinomial distribution)
## 
## Conditional probability table:
##  
##               feed_lane_cleaning
## onsite_milking      scrape      lagoon       clean          sl
##            no  0.996000000 0.006493506 0.008196721 0.003875969
##            yes 0.004000000 0.993506494 0.991803279 0.996124031
## 
##   Parameters of node resp_vacc (multinomial distribution)
## 
## Conditional probability table:
##  
##          feed_lane_cleaning
## resp_vacc      scrape      lagoon       clean          sl
##       no  0.004000000 0.006493506 0.008196721 0.996124031
##       yes 0.996000000 0.993506494 0.991803279 0.003875969
## 
##   Parameters of node salmonella (multinomial distribution)
## 
## Conditional probability table:
##  
##           Farm
## salmonella           A           B           C           D           E
##        no  0.007462687 0.007462687 0.004132231 0.928571429 0.997409326
##        yes 0.992537313 0.992537313 0.995867769 0.071428571 0.002590674
##           Farm
## salmonella           F
##        no  0.989795918
##        yes 0.010204082
## 
##   Parameters of node bovishield (multinomial distribution)
## 
## Conditional probability table:
##  
##           Farm
## bovishield           A           B           C           D           E
##        no  0.007462687 0.007462687 0.995867769 0.994505495 0.002590674
##        yes 0.992537313 0.992537313 0.004132231 0.005494505 0.997409326
##           Farm
## bovishield           F
##        no  0.989795918
##        yes 0.010204082
## 
##   Parameters of node pyramid (multinomial distribution)
## 
## Conditional probability table:
##  
##        Farm
## pyramid           A           B           C           D           E           F
##     no  0.992537313 0.992537313 0.004132231 0.994505495 0.997409326 0.010204082
##     yes 0.007462687 0.007462687 0.995867769 0.005494505 0.002590674 0.989795918
## 
##   Parameters of node titanium (multinomial distribution)
## 
## Conditional probability table:
##  
##         feed_lane_cleaning
## titanium      scrape      lagoon       clean          sl
##      no  0.996000000 0.993506494 0.008196721 0.996124031
##      yes 0.004000000 0.006493506 0.991803279 0.003875969
## 
##   Parameters of node pinkeye (multinomial distribution)
## 
## Conditional probability table:
##  
##        Farm
## pinkeye           A           B           C           D           E           F
##     no  0.992537313 0.007462687 0.004132231 0.005494505 0.997409326 0.010204082
##     yes 0.007462687 0.992537313 0.995867769 0.994505495 0.002590674 0.989795918
## 
##   Parameters of node clostrid (multinomial distribution)
## 
## Conditional probability table:
##  
##         titanium
## clostrid          no         yes
##      no  0.996969697 0.016129032
##      yes 0.003030303 0.983870968
## 
##   Parameters of node colost_source (multinomial distribution)
## 
## Conditional probability table:
##  
##              resp_vacc
## colost_source          no         yes
##            p  0.007692308 0.980916031
##            dp 0.992307692 0.019083969
## 
##   Parameters of node colost_past (multinomial distribution)
## 
## Conditional probability table:
##  
##            resp_vacc
## colost_past          no         yes
##         no  0.992307692 0.003816794
##         yes 0.007692308 0.996183206
## 
##   Parameters of node hosp_milk_pastuerized (multinomial distribution)
## 
## Conditional probability table:
##  
##                      resp_vacc
## hosp_milk_pastuerized          no         yes
##                   no  0.992307692 0.003816794
##                   yes 0.007692308 0.996183206
## 
##   Parameters of node action (multinomial distribution)
## 
## Conditional probability table:
##  
##          Farm
## action              A           B           C           D           E
##   screen  0.992537313 0.992537313 0.004132231 0.994505495 0.997409326
##   premium 0.007462687 0.007462687 0.995867769 0.005494505 0.002590674
##          Farm
## action              F
##   screen  0.989795918
##   premium 0.010204082
## 
##   Parameters of node bull_calves (multinomial distribution)
## 
## Conditional probability table:
##  
##            Farm
## bull_calves           A           B           C           D           E
##         no  0.007462687 0.992537313 0.995867769 0.005494505 0.997409326
##         yes 0.992537313 0.007462687 0.004132231 0.994505495 0.002590674
##            Farm
## bull_calves           F
##         no  0.010204082
##         yes 0.989795918
## 
##   Parameters of node milk_mass_med (multinomial distribution)
## 
## Conditional probability table:
##  
##              Farm
## milk_mass_med           A           B           C           D           E
##           no  0.007462687 0.992537313 0.995867769 0.994505495 0.002590674
##           yes 0.992537313 0.007462687 0.004132231 0.005494505 0.997409326
##              Farm
## milk_mass_med           F
##           no  0.010204082
##           yes 0.989795918
## 
##   Parameters of node mass_pen_exp (multinomial distribution)
## 
## Conditional probability table:
##  
##             Farm
## mass_pen_exp           A           B           C           D           E
##          no  0.007462687 0.992537313 0.995867769 0.994505495 0.997409326
##          yes 0.992537313 0.007462687 0.004132231 0.005494505 0.002590674
##             Farm
## mass_pen_exp           F
##          no  0.989795918
##          yes 0.010204082
## 
##   Parameters of node mass_rum_exp (multinomial distribution)
## 
## Conditional probability table:
##  
##             mass_pen_exp
## mass_rum_exp          no         yes
##          no  0.002890173 0.978260870
##          yes 0.997109827 0.021739130
## 
##   Parameters of node mass_corr_exp (multinomial distribution)
## 
## Conditional probability table:
##  
##              bull_calves
## mass_corr_exp          no         yes
##           no  0.003937008 0.992753623
##           yes 0.996062992 0.007246377
## 
##   Parameters of node mass_sul_exp (multinomial distribution)
## 
## Conditional probability table:
##  
##             Farm
## mass_sul_exp           A           B           C           D           E
##          no  0.007462687 0.007462687 0.004132231 0.005494505 0.997409326
##          yes 0.992537313 0.992537313 0.995867769 0.994505495 0.002590674
##             Farm
## mass_sul_exp           F
##          no  0.989795918
##          yes 0.010204082
## 
##   Parameters of node feed_lane_cleaning (multinomial distribution)
## 
## Conditional probability table:
##  
##                   Farm
## feed_lane_cleaning           A           B           C           D           E
##             scrape 0.988805970 0.003731343 0.993801653 0.002747253 0.001295337
##             lagoon 0.003731343 0.988805970 0.002066116 0.002747253 0.001295337
##             clean  0.003731343 0.003731343 0.002066116 0.991758242 0.001295337
##             sl     0.003731343 0.003731343 0.002066116 0.002747253 0.996113990
##                   Farm
## feed_lane_cleaning           F
##             scrape 0.005102041
##             lagoon 0.984693878
##             clean  0.005102041
##             sl     0.005102041
## 
##   Parameters of node dust_mgmt (multinomial distribution)
## 
## Conditional probability table:
##  
##          resp_vacc
## dust_mgmt          no         yes
##       no  0.992307692 0.003816794
##       yes 0.007692308 0.996183206
## 
##   Parameters of node no_classes (multinomial distribution)
## 
## Conditional probability table:
##  
##            Farm
## no_classes            A           B           C           D           E
##   0         0.363184080 0.184079602 0.002754821 0.069597070 0.996545769
##   1         0.631840796 0.363184080 0.647382920 0.465201465 0.001727116
##   2 or more 0.004975124 0.452736318 0.349862259 0.465201465 0.001727116
##            Farm
## no_classes            F
##   0         0.251700680
##   1         0.496598639
##   2 or more 0.251700680
## 
##   Parameters of node Farm (multinomial distribution)
## 
## Conditional probability table:
##  
##     season
## Farm      summer      winter
##    A 0.208812261 0.038226300
##    B 0.001915709 0.203363914
##    C 0.231800766 0.185015291
##    D 0.070881226 0.221712538
##    E 0.369731801 0.295107034
##    F 0.116858238 0.056574924
```

```
# conditional probabilities
hsomfarm.bayes$tilm
```

```
## 
##   Parameters of node tilm (multinomial distribution)
## 
## Conditional probability table:
##  
##     tild
## tilm          S          R
##    S 0.94848485 0.53225806
##    R 0.05151515 0.46774194
```

```
hsomfarm.bayes$tet
```

```
## 
##   Parameters of node tet (multinomial distribution)
## 
## Conditional probability table:
##         S        R 
## 0.372449 0.627551
```

```
hsomfarm.bayes$spc
```

```
## 
##   Parameters of node spc (multinomial distribution)
## 
## Conditional probability table:
##  
##    source_no
## spc      single   low multi  high multi
##   S 0.007142857 0.723880597 0.251592357
##   R 0.992857143 0.276119403 0.748407643
```

```
hsomfarm.bayes$gen
```

```
## NULL
```

```
set.seed(118)
cpquery(hsomfarm.bayes, (tild =="R"), bovishield == "yes")
```

```
## [1] 0.004460303
```

```
cpquery(hsomfarm.bayes, (tild =="R"), bovishield == "no")
```

```
## [1] 0.3599736
```

```
cpquery(hsomfarm.bayes, (tild =="S"), bovishield == "yes")
```

```
## [1] 0.994639
```

```
cpquery(hsomfarm.bayes, (tild =="S"), bovishield == "no")
```

```
## [1] 0.6594837
```

```
set.seed(119)
cpquery(hsomfarm.bayes, (tilm == "R"), tild == "R")
```

```
## [1] 0.4666247
```

```
cpquery(hsomfarm.bayes, (tilm == "S"), tild == "R")
```

```
## [1] 0.5251656
```

```
cpquery(hsomfarm.bayes, (tild == "R"), tilm == "R")
```

```
## [1] 0.627551
```

```
cpquery(hsomfarm.bayes, (tild == "S"), tilm == "S")
```

```
## [1] 0.9038243
```

```
set.seed(120)
cpquery(hsomfarm.bayes, (tilm == "R"), tild == "R" & bovishield == "yes")
```

```
## [1] 0.4705882
```

```
cpquery(hsomfarm.bayes, (tilm == "R"), tild == "S" & bovishield == "yes")
```

```
## [1] 0.04875887
```

```
cpquery(hsomfarm.bayes, (tilm == "R"), tild == "S" & bovishield == "no")
```

```
## [1] 0.04925939
```

```
cpquery(hsomfarm.bayes, (tilm == "S"), tild == "R" & bovishield == "yes")
```

```
## [1] 0.6538462
```

```
cpquery(hsomfarm.bayes, (tilm == "S"), tild == "S" & bovishield == "no")
```

```
## [1] 0.9498056
```

Animal level query

```
# fit the parameters - bayesian method
hsomind.bayes = bn.fit(hsomind.hc, hsomind.analyze, method = "bayes")

# probability table
hsomind.bayes
```

```
## 
##   Bayesian network parameters
## 
##   Parameters of node Farm (multinomial distribution)
## 
## Conditional probability table:
##  
##     season
## Farm      summer      winter
##    A 0.330909091 0.054545455
##    B 0.003636364 0.288311688
##    C 0.367272727 0.262337662
##    D 0.112727273 0.314285714
##    F 0.185454545 0.080519481
## 
##   Parameters of node season (multinomial distribution)
## 
## Conditional probability table:
##     summer    winter 
## 0.4166667 0.5833333 
## 
##   Parameters of node brd (multinomial distribution)
## 
## Conditional probability table:
##  
##      Farm
## brd             A           B           C           D           F
##   neg 0.455357143 0.008928571 0.648514851 0.401315789 0.621951220
##   pos 0.544642857 0.991071429 0.351485149 0.598684211 0.378048780
## 
##   Parameters of node spc (multinomial distribution)
## 
## Conditional probability table:
##  
##    Farm
## spc           A           B           C           D           F
##   S 0.633928571 0.812500000 0.301980198 0.006578947 0.012195122
##   R 0.366071429 0.187500000 0.698019802 0.993421053 0.987804878
## 
##   Parameters of node tet (multinomial distribution)
## 
## Conditional probability table:
##  
## , , brd = neg
## 
##    Farm
## tet           A           B           C           D           F
##   S 0.205882353 0.500000000 0.614503817 0.008196721 0.794117647
##   R 0.794117647 0.500000000 0.385496183 0.991803279 0.205882353
## 
## , , brd = pos
## 
##    Farm
## tet           A           B           C           D           F
##   S 0.008196721 0.184684685 0.147887324 0.554945055 0.661290323
##   R 0.991803279 0.815315315 0.852112676 0.445054945 0.338709677
## 
## 
##   Parameters of node tild (multinomial distribution)
## 
## Conditional probability table:
##  
## , , brd = neg
## 
##     season
## tild     summer     winter
##    S 0.91509434 0.37692308
##    R 0.08490566 0.62307692
## 
## , , brd = pos
## 
##     season
## tild     summer     winter
##    S 0.99122807 0.81460674
##    R 0.00877193 0.18539326
## 
## 
##   Parameters of node total_abx (multinomial distribution)
## 
## Conditional probability table:
##  
##            Farm
## total_abx             A           B           C           D           F
##   >=2       0.991071429 0.366071429 0.450495050 0.532894737 0.378048780
##   3 or more 0.008928571 0.633928571 0.549504950 0.467105263 0.621951220
## 
##   Parameters of node days_since_pen (multinomial distribution)
## 
## Conditional probability table:
##  
## , , total_abx = >=2
## 
##               Farm
## days_since_pen           A           B           C           D           F
##   treated      0.004504505 0.012195122 0.445054945 0.006172840 0.016129032
##   no treatment 0.995495495 0.987804878 0.554945055 0.993827160 0.983870968
## 
## , , total_abx = 3 or more
## 
##               Farm
## days_since_pen           A           B           C           D           F
##   treated      0.500000000 0.852112676 0.725225225 0.007042254 0.009803922
##   no treatment 0.500000000 0.147887324 0.274774775 0.992957746 0.990196078
## 
## 
##   Parameters of node days_since_mac (multinomial distribution)
## 
## Conditional probability table:
##  
##               total_abx
## days_since_mac       >=2 3 or more
##   treated      0.0915493 0.5000000
##   no treatment 0.9084507 0.5000000
## 
##   Parameters of node days_since_phen (multinomial distribution)
## 
## Conditional probability table:
##  
## , , days_since_pen = treated
## 
##                total_abx
## days_since_phen        >=2  3 or more
##    <60 days     0.01960784 0.35672515
##    >=60 days    0.01960784 0.21637427
##    no treatment 0.96078431 0.42690058
## 
## , , days_since_pen = no treatment
## 
##                total_abx
## days_since_phen        >=2  3 or more
##    <60 days     0.13066667 0.18974359
##    >=60 days    0.35466667 0.74358974
##    no treatment 0.51466667 0.06666667
## 
## 
##   Parameters of node days_since_tet (multinomial distribution)
## 
## Conditional probability table:
##  
## , , season = summer
## 
##               Farm
## days_since_tet           A           B           C           D           F
##   treated      0.005494505 0.500000000 0.004950495 0.983870968 0.009803922
##   no treatment 0.994505495 0.500000000 0.995049505 0.016129032 0.990196078
## 
## , , season = winter
## 
##               Farm
## days_since_tet           A           B           C           D           F
##   treated      0.023809524 0.004504505 0.004950495 0.582644628 0.983870968
##   no treatment 0.976190476 0.995495495 0.995049505 0.417355372 0.016129032
## 
## 
##   Parameters of node days_since_flr (multinomial distribution)
## 
## Conditional probability table:
##  
## , , total_abx = >=2
## 
##               tet
## days_since_flr           S           R
##   treated      0.109756098 0.004950495
##   no treatment 0.890243902 0.995049505
## 
## , , total_abx = 3 or more
## 
##               tet
## days_since_flr           S           R
##   treated      0.311320755 0.702898551
##   no treatment 0.688679245 0.297101449
```

```
hsomind.bayes$tild
```

```
## 
##   Parameters of node tild (multinomial distribution)
## 
## Conditional probability table:
##  
## , , brd = neg
## 
##     season
## tild     summer     winter
##    S 0.91509434 0.37692308
##    R 0.08490566 0.62307692
## 
## , , brd = pos
## 
##     season
## tild     summer     winter
##    S 0.99122807 0.81460674
##    R 0.00877193 0.18539326
```

```
set.seed(121)
cpquery(hsomind.bayes, (tet == "R"), brd == "neg")
```

```
## [1] 0.5433954
```

```
cpquery(hsomind.bayes, (tet == "R"), brd == "pos")
```

```
## [1] 0.715339
```

```
cpquery(hsomind.bayes, (tet == "S"), brd == "neg")
```

```
## [1] 0.4572265
```

```
cpquery(hsomind.bayes, (tet == "S"), brd == "pos")
```

```
## [1] 0.2833515
```

```
set.seed(122)
cpquery(hsomind.bayes, (tet == "R"), brd == "neg" & days_since_phen == "no treatment")
```

```
## [1] 0.5576037
```

```
cpquery(hsomind.bayes, (tet == "R"), brd == "pos" & days_since_phen == "no treatment")
```

```
## [1] 0.7771976
```

```
cpquery(hsomind.bayes, (tet == "S"), brd == "neg" & days_since_phen == "no treatment")
```

```
## [1] 0.4358407
```

```
cpquery(hsomind.bayes, (tet == "S"), brd == "pos" & days_since_phen == "no treatment")
```

```
## [1] 0.2343324
```

```
set.seed(123)
cpquery(hsomind.bayes, (tet == "R"), brd == "neg" & days_since_phen == "<60 days")
```

```
## [1] 0.4766977
```

```
cpquery(hsomind.bayes, (tet == "R"), brd == "pos" & days_since_phen == "<60 days")
```

```
## [1] 0.7255639
```

```
cpquery(hsomind.bayes, (tet == "S"), brd == "neg" & days_since_phen == "<60 days")
```

```
## [1] 0.5037594
```

```
cpquery(hsomind.bayes, (tet == "S"), brd == "pos" & days_since_phen == "<60 days")
```

```
## [1] 0.2771996
```

```
set.seed(124)
cpquery(hsomind.bayes, (tet == "R"), brd == "neg" & days_since_phen == ">=60 days")
```

```
## [1] 0.5581662
```

```
cpquery(hsomind.bayes, (tet == "R"), brd == "pos" & days_since_phen == ">=60 days")
```

```
## [1] 0.652515
```

```
cpquery(hsomind.bayes, (tet == "S"), brd == "neg" & days_since_phen == ">=60 days")
```

```
## [1] 0.4328439
```

```
cpquery(hsomind.bayes, (tet == "S"), brd == "pos" & days_since_phen == ">=60 days")
```

```
## [1] 0.3569147
```

# 4. Summary Statistics

```
# AMR
amr <- rbind.fill(pmult.all, mhaem.all, hsom.all)

summary(amr)
```

```
##  Farm      season      dan        enr        flr      gam       gen     
##  A:47   summer:174   S   : 91   S   : 89   S   :138   S:209   S   :251  
##  B:61   winter:187   R   :173   R   :175   R   :126   R:152   R   : 13  
##  C:58                NA's: 97   NA's: 97   NA's: 97           NA's: 97  
##  D:69                                                                   
##  E:65                                                                   
##  F:61                                                                   
##  pen     spc     tild    tilm    tul          source_no   onsite_milking
##  S:310   S:239   S:173   S:162   S:297   single    :130   no :105       
##  R: 51   R:122   R:188   R:199   R: 64   low multi :108   yes:256       
##                                          high multi:123                 
##                                                                         
##                                                                         
##                                                                         
##  resp_vacc first_def  salmonella bovishield pyramid   titanium  pinkeye  
##  no : 65   no  :211   no :194    no :188    no :242   no :292   no :112  
##  yes:296   yes : 53   yes:167    yes:173    yes:119   yes: 69   yes:249  
##            NA's: 97                                                      
##                                                                          
##                                                                          
##                                                                          
##  clostrid  colost_source colost_past colost_again hosp_milk_pastuerized
##  no :292   p :295        no : 65     no  : 69     no : 65              
##  yes: 69   dp: 66        yes:296     yes : 88     yes:296              
##                                      -99 :107                          
##                                      NA's: 97                          
##                                                                        
##                                                                        
##      action    bull_calves milk_mass_med mass_pen_exp mass_tet_exp mass_rum_exp
##  screen :303   no :184     no :188       no :314      no : 61      no : 47     
##  premium: 58   yes:177     yes:173       yes: 47      yes:300      yes:314     
##                                                                                
##                                                                                
##                                                                                
##                                                                                
##  mass_corr_exp mass_sul_exp feed_lane_cleaning dust_mgmt     no_classes 
##  no :177       no :126      scrape:105         no : 65   0        :124  
##  yes:184       yes:235      lagoon:122         yes:296   1        :146  
##                             clean : 69                   2 or more: 65  
##                             sl    : 65                   2        : 26  
##                                                          3 or more:  0  
##                                                                         
##    cef        tet     
##  S   :119   S   : 52  
##  R   :  0   R   :164  
##  NA's:242   NA's:145  
##                       
##                       
##
```

```
# animal-level
all_calves <- rbind.fill(pmultind.analyze, mhaemind.analyze, hsomind.analyze) %>% 
  select(Farm, brd, total_abx, days_since_pen, days_since_mac, days_since_phen, 
         days_since_flr, days_since_tet, days_since_ceph, tild, tet, spc, pen, 
         flr, tilm, enr, dan, gam, tul) %>% 
  distinct()

summary(all_calves)
```

```
##  Farm    brd          total_abx        days_since_pen      days_since_mac
##  A:33   neg:126   0        : 35   <60 days    : 16    <60 days    : 12   
##  B:58   pos:137   1 to 2   : 63   >=60 days   : 10    >=60 days   : 26   
##  C:57             3 or more:134   no treatment:203    no treatment:185   
##  D:60             >=2      : 31   treated     : 34    treated     : 40   
##  F:55                                                                    
##      days_since_phen      days_since_flr      days_since_tet
##  <60 days    : 36    <60 days    : 13    treatment   : 21   
##  >=60 days   : 94    >=60 days   : 42    no treatment:217   
##  no treatment:110    no treatment:178    treated     : 25   
##  0-60 days   : 23    0-60 days   : 13                       
##                      treated     : 17                       
##      days_since_ceph tild      tet      spc       pen        flr     
##  <60 days    : 10    S:117   S   : 35   S:177   S   : 59   S   :103  
##  >=60 days   : 10    R:146   R   :120   R: 86   R   : 36   R   :100  
##  no treatment:174            NA's:108           NA's:168   NA's: 60  
##  treated     :  9                                                    
##  NA's        : 60                                                    
##    tilm       enr        dan        gam        tul     
##  S   : 56   S   : 73   S   : 74   S   : 87   S   :150  
##  R   :147   R   :130   R   :129   R   :116   R   : 53  
##  NA's: 60   NA's: 60   NA's: 60   NA's: 60   NA's: 60  
##                                                        
##
```

```
summary(pmultind.analyze)
```

```
##  Farm      season    brd     dan    enr    flr    gam    spc    tild   tilm  
##  A:13   summer:61   neg:63   S:51   S:51   S:49   S:37   S:88   S:32   S:29  
##  B:24   winter:60   pos:58   R:70   R:70   R:72   R:84   R:33   R:89   R:92  
##  C:21                                                                        
##  D:34                                                                        
##  F:29                                                                        
##  tul        total_abx       last_treat      days_since_pen     days_since_ceph
##  S:98   0        :21   <16 days  :19   <60 days    :17     <60 days    : 10   
##  R:23   1 to 2   :34   16-60 days:33   >=60 days   :10     >=60 days   : 10   
##         3 or more:66   >=60 days :48   no treatment:94     no treatment:101   
##                        no trx    :21                                          
##                                                                               
##       days_since_mac     days_since_phen      days_since_tet   days_since_sul
##  <60 days    :12     <60 days    :27     treatment   :25     >=60 days: 20   
##  >=60 days   :27     >=60 days   :42     no treatment:96     no trx   :101   
##  no treatment:82     no treatment:52                                         
##                                                                              
##                                                                              
##       days_since_flr
##  <60 days    :14    
##  >=60 days   :29    
##  no treatment:78    
##                     
##
```

```
summary(mhaemind.analyze)
```

```
##  Farm      season    brd     dan    enr    flr    gam    pen    spc    tet   
##  A:23   summer:55   neg:50   S:35   S:34   S:72   S:71   S:70   S:93   S:15  
##  B:26   winter:55   pos:60   R:75   R:76   R:38   R:39   R:40   R:17   R:95  
##  C:17                                                                        
##  D:20                                                                        
##  F:24                                                                        
##  tild   tilm   tul        total_abx       last_treat      days_since_pen
##  S:55   S:42   S:78   0        :30   <16 days  :14   treated     :18    
##  R:55   R:68   R:32   1 to 2   :35   16-60 days:25   no treatment:92    
##                       3 or more:45   >=60 days :42                      
##                                      no trx    :29                      
##                                                                         
##      days_since_ceph      days_since_mac     days_since_phen
##  treated     : 10    treated     :24     0-60 days   :24    
##  no treatment:100    no treatment:86     >=60 days   :34    
##                                          no treatment:52    
##                                                             
##                                                             
##       days_since_tet      days_since_flr
##  treated     :15     0-60 days   :13    
##  no treatment:95     >=60 days   :16    
##                      no treatment:81    
##                                         
##
```

```
summary(hsomind.analyze)
```

```
##  Farm      season    brd     spc    tet    tild       total_abx 
##  A:11   summer:27   neg:29   S:22   S:23   S:50   >=2      :35  
##  B:11   winter:38   pos:36   R:43   R:42   R:15   3 or more:30  
##  C:20                                                           
##  D:15                                                           
##  F: 8                                                           
##       days_since_pen      days_since_mac     days_since_phen
##  treated     :18     treated     :18     <60 days    :12    
##  no treatment:47     no treatment:47     >=60 days   :26    
##                                          no treatment:27    
##                                                             
##                                                             
##       days_since_tet      days_since_flr
##  treated     :13     treated     :17    
##  no treatment:52     no treatment:48    
##                                         
##                                         
##
```
