# Supplementary material for: Antimicrobial Susceptibility in Respiratory Pathogens and Farm and Animal Variables in Weaned California Dairy Heifers: Logistic Regression and Bayesian Network Analyses
Source: Antibiotics (Basel). 2024 Jan 4;13(1):50. doi: 10.3390/antibiotics13010050 (PMC10812578; doi:10.3390/antibiotics13010050)
Supplement: Supplementary file 1 [file antibiotics-13-00050-s001.zip › Supplemenatry Materials Table S1-S3.docx]

**Table S1.1.** *P. multocida* Farm-Level Analysis Conditional Probabilities

Season

Farm Summer Winter

1 0.08508159 0.0950783

2 0.25291375 0.08165548

3 0.15501166 0.13534676

4 0.22494172 0.24272931

5 0.14102564 0.18903803

6 0.14102564 0.25615213

TILD

DAN S R

S 0.93835616 0.19406393

R 0.06164384 0.80593607

DAN

ENR S R

S 0.995495495 0.002762431

R 0.004504505 0.997237569

Farm

GAM 1 2 3 4 5 6

S 0.38607595 0.0862069 0.0511811 0.82195122 0.16896552 0.03714286

R 0.61392405 0.9137931 0.9488189 0.17804878 0.83103448 0.96285714

DAN

SPC S R

S 0.4189189 0.9640884

R 0.5810811 0.0359116

Onsite Milking = "No"

GAM

TILD S R

S 0.18 0.004424779

R 0.82 0.995575221

Onsite Milking = "Yes"

GAM

TILD S R

S 0.996453901 0.001639344

R 0.003546099 0.998360656

TILD

TILM S R

S 0.910958904 0.002283105

R 0.089041096 0.997716895

SPC

TUL S R

S 0.8891403 0.6267606

R 0.1108597 0.3732394

Farm

No. of

Sources 1 2 3 4 5 6

Single 0.004219409 0.002298851 0.002624672 0.996747967 0.002298851 0.996190476

Multi, Low 0.991561181 0.995402299 0.002624672 0.001626016 0.002298851 0.001904762

Multi, High 0.004219409 0.002298851 0.994750656 0.001626016 0.995402299 0.001904762

Feed Lane Cleaning Method

Onsite Milking Scrape Scrape/Lagoon Lagoon Clean

No 0.996350365 0.005154639 0.002347418 0.003649635

Yes 0.003649635 0.994845361 0.997652582 0.996350365

Feed Lane Cleaning Method

Respiratory

Vaccine Scrape Scrape/Lagoon Lagoon Clean

No 0.003649635 0.994845361 0.002347418 0.003649635

Yes 0.996350365 0. 005154639 0.997652582 0.996350365

Farm

Oral

Antibody 1 2 3 4 5 6

No 0.993670886 0.996551724 0.996062992 0.997560976 0.996551724 0.002857143

Yes 0.006329114 0.003448276 0.003937008 0.002439024 0.003448276 0.997142857

Farm

Salmonella

Vaccine 1 2 3 4 5 6

No 0.006329114 0.003448276 0.003937008 0.997560976 0.996551724 0.997142857

Yes 0.993670886 0.996551724 0.996062992 0.002439024 0.003448276 0.002857143

Farm

IRV1 1 2 3 4 5 6

No 0.006329114 0.003448276 0.996062992 0.997560976 0.003448276 0.997142857

Yes 0.993670886 0.996551724 0.003937008 0.002439024 0.996551724 0.002857143

Farm

IRV2 1 2 3 4 5 6

No 0.993670886 0.996551724 0.003937008 0.997560976 0.996551724 0.002857143

Yes 0.006329114 0.003448276 0.996062992 0.002439024 0.003448276 0.997142857

Feed Lane Cleaning Method

IRV3 Scrape Scrape/Lagoon Lagoon Clean

No 0.996350365 0.994845361 0.997652582 0.003649635

Yes 0.003649635 0.005154639 0.002347418 0.996350365

Farm

Pinkeye

Vaccine 1 2 3 4 5 6

No 0.993670886 0.003448276 0.003937008 0.002439024 0.996551724 0.002857143

Yes 0.006329114 0.996551724 0.996062992 0.997560976 0.003448276 0.997142857

IRV3

Clostridial

Vaccine No Yes

No 0.997757848 0.007246377

Yes 0.002242152 0.992753623

Respiratory Vaccine

Colostrum

Source No Yes

Pooled 0.010204082 0.997942387

From Dam 0.989795918 0.002057613

Respiratory Vaccine

Colostrum in

the Past No Yes

No 0.989795918 0.002057613

Yes 0.010204082 0.997942387

Respiratory Vaccine

Hospital Milk

Pasteurized No Yes

No 0.989795918 0.002057613

Yes 0.010204082 0.997942387

Farm

Total Solids

Screening 1 2 3 4 5 6

Screen 0.993670886 0.996551724 0.003937008 0.997560976 0.996551724 0.997142857

Premium 0.006329114 0.003448276 0.996062992 0.002439024 0.003448276 0.002857143

Farm

Bull Calves

Comingled 1 2 3 4 5 6

No 0.006329114 0.996551724 0.996062992 0.002439024 0.996551724 0.002857143

Yes 0.993670886 0.003448276 0.003937008 0.997560976 0.003448276 0.997142857

Farm

Milk

Medicated 1 2 3 4 5 6

No 0.006329114 0.996551724 0.996062992 0.997560976 0.003448276 0.002857143

Yes 0.993670886 0.003448276 0.003937008 0.002439024 0.996551724 0.997142857

Farm

Mass PEN

Exposure 1 2 3 4 5 6

No 0.006329114 0.996551724 0.996062992 0.997560976 0.996551724 0.997142857

Yes 0.993670886 0.003448276 0.003937008 0.002439024 0.003448276 0.002857143

Oral Antibody

Mass TET Exposure No Yes

No 0.002145923 0.991525424

Yes 0.997854077 0.008474576

Mass PEN Exposure

Mass RUM Exposure No Yes

No 0.001886792 0.981481481

Yes 0.998113208 0.018518519

Bull Calves Comingled

Mass CORR Exposure No Yes

No 0.003597122 0.996732026

Yes 0.996402878 0.003267974

Farm

Mass SUL

Exposure 1 2 3 4 5 6

No 0.006329114 0.003448276 0.003937008 0.002439024 0.996551724 0.997142857

Yes 0.993670886 0.996551724 0.996062992 0.997560976 0.003448276 0.002857143

Farm

Feed Lane

Cleaning

Method 1 2 3 4 5 6

Scrape 0.990506329 0.001724138 0.994094488 0.001219512 0.001724138 0.001428571

S/L 0.003164557 0.001724138 0.001968504 0.001219512 0.994827586 0.001428571

Lagoon 0.003164557 0.994827586 0.001968504 0.001219512 0.001724138 0.995714286

Clean 0.003164557 0.001724138 0.001968504 0.996341463 0.001724138 0.001428571

Respiratory Vaccination

Dust Management No Yes

No 0.989795918 0.002057613

Yes 0.010204082 0.997942387

Farm

No. AMD

Classes 1 2 3 4 5 6

0 0.763713080 0.126436782 0.002624672 0.030894309 0.995402299 0.241904762

1 0.232067511 0.374712644 0.664041995 0.382113821 0.002298851 0.584761905

2 or more 0.004219409 0.498850575 0.333333333 0.586991870 0.002298851 0.173333333

Farm

GAM 1 2 3 4 5 6

S 0.38607595 0.08620690 0.05118110 0.82195122 0.16896552 0.03714286

R 0.61392405 0.91379310 0.94881890 0.17804878 0.83103448 0.96285714

Onsite Milking = “No”

GAM

TILD S R

S 0.180000000 0.004424779

R 0.820000000 0.995575221

Onsite Milking = “Yes”

GAM

TILD S R

S 0.996453901 0.001639344

R 0.003546099 0.998360656

TILD

TILM S R

S 0.910958904 0.002283105

R 0.089041096 0.997716895

TILD

DAN S R

S 0.93835616 0.19406393

R 0.06164384 0.80593607

DAN

ENR S R

S 0.995495495 0.002762431

R 0.004504505 0.997237569

DAN

SPC S R

S 0.4189189 0.9640884

R 0.5810811 0.0359116

**Table S1.2.** *P. multocida* Animal-Level Analysis Conditional Probabilities

Season

Farm Hot Cold

1 0.09918699 0.11735537

2 0.29430894 0.10082645

3 0.18048780 0.16694215

4 0.26178862 0.29917355

5 0.16422764 0.31570248

TILD = S

Farm

DAN 1 2 3 4 5

S 0.954545455 0.023809524 0.500000000 0.998220641 0.954545455

R 0.045454545 0.976190476 0.500000000 0.001779359 0.045454545

TILD = R

Farm

DAN 1 2 3 4 5

S 0.004132231 0.047511312 0.428909953 0.008196721 0.393238434

R 0.995867769 0.952488688 0.571090047 0.991803279 0.606761566

DAN

ENR S R

S 0.995145631 0.003546099

R 0.004854369 0.996453901

Season = Hot

Farm

FLR 1 2 3 4 5

S 0.008196721 0.058011050 0.274774775 0.934782609 0.103960396

R 0.991803279 0.941988950 0.725225225 0.065217391 0.896039604

Season = Cold

Farm

FLR 1 2 3 4 5

S 0.147887324 0.500000000 0.103960396 0.997237569 0.316753927

R 0.852112676 0.500000000 0.896039604 0.002762431 0.683246073

Farm

GAM 1 2 3 4 5

S 0.38636364 0.08677686 0.05188679 0.82163743 0.03767123

R 0.61363636 0.91322314 0.94811321 0.17836257 0.96232877

DAN = S

Farm

SPC 1 2 3 4 5

S 0.954545455 0.045454545 0.005494505 0.286476868 0.995867769

R 0.045454545 0.954545455 0.994505495 0.713523132 0.004132231

DAN = R

Farm

SPC 1 2 3 4 5

S 0.995867769 0.954545455 0.830578512 0.991803279 0.997076023

R 0.004132231 0.045454545 0.169421488 0.008196721 0.002923977

GAM

TILD S R

S 0.86000000 0.00295858

R 0.14000000 0.99704142

TILD

TILM S R

S 0.900000000 0.002793296

R 0.100000000 0.997206704

SPC = S

Farm

TUL 1 2 3 4 5

S 0.690839695 0.771493213 0.995049505 0.996453901 0.963917526

R 0.309160305 0.228506787 0.004950495 0.003546099 0.036082474

SPC = R

Farm

TUL 1 2 3 4 5

S 0.500000000 0.023809524 0.004504505 0.997512438 0.500000000

R 0.500000000 0.976190476 0.995495495 0.002487562 0.500000000

Farm

No. AMD

Treatments 1 2 3 4 5

0 0.762626263 0.126721763 0.003144654 0.031189084 0.242009132

1 to 2 0.156565657 0.168044077 0.380503145 0.352826511 0.276255708

3 or more 0.080808081 0.705234160 0.616352201 0.615984405 0.481735160

No. AMD Treatments

Last Treated 0 1 to 2 3 or more

<16 days 0.003906250 0.206310680 0.182160804

16-60 days 0.003906250 0.264563107 0.363065327

>=60 days 0.003906250 0.526699029 0.453517588

Never treated 0.988281250 0.002427184 0.001256281

Farm

Days Since PEN 1 2 3 4 5

<60 days 0.005050505 0.126721763 0.663522013 0.001949318 0.002283105

>=60 days 0.005050505 0.374655647 0.050314465 0.001949318 0.002283105

no treatment 0.989898990 0.498622590 0.286163522 0.996101365 0.995433790

Last Treated

Days since MAC <16 days 16-60 days >=60 days Never treated

<60 days 0.264069264 0.213032581 0.001727116 0.003921569

>=60 days 0.160173160 0.243107769 0.333333333 0.003921569

no treatment 0.575757576 0.543859649 0.664939551 0.992156863

Last Treated = “<16 days”

Season

Days since PHEN Hot Cold

<60 days 0.881278539 0.497942387

>=60 days 0.004566210 0.300411523

no treatment 0.114155251 0.201646091

Last Treated = “16-60 days”

Season

Days since PHEN Hot Cold

<60 days 0.644768856 0.188630491

>=60 days 0.060827251 0.560723514

no treatment 0.294403893 0.250645995

Last Treated = “>=60 days”

Season

Days since PHEN Hot Cold

<60 days 0.001801802 0.001658375

>=60 days 0.563963964 0.638474295

no treatment 0.434234234 0.359867330

Last Treated = “no treatment”

Season

Days since PHEN Hot Cold

<60 days 0.003436426 0.004566210

>=60 days 0.003436426 0.004566210

no treatment 0.993127148 0.990867580

Farm

Days since TET 1 2 3 4 5

Treated 0.007575758 0.004132231 0.004716981 0.587719298 0.174657534

No Treatment 0.992424242 0.995867769 0.995283019 0.412280702 0.825342466

Farm

Days since SUL 1 2 3 4 5

Treated 0.007575758 0.004132231 0.004716981 0.587719298 0.003424658

No Treatment 0.992424242 0.995867769 0.995283019 0.412280702 0.996575342

Season = Hot

Farm

FLR 1 2 3 4 5

S 0.008196721 0.058011050 0.274774775 0.934782609 0.103960396

R 0.991803279 0.941988950 0.725225225 0.065217391 0.896039604

Season = Cold

Farm

FLR 1 2 3 4 5

S 0.147887324 0.500000000 0.103960396 0.997237569 0.316753927

R 0.852112676 0.500000000 0.896039604 0.002762431 0.683246073

Farm

GAM 1 2 3 4 5

S 0.38636364 0.08677686 0.05188679 0.82163743 0.03767123

R 0.61363636 0.91322314 0.94811321 0.17836257 0.96232877

GAM

TILD S R

S 0.86000000 0.00295858

R 0.14000000 0.99704142

TILD

TILM S R

S 0.900000000 0.002793296

R 0.100000000 0.997206704

TILD = S

Farm

DAN 1 2 3 4 5

S 0.954545455 0.023809524 0.500000000 0.998220641 0.954545455

R 0.045454545 0.976190476 0.500000000 0.001779359 0.045454545

TILD = R

Farm

DAN 1 2 3 4 5

S 0.004132231 0.047511312 0.428909953 0.008196721 0.393238434

R 0.995867769 0.952488688 0.571090047 0.991803279 0.606761566

DAN

ENR S R

S 0.995145631 0.003546099

R 0.004854369 0.996453901

DAN = S

Farm

SPC 1 2 3 4 5

S 0.954545455 0.045454545 0.005494505 0.286476868 0.995867769

R 0.045454545 0.954545455 0.994505495 0.713523132 0.004132231

DAN = R

Farm

SPC 1 2 3 4 5

S 0.995867769 0.954545455 0.830578512 0.991803279 0.997076023

R 0.004132231 0.045454545 0.169421488 0.008196721 0.002923977

SPC = S

Farm

TUL 1 2 3 4 5

S 0.690839695 0.771493213 0.995049505 0.996453901 0.963917526

R 0.309160305 0.228506787 0.004950495 0.003546099 0.036082474

SPC = R

Farm

TUL 1 2 3 4 5

S 0.500000000 0.023809524 0.004504505 0.997512438 0.500000000

R 0.500000000 0.976190476 0.995495495 0.002487562 0.500000000

**Table S2.1.** *M. haemolytica* Farm-Level Analysis Conditional Probabilities

Season

Farm Hot Cold

1 0.10055096 0.28711485

2 0.26584022 0.16946779

3 0.13360882 0.15266106

4 0.21625344 0.11904762

5 0.08402204 0.06862745

6 0.19972452 0.20308123

Farm

DAN 1 2 3 4 5 6

S 0.089928058 0.079617834 0.179611650 0.995867769 0.118181818 0.334482759

R 0.910071942 0.920382166 0.820388350 0.004132231 0.881818182 0.665517241

Pinkeye Vaccine = “No”

DAN

ENR S R

S 0.346153846 0.004273504

R 0.653846154 0.995726496

Pinkeye Vaccine = “Yes’

DAN

ENR S R

S 0.996240602 0.002304147

R 0.003759398 0.997695853

PEN = S & TUL = S

Farm

FLR 1 2 3 4 5 6

S 0.998960499 0.998402556 0.797520661 0.980000000 0.993150685 0.998113208

R 0.001039501 0.001597444 0.202479339 0.020000000 0.006849315 0.001886792

PEN = R & TUL = S

Farm

FLR 1 2 3 4 5 6

S 0.993150685 0.989795918 0.989795918 0.263676149 0.993150685 0.989795918

R 0.006849315 0.010204082 0.010204082 0.736323851 0.006849315 0.010204082

PEN = S & TUL = R

Farm

FLR 1 2 3 4 5 6

S 0.500000000 0.334482759 0.335616438 0.500000000 0.980000000 0.545283019

R 0.500000000 0.665517241 0.664383562 0.500000000 0.020000000 0.454716981

PEN = R & TUL = R

Farm

FLR 1 2 3 4 5 6

S 0.500000000 0.004132231 0.002958580 0.500000000 0.010204082 0.500000000

R 0.500000000 0.995867769 0.997041420 0.500000000 0.989795918 0.500000000

Farm

GAM 1 2 3 4 5 6

S 0.996402878 0.576433121 0.354368932 0.995867769 0.445454545 0.293103448

R 0.003597122 0.423566879 0.645631068 0.004132231 0.554545455 0.706896552

Farm

PEN 1 2 3 4 5 6

S 0.86690647 0.72929936 0.47087379 0.05371901 0.44545455 0.91379310

R 0.13309353 0.27070064 0.52912621 0.94628099 0.55454545 0.08620690

TILM

SPC S R

S 0.994252874 0.748366013

R 0.005747126 0.251633987

ENR

TET S R

S 0.44202899 0.01461988

R 0.55797101 0.98538012

GAM = S

ENR

TILD S R

S 0.996240602 0.500000000

R 0.003759398 0.500000000

GAM = R

ENR

TILD S R

S 0.100000000 0.026011561

R 0.900000000 0.973988439

TILD = S

PEN

TILM S R

S 0.932000000 0.541237113

R 0.068000000 0.458762887

TILD = R

PEN

TILM S R

S 0.002890173 0.052941176

R 0.997109827 0.947058824

GAM

TUL S R

S 0.98344371 0.23033708

R 0.01655629 0.76966292

Farm

No. of

Sources 1 2 3 4 5 6

Single 0.002398082 0.002123142 0.003236246 0.994490358 0.006060606 0.995402299

Multi, Low 0.995203837 0.995753715 0.003236246 0.002754821 0.006060606 0.002298851

Multi, High 0.002398082 0.002123142 0.993527508 0.002754821 0.987878788 0.002298851

Farm

Onsite

Milking 1 2 3 4 5 6

No 0.996402878 0.003184713 0.995145631 0.004132231 0.009090909 0.003448276

Yes 0.003597122 0.996815287 0.004854369 0.995867769 0.990909091 0.996551724

Farm

Salmonella

Vaccine 1 2 3 4 5 6

No 0.003597122 0.003184713 0.004854369 0.995867769 0.990909091 0.996551724

Yes 0.996402878 0.996815287 0.995145631 0.004132231 0.009090909 0.003448276

Farm

First

Defense 1 2 3 4 5 6

No 0.996402878 0.996815287 0.995145631 0.995867769 0.990909091 0.003448276

Yes 0.003597122 0.003184713 0.004854369 0.004132231 0.009090909 0.996551724

Farm

IRV1 1 2 3 4 5 6

No 0.003597122 0.003184713 0.995145631 0.995867769 0.009090909 0.996551724

Yes 0.996402878 0.996815287 0.004854369 0.004132231 0.990909091 0.003448276

Farm

IRV2 1 2 3 4 5 6

No 0.996402878 0.996815287 0.004854369 0.995867769 0.990909091 0.003448276

Yes 0.003597122 0.003184713 0.995145631 0.004132231 0.009090909 0.996551724

Farm

IRV3 1 2 3 4 5 6

No 0.996402878 0.996815287 0.995145631 0.004132231 0.990909091 0.996551724

Yes 0.003597122 0.003184713 0.004854369 0.995867769 0.009090909 0.003448276

Farm

Pinkeye

Vaccine 1 2 3 4 5 6

No 0.996402878 0.003184713 0.004854369 0.004132231 0.990909091 0.003448276

Yes 0.003597122 0.996815287 0.995145631 0.995867769 0.009090909 0.996551724

IRV3

Clostridial

Vaccine No Yes

No 0.997487437 0.012195122

Yes 0.002512563 0.987804878

Farm

Total Solids

Screening 1 2 3 4 5 6

Screen 0.996402878 0.996815287 0.004854369 0.995867769 0.990909091 0.996551724

Premium 0.003597122 0.003184713 0.995145631 0.004132231 0.009090909 0.003448276

Farm

Bull Calves

Comingled 1 2 3 4 5 6

No 0.003597122 0.996815287 0.995145631 0.004132231 0.990909091 0.003448276

Yes 0.996402878 0.003184713 0.004854369 0.995867769 0.009090909 0.996551724

Farm

Medicated

Milk 1 2 3 4 5 6

No 0.003597122 0.996815287 0.995145631 0.995867769 0.009090909 0.003448276

Yes 0.996402878 0.003184713 0.004854369 0.004132231 0.990909091 0.996551724

Farm

Mass PEN

Exposure 1 2 3 4 5 6

No 0.003597122 0.996815287 0.995145631 0.995867769 0.990909091 0.996551724

Yes 0.996402878 0.003184713 0.004854369 0.004132231 0.009090909 0.003448276

Oral Antibody

Mass TET

Exposure No Yes

No 0.002617801 0.989795918

Yes 0.997382199 0.010204082

Mass PEN Exposure

Mass RUM

Exposure No Yes

No 0.002590674 0.989361702

Yes 0.997409326 0.010638298

Bull Calves Co-Mingled

Mass CORR

Exposure No Yes

No 0.004761905 0.996296296

Yes 0.995238095 0.003703704

Farm

Mass SUL

Exposure 1 2 3 4 5 6

No 0.003597122 0.003184713 0.004854369 0.004132231 0.990909091 0.996551724

Yes 0.996402878 0.996815287 0.995145631 0.995867769 0.009090909 0.003448276

Feed Lane IRV3

Cleaning Method No Yes

Scrape/Lagoon 0.997487437 0.012195122

Clean 0.002512563 0.987804878

Farm

No. AMD

Classes 1 2 3 4 5 6

0 0.606714628 0.116772824 0.119741100 0.101928375 0.987878788 0.333333333

1 0.390887290 0.460721868 0.702265372 0.498622590 0.006060606 0.498850575

2 0.002398082 0.422505308 0.177993528 0.399449036 0.006060606 0.167816092

**Table S2.2.** *M. haemolytica* Animal-Level Conditional Probabilities

Season

Farm Hot Cold

1 0.1099099 0.3081081

2 0.2900901 0.1819820

3 0.1459459 0.1639640

4 0.2360360 0.1279279

5 0.2180180 0.2180180

Farm

DAN 1 2 3 4 5

S 0.090517241 0.080152672 0.180232558 0.995049505 0.334710744

R 0.909482759 0.919847328 0.819767442 0.004950495 0.665289256

TILD = S

DAN

ENR S R

S 0.996240602 0.005617978

R 0.003759398 0.994382022

TILD = R

DAN

ENR S R

S 0.500000000 0.002347418

R 0.500000000 0.997652582

TUL = S

Farm

FLR 1 2 3 4 5

S 0.997835498 0.996688742 0.852112676 0.300995025 0.996183206

R 0.002164502 0.003311258 0.147887324 0.699004975 0.003816794

TUL = R

Farm

FLR 1 2 3 4 5

S 0.500000000 0.184684685 0.103960396 0.500000000 0.545045045

R 0.500000000 0.815315315 0.896039604 0.500000000 0.454954955

Farm

GAM 1 2 3 4 5

S 0.995689655 0.576335878 0.354651163 0.995049505 0.293388430

R 0.004310345 0.423664122 0.645348837 0.004950495 0.706611570

Farm

PEN 1 2 3 4 5

S 0.86637931 0.72900763 0.47093023 0.05445545 0.91322314

R 0.13362069 0.27099237 0.52906977 0.94554455 0.08677686

Farm

SPC 1 2 3 4 5

S 0.909482759 0.576335878 0.761627907 0.995049505 0.995867769

R 0.090517241 0.423664122 0.238372093 0.004950495 0.004132231

FLR = S

ENR

TET S R

S 0.697530864 0.002392344

R 0.302469136 0.997607656

FLR = R

ENR

TET S R

S 0.078947368 0.005154639

R 0.921052632 0.994845361

GAM

TILD S R

S 0.75874126 0.03164557

R 0.24125874 0.96835443

TILD = S

PEN

TILM S R

S 0.932000000 0.541237113

R 0.068000000 0.458762887

TILD = R

PEN

TILM S R

S 0.003184713 0.007692308

R 0.996815287 0.992307692

GAM

TUL S R

S 0.996503497 0.183544304

R 0.003496503 0.816455696

Farm

Total

AMD 1 2 3 4 5

0 0.64942529 0.11704835 0.12015504 0.10231023 0.33333333

1 to 2 0.30459770 0.23155216 0.46899225 0.44884488 0.20936639

3 or more 0.04597701 0.65139949 0.41085271 0.44884488 0.45730028

Total AMD Treatments

Last Treatment 0 1 to 2 3 or more

<16 days 0.002747253 0.172169811 0.178308824

16-60 days 0.002747253 0.172169811 0.420955882

>=60 days 0.035714286 0.653301887 0.398897059

no treatment 0.958791209 0.002358491 0.001838235

Farm

Days Since

PHEN 1 2 3 4 5

Treated 0.004310345 0.423664122 0.412790698 0.004950495 0.004132231

No Treatment 0.995689655 0.576335878 0.587209302 0.995049505 0.995867769

Farm

Days Since

CEPH 1 2 3 4 5

Treated 0.047413793 0.003816794 0.005813953 0.054455446 0.334710744

No treatment 0.952586207 0.996183206 0.994186047 0.945544554 0.665289256

Total AMD Treatments

Days Since

MAC 0 1 to 2 3 or more

Treated 0.005494505 0.089622642 0.466911765

No Treatment 0.994505495 0.910377358 0.533088235

PEN Treatment = “Yes”

Last Treatment

Days Since

PHEN <16 days 16-60 days >=60 days no treatment

0-60 days 0.788617886 0.695473251 0.013333333 0.333333333

>=60 days 0.203252033 0.004115226 0.013333333 0.333333333

No Treatment 0.008130081 0.300411523 0.973333333 0.333333333

PEN Treatment = “No”

Last Treatment

Days Since

PHEN <16 days 16-60 days >=60 days no treatment

0-60 days 0.771689498 0.399449036 0.001064963 0.001430615

>=60 days 0.114155251 0.465564738 0.640042599 0.001430615

No Treatment 0.114155251 0.134986226 0.358892439 0.997138770

Farm

Days Since

TET 1 2 3 4 5

Treated 0.004310345 0.003816794 0.005813953 0.648514851 0.086776860

No Treatment 0.995689655 0.996183206 0.994186047 0.351485149 0.913223140

Last Treatment

Days Since

TET <16 days 16-60 days >=60 days no treatment

0-60 days 0.356725146 0.320132013 0.001972387 0.002849003

>=60 days 0.076023392 0.161716172 0.262327416 0.002849003

No Treatment 0.567251462 0.518151815 0.735700197 0.994301994

**Table S3.1.** *H. somni* Farm-Level Conditional Probabilities

No. of Source

SPC Single Multi, Low Multi, High

S 0.007142857 0.723880597 0.251592357

R 0.992857143 0.276119403 0.748407643

IRV1 = “No”

Season

TILD Hot Cold

S 0.938356164 0.440594059

R 0.061643836 0.559405941

IRV1 = “Yes”

Season

TILD Hot Cold

S 0.995049505 0.995726496

R 0.004950495 0.004273504

TILD

TILM S R

S 0.94848485 0.53225806

R 0.05151515 0.46774194

Farm

No. of

Sources 1 2 3 4 5 6

Single 0.004975124 0.004975124 0.002754821 0.992673993 0.001727116 0.986394558

Multi, Low 0.990049751 0.990049751 0.002754821 0.003663004 0.001727116 0.006802721

Multi, High 0.004975124 0.004975124 0.994490358 0.003663004 0.996545769 0.006802721

Feed Lane Cleaning Method

Onsite Milking Scrape Scrape/Lagoon Lagoon Clean

No 0.996000000 0.003875969 0.006493506 0.008196721

Yes 0.004000000 0.996124031 0.993506494 0.991803279

Feed Lane Cleaning Method

Respiratory

Vaccine Scrape Scrape/Lagoon Lagoon Clean

No 0.004000000 0.996124031 0.006493506 0.008196721

Yes 0.996000000 0.003875969 0.993506494 0.991803279

Farm

Salmonella

Vaccine 1 2 3 4 5 6

No 0.007462687 0.007462687 0.004132231 0.928571429 0.997409326 0.989795918

Yes 0.992537313 0.992537313 0.995867769 0.071428571 0.002590674 0.010204082

Farm

IRV1 1 2 3 4 5 6

No 0.007462687 0.007462687 0.995867769 0.994505495 0.002590674 0.989795918

Yes 0.992537313 0.992537313 0.004132231 0.005494505 0.997409326 0.010204082

Farm

IRV2 1 2 3 4 5 6

No 0.992537313 0.992537313 0.004132231 0.994505495 0.997409326 0.010204082

Yes 0.007462687 0.007462687 0.995867769 0.005494505 0.002590674 0.989795918

Feed Lane Cleaning Method

IRV3 Scrape Scrape/Lagoon Lagoon Clean

No 0.996000000 0.996124031 0.993506494 0.008196721

Yes 0.004000000 0.003875969 0.006493506 0.991803279

Farm

Pinkeye

Vaccine 1 2 3 4 5 6

No 0.992537313 0.007462687 0.004132231 0.005494505 0.997409326 0.010204082

Yes 0.007462687 0.992537313 0.995867769 0.994505495 0.002590674 0.989795918

IRV3

Clostridial

Vaccine No Yes

No 0.996969697 0.016129032

Yes 0.003030303 0.983870968

Respiratory Vaccine

Colostrum

Source No Yes

Pooled 0.007692308 0.980916031

From Dam 0.992307692 0.019083969

Respiratory Vaccine

Colostrum in

Past No Yes

No 0.992307692 0.003816794

Yes 0.007692308 0.996183206

Respiratory Vaccine

Hospital Milk

Pasteurized No Yes

No 0.992307692 0.003816794

Yes 0.007692308 0.996183206

Farm

Total Solids

Screening 1 2 3 4 5 6

Screen 0.992537313 0.992537313 0.004132231 0.994505495 0.997409326 0.989795918

Premium 0.007462687 0.007462687 0.995867769 0.005494505 0.002590674 0.010204082

Farm

Bull Calves

Comingled 1 2 3 4 5 6

No 0.007462687 0.992537313 0.995867769 0.005494505 0.997409326 0.010204082

Yes 0.992537313 0.007462687 0.004132231 0.994505495 0.002590674 0.989795918

Farm

Medicated

Milk 1 2 3 4 5 6

No 0.007462687 0.992537313 0.995867769 0.994505495 0.002590674 0.010204082

Yes 0.992537313 0.007462687 0.004132231 0.005494505 0.997409326 0.989795918

Farm

Mass PEN

Exposure 1 2 3 4 5 6

No 0.007462687 0.992537313 0.995867769 0.994505495 0.997409326 0.989795918

Yes 0.992537313 0.007462687 0.004132231 0.005494505 0.002590674 0.010204082

Mass PEN Exposure

Mass RUM Exposure No Yes

No 0.002890173 0.978260870

Yes 0.997109827 0.021739130

Bull Calves Comingled

Mass CORR Exposure No Yes

No 0.003937008 0.992753623

Yes 0.996062992 0.007246377

Farm

Mass SUL

Exposure 1 2 3 4 5 6

No 0.007462687 0.007462687 0.004132231 0.005494505 0.997409326 0.989795918

Yes 0.992537313 0.992537313 0.995867769 0.994505495 0.002590674 0.010204082

Feed Lane

Cleaning Farm

Method 1 2 3 4 5 6

Scrape 0.988805970 0.003731343 0.993801653 0.002747253 0.001295337 0.005102041

S/L 0.003731343 0.003731343 0.002066116 0.002747253 0.996113990 0.005102041

Lagoon 0.003731343 0.988805970 0.002066116 0.002747253 0.001295337 0.984693878

Clean 0.003731343 0.003731343 0.002066116 0.991758242 0.001295337 0.005102041

Respiratory Vaccine

Dust Management No Yes

No 0.992307692 0.003816794

Yes 0.007692308 0.996183206

Farm

No. AMD

Classes 1 2 3 4 5 6

0 0.363184080 0.184079602 0.002754821 0.069597070 0.996545769 0.251700680

1 0.631840796 0.363184080 0.647382920 0.465201465 0.001727116 0.496598639

2 or more 0.004975124 0.452736318 0.349862259 0.465201465 0.001727116 0.251700680

Season

Farm Hot Cold

1 0.208812261 0.038226300

2 0.001915709 0.203363914

3 0.231800766 0.185015291

4 0.070881226 0.221712538

5 0.369731801 0.295107034

6 0.116858238 0.056574924

TILD

TILM S R

S 0.94848485 0.53225806

R 0.05151515 0.46774194

No. of Sources

SPC Single Multi, Low Multi, High

S 0.007142857 0.723880597 0.251592357

R 0.992857143 0.276119403 0.748407643

**Table S3.2.** *H. somni* Animal-Level Conditional Probabilities

Season

Farm Hot Cold

1 0.330909091 0.054545455

2 0.003636364 0.288311688

3 0.367272727 0.262337662

4 0.112727273 0.314285714

6 0.185454545 0.080519481

Farm

BRD Status 1 2 3 4 6

Negative 0.455357143 0.008928571 0.648514851 0.401315789 0.621951220

Positive 0.544642857 0.991071429 0.351485149 0.598684211 0.378048780

Farm

SPC 1 2 3 4 6

S 0.633928571 0.812500000 0.301980198 0.006578947 0.012195122

R 0.366071429 0.187500000 0.698019802 0.993421053 0.987804878

BRD Status = Negative

Farm

TET 1 2 3 4 6

S 0.205882353 0.500000000 0.614503817 0.008196721 0.794117647

R 0.794117647 0.500000000 0.385496183 0.991803279 0.205882353

BRD Status = Positive

Farm

TET 1 2 3 4 6

S 0.008196721 0.184684685 0.147887324 0.554945055 0.661290323

R 0.991803279 0.815315315 0.852112676 0.445054945 0.338709677

BRD Status = Negative

Season

TILD Hot Cold

S 0.91509434 0.37692308

R 0.08490566 0.62307692

BRD Status = Positive

Season

TILD Hot Cold

S 0.99122807 0.81460674

R 0.00877193 0.18539326

Farm

Total AMD

Treatments 1 2 3 4 6

>=2 0.991071429 0.366071429 0.450495050 0.532894737 0.378048780

3 or more 0.008928571 0.633928571 0.549504950 0.467105263 0.621951220

Total AMD Treatments = “>=2”

Farm

PHEN

Treatment 1 2 3 4 6

Treated 0.004504505 0.012195122 0.445054945 0.006172840 0.016129032

No Treatment 0.995495495 0.987804878 0.554945055 0.993827160 0.983870968

Total AMD Treatments = “3 or more”

Farm

PHEN Treatment 1 2 3 4 6

Treated 0.500000000 0.852112676 0.725225225 0.007042254 0.009803922

No Treatment 0.500000000 0.147887324 0.274774775 0.992957746 0.990196078

Total AMD Treatments

MAC Treatment >=2 3 or more

Treated 0.0915493 0.5000000

No Treatment 0.9084507 0.5000000

PEN Treatment = “Treated”

Total AMD Treatments

Days Since PHEN >=2 3 or more

<60 days 0.01960784 0.35672515

>=60 days 0.01960784 0.21637427

No Treatment 0.96078431 0.42690058

PEN Treatment = “No Treatment”

Total AMD Treatments

Days Since PHEN >=2 3 or more

<60 days 0.13066667 0.18974359

>=60 days 0.35466667 0.74358974

No Treatmnent 0.51466667 0.06666667

Season = Hot

Farm

TET Treatment 1 2 3 4 6

Treated 0.005494505 0.500000000 0.004950495 0.983870968 0.009803922

No Treatment 0.994505495 0.500000000 0.995049505 0.016129032 0.990196078

Season = Cold

Farm

TET Treatment 1 2 3 4 6

Treated 0.023809524 0.004504505 0.004950495 0.582644628 0.983870968

No Treatment 0.976190476 0.995495495 0.995049505 0.417355372 0.016129032

Total AMD Treatments = “>=2”

TET

FLR Treatment S R

Treated 0.109756098 0.004950495

No Treatment 0.890243902 0.995049505

Total AMD Treatments = “3 or more”

TET

FLR Treatment S R

Treated 0.311320755 0.702898551

No Treatment 0.688679245 0.297101449

BRD Status = Negative

Season

TILD Hot Cold

S 0.91509434 0.37692308

R 0.08490566 0.62307692

BRD Status = Positive

Season

TILD Hot Cold

S 0.99122807 0.81460674

R 0.00877193 0.18539326
