# Supplementary material for: Antimicrobial Susceptibility in Respiratory Pathogens and Farm and Animal Variables in Weaned California Dairy Heifers: Logistic Regression and Bayesian Network Analyses
Source: Antibiotics (Basel). 2024 Jan 4;13(1):50. doi: 10.3390/antibiotics13010050 (PMC10812578; doi:10.3390/antibiotics13010050)
Supplement: Supplementary file 1 [file antibiotics-13-00050-s001.zip › Supplementary File S1.docx]

**Supplementary**

**Supplementary Methods:**

Methods and materials for culture and sensitivity determination

Deep nasopharyngeal swabs (DNPS) were immediately placed in Amies with charcoal transport media (CultureSwab Plus, BD BBL™, COPAN Italia SpA, Brescia Italy) after collection. All samples were stored in a cooler with ice during sampling, followed by refrigeration at ~4ºC for up to 48 h before submission for bacteriological analysis. All swabs were submitted to the California Animal Health and Food Safety laboratory in Davis, CA for selective culture and sensitivity testing. Each DNPS in Amies with charcoal transport media was cultured on sheep blood-3% agar (3% SBA) and chocolate agar (CHOC). Plates were incubated for 48 hours at 35 ± 2˚C in 5-10% CO_2_ (3% SBA, CHOC) and examined every 18-24 hours for colonies of interest. Organisms of interest included the respiratory pathogens *Mannheimia haemolytica*, *Pasteurella multocida*, and *Histophilus somni* recovered from DNPS; All colonies of interest were confirmed by biochemical testing and matrix-assisted, laser desorption-ionization time of flight (MALDI-TOF) mass spectrometry. Antimicrobial susceptibility testing was performed using broth microdilution (Trek Sensititre, Trek Diagnostic Systems, Thermo Fisher Scientific, Waltham, MA) according to Clinical Laboratory Standards Institute (CLSI) guidelines to determine the MIC of the 19 AMD contained on the Sensititre Bovine BOPO7F Vet AST plate (Thermo Scientific, Remel Inc., Lenexa, KS, USA). This panel of 19 AMD was selected to match those monitored by the United States Department of Agriculture, Animal and Plant Health Inspection Service. Where available, interpretive criteria from CLSI-established clinical breakpoints were used to classify an organism as susceptible versus not susceptible (resistant or intermediate). Although there are published CLSI breakpoints for ampicillin and sulfadimethoxine for *P. multocida, M. haemolytica*, and *H. somni*, the current MIC breakpoints are below the lowest concentration of drug tested in the standard broth microdilution method used, and thus susceptibility could not be meaningfully interpreted based on the MIC provided. The suggested epidemiologic cutoff between wild type and evidence of acquired AMR for *P. multocida,* suggested by the European Committee on Antimicrobial Susceptibility Testing, is 1 µg/mL for ampicillin. Isolates were tested against antimicrobials at the following dilutions: ampicillin (0.25-16µg/mL), penicillin (0.12-8 µg/mL), ceftiofur (0.25-8 µg/mL), florfenicol (0.25-8 µg/mL), tylosin (0.5-32 µg/mL), tilmicosin (2-16 µg/mL), tulathromycin (8-64 µg/mL), tildipirosin (2-16 µg/mL), gamithromycin (1-8 µg/mL), tiamulin (0.5-32 µg/mL), clindamycin (0.25-16 µg/mL), danofloxacin (0.12-1 µg/mL), enrofloxacin (0.12-2 µg/mL), trimethoprim-sulfamethoxazole (2/38 µg/mL), sulfadimethoxine (256 µg/mL), tetracycline (0.5-8 µg/mL), gentamicin (1-16 µg/mL), neomycin (8-32 µg/mL), and spectinomycin (8-64 µg/mL). *Escherichia coli* ATTC 25922, *Pseudomonas aeruginosa* ATTC 27853, *Enterococcus faecalis* ATTC 29212, and *Staphylococcus aureus* ATTC 29213 were used as quality control organisms.

Isolates were sub-cultured on SBA (*M. haemolytica, P. multocida, E. coli, Enterococcus* spp*.)* or CHOC (*H. somni)* and incubated for 18-24 hours at 35 ˚C in 5-10% CO_2_. Each isolate was suspended in 0.85% saline to a concentration equivalent to a 0.5 McFarland standard and added to 10ml of cation-adjusted Mueller-Hinton broth containing lysed horse blood (*M. haemolytica,* *P. multocida),* or veterinary fastidious media (*H. somni)* to achieve 5 X 10^5^-1 × 10^6^ cfu/mL. Susceptibility plates were incubated for 18-24 hours at 35 ± 2˚C in ambient air (*M. haemolytica,* *P. multocida),* or 5-10% CO_2_ (*H. somni)* and observed for visible growth. The MIC was determined as the lowest concentration of antimicrobial that prevented growth.

**Figure S1.** Original, unaltered directed acyclic graphs for farm-level and animal-level variable analyses of *Pasteurella multocida, Mannheimia haemolytica,* and *Histophilus somni* isolates tested against tetracycline (TET), tilmicosin (TILM), tildipirosin (TILD), gamithromycin (GAM), enrofloxacin (ENR), danofloxacin (DAN), florfenicol (FLR), spectinomycin (SPC), tulathromycin (TUL), penicillin (PEN), ceftiofur (CEF), and gentamicin (GEN).

Farm-level analysis of *P. multocida*

*
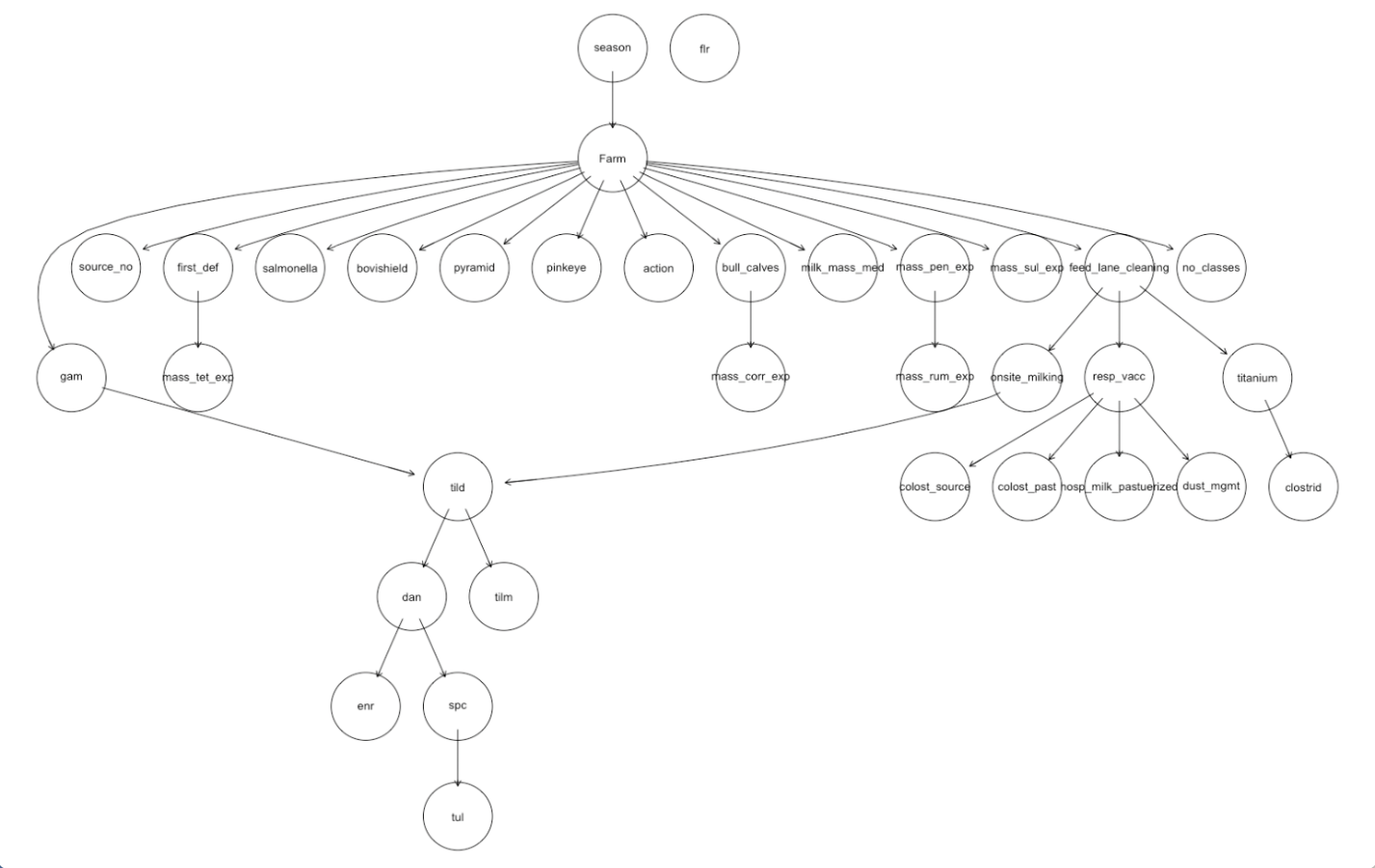
*

Animal-level analysis of *P. multocida*

*
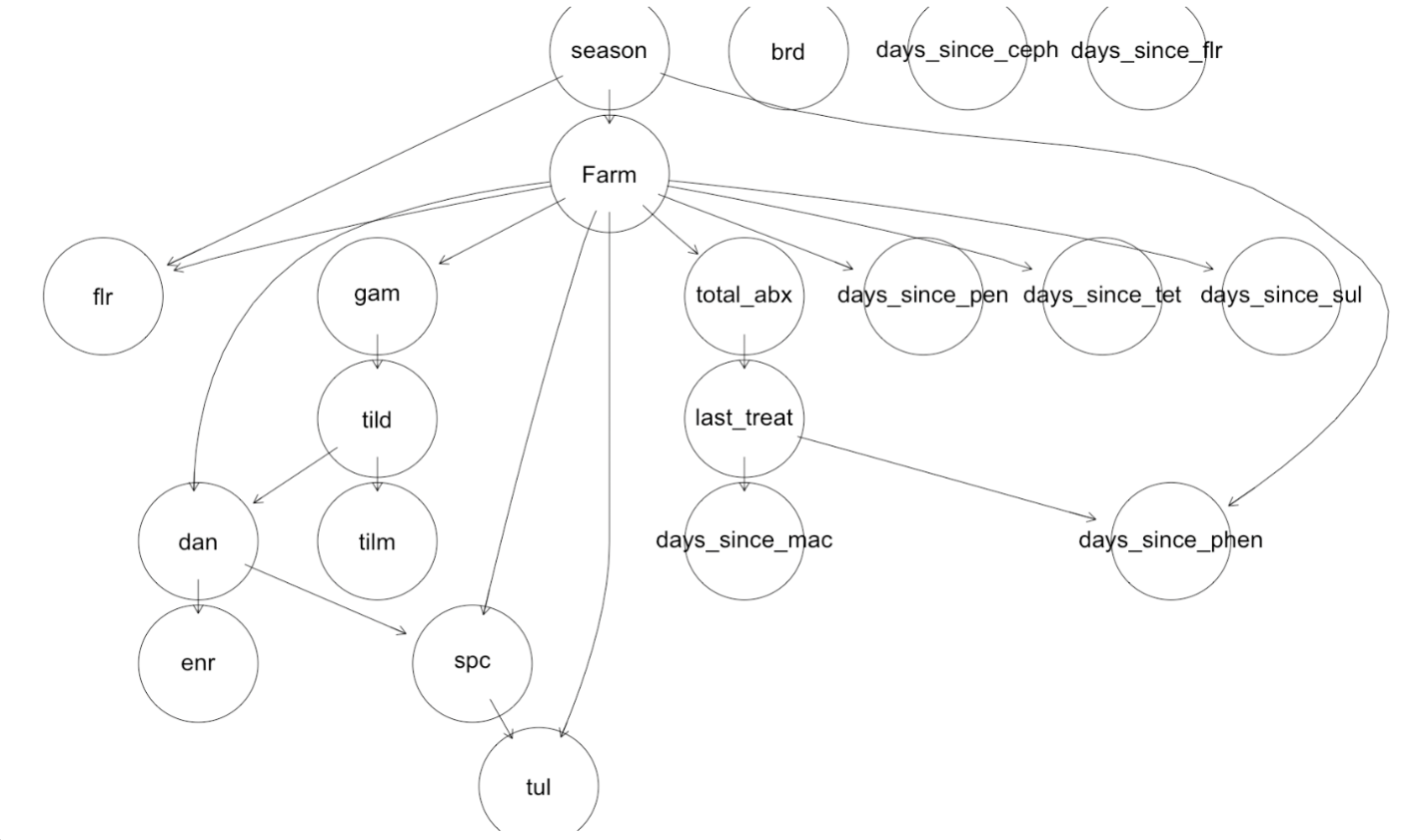
*

Farm-level analysis of *M. haemolytica*

*
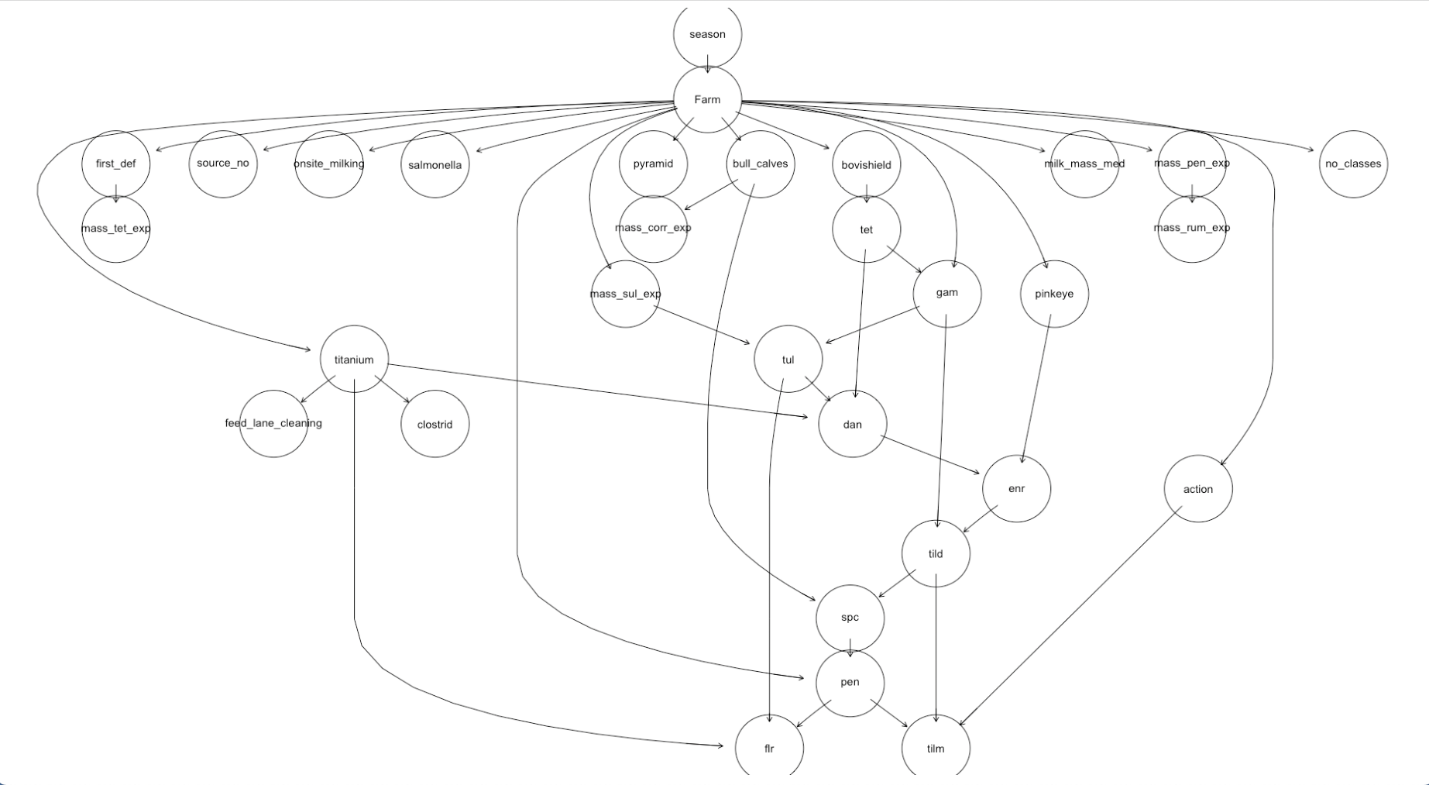
*

Animal-level analysis of *M. haemolytica*

*
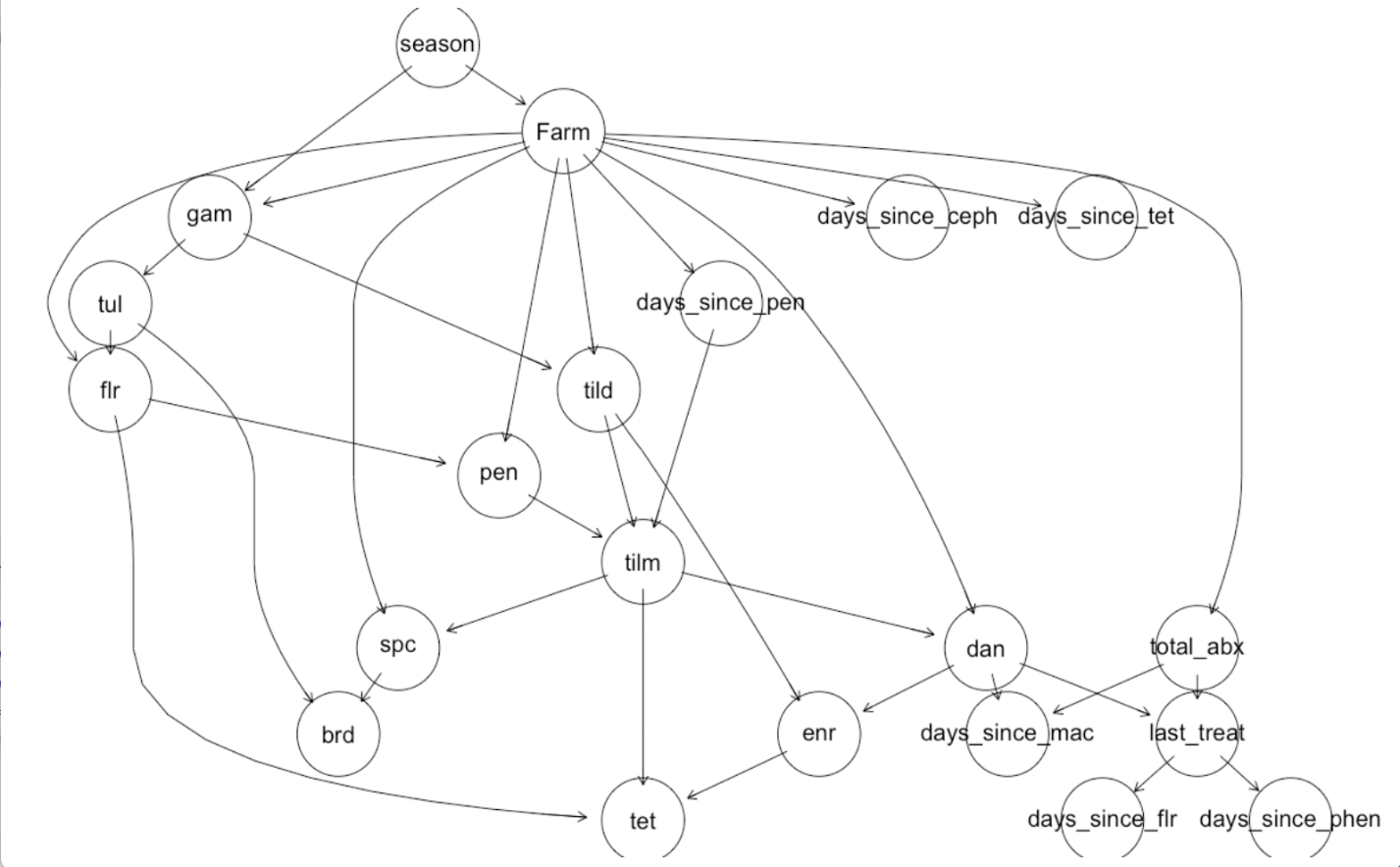
*

Farm-level analysis of *H. somni*

*
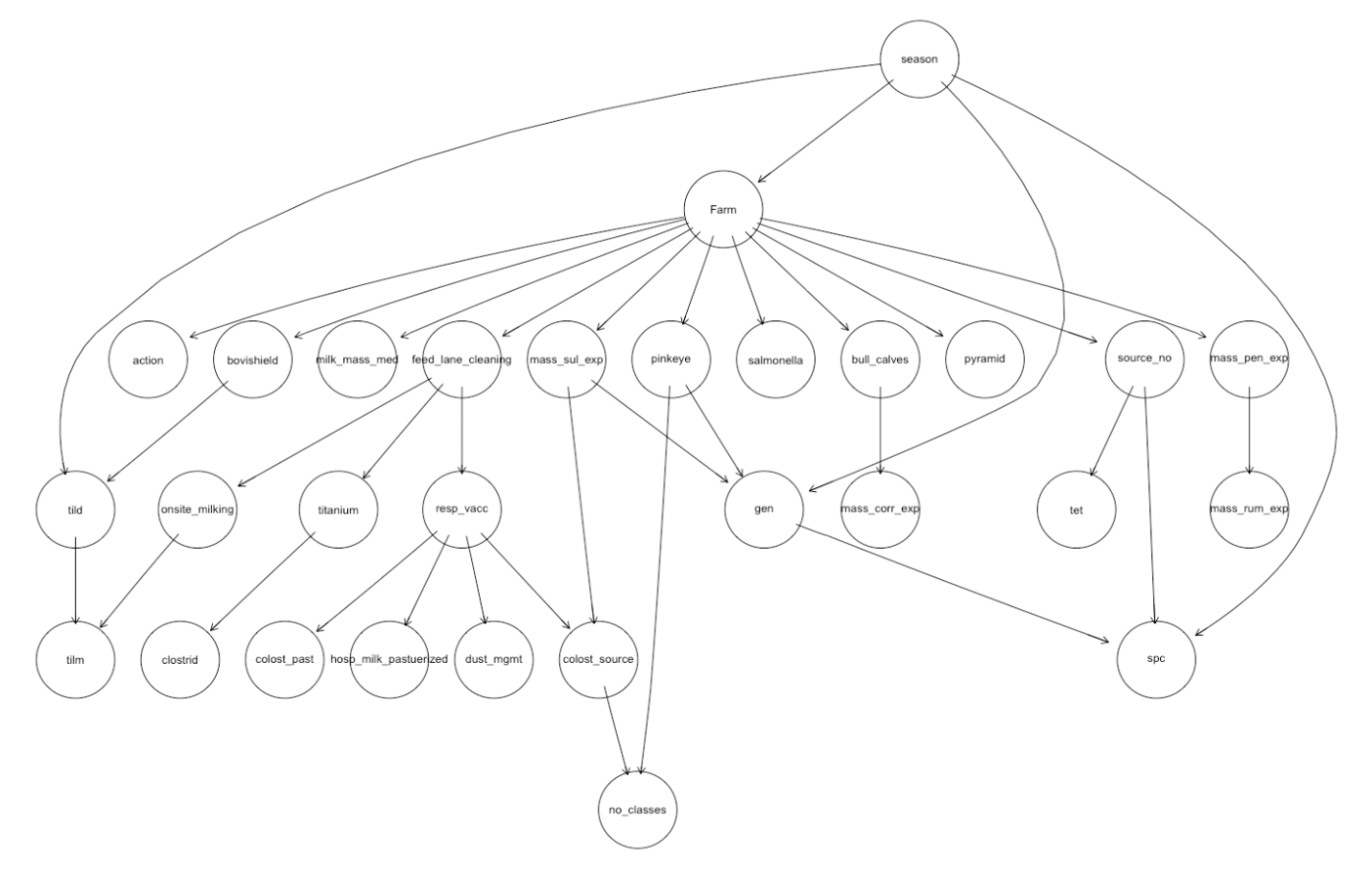
*

Animal-level analysis of *H. somni*

*
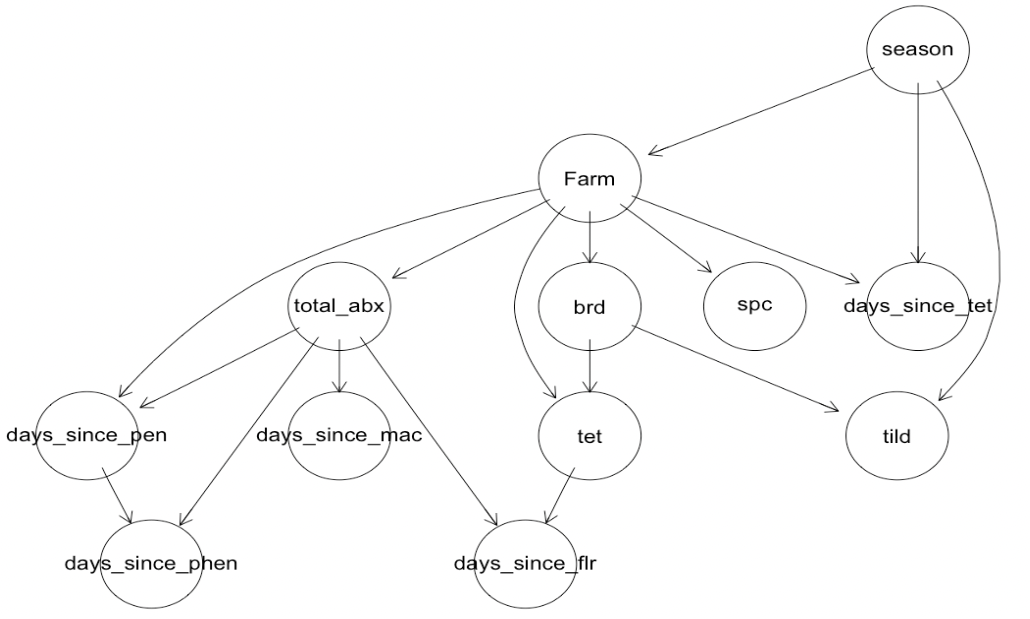
*

**Table S1.1.** Conditional Probabilities for original, unaltered directed acyclic graphs for farm-level variable analyses of *Pasteurella multocida* isolates tested against tetracycline (TET), tilmicosin (TILM), tildipirosin (TILD), gamithromycin (GAM), enrofloxacin (ENR), danofloxacin (DAN), florfenicol (FLR), spectinomycin (SPC), tulathromycin (TUL), penicillin (PEN), ceftiofur (CEF), and gentamicin (GEN).

**Table S1.2.** Conditional Probabilities for original, unaltered directed acyclic graphs for animal-level variable analyses of *Pasteurella multocida* isolates tested against tetracycline (TET), tilmicosin (TILM), tildipirosin (TILD), gamithromycin (GAM), enrofloxacin (ENR), danofloxacin (DAN), florfenicol (FLR), spectinomycin (SPC), tulathromycin (TUL), penicillin (PEN), ceftiofur (CEF), and gentamicin (GEN).

**Table S2.1.** Conditional Probabilities for original, unaltered directed acyclic graphs for farm-level variable analyses of *Mannheimia haemolytica* isolates tested against tetracycline (TET), tilmicosin (TILM), tildipirosin (TILD), gamithromycin (GAM), enrofloxacin (ENR), danofloxacin (DAN), florfenicol (FLR), spectinomycin (SPC), tulathromycin (TUL), penicillin (PEN), ceftiofur (CEF), and gentamicin (GEN).

**Table S2.2.** Conditional Probabilities for original, unaltered directed acyclic graphs for animal-level variable analyses of *Mannheimia haemolytica* isolates tested against tetracycline (TET), tilmicosin (TILM), tildipirosin (TILD), gamithromycin (GAM), enrofloxacin (ENR), danofloxacin (DAN), florfenicol (FLR), spectinomycin (SPC), tulathromycin (TUL), penicillin (PEN), ceftiofur (CEF), and gentamicin (GEN).

**Table S3.1.** Conditional Probabilities for original, unaltered directed acyclic graphs for farm-level variable analyses of *Histophilus somni* isolates tested against tetracycline (TET), tilmicosin (TILM), tildipirosin (TILD), gamithromycin (GAM), enrofloxacin (ENR), danofloxacin (DAN), florfenicol (FLR), spectinomycin (SPC), tulathromycin (TUL), penicillin (PEN), ceftiofur (CEF), and gentamicin (GEN).

**Table S3.2.** Conditional Probabilities for original, unaltered directed acyclic graphs for animal-level variable analyses of *Histophilus somni* isolates tested against tetracycline (TET), tilmicosin (TILM), tildipirosin (TILD), gamithromycin (GAM), enrofloxacin (ENR), danofloxacin (DAN), florfenicol (FLR), spectinomycin (SPC), tulathromycin (TUL), penicillin (PEN), ceftiofur (CEF), and gentamicin (GEN).
